# Molecular Footprints of Quaternary Climate Fluctuations in the Circumpolar Tundra Shrub Dwarf Birch

**DOI:** 10.1111/mec.70082

**Published:** 2025-09-02

**Authors:** Maria Dance, Erin E. Saupe, James Borrell, Pernille Bronken Eidesen, Daniel Ackerman, Jakob Assmann, Bruce C. Forbes, Marina Gurskaya, Toke T. Høye, Stein R. Karlsen, Timo Kumpula, Mariusz Lamentowicz, Michael M. Loranty, Isla Myers‐Smith, Janet Prevéy, Christian Rixen, Gabriela Schaepman‐Strub, Michał Słowiński, Sandra Słowińska, Aleksandr Sokolov, James D. M. Speed, Marcus Spiegel, Martin Wilmking, Marc Macias‐Fauria

**Affiliations:** ^1^ Scott Polar Research Institute University of Cambridge Cambridge UK; ^2^ School of Geography and the Environment University of Oxford Oxford UK; ^3^ The University Centre in Svalbard Longyearbyen Norway; ^4^ Department of Earth Sciences University of Oxford Oxford UK; ^5^ Royal Botanic Gardens Kew Richmond UK; ^6^ University of Minnesota Minneapolis Minnesota USA; ^7^ The University of Edinburgh Edinburgh UK; ^8^ Department of Evolutionary Biology and Environmental Studies University of Zurich Zürich Switzerland; ^9^ Arctic Centre, University of Lapland Rovaniemi Finland; ^10^ Institute of Plant and Animal Ecology, UB RAS Yekaterinburg Russia; ^11^ Department of Ecoscience Aarhus University Aarhus Denmark; ^12^ NORCE Norwegian Research Centre Tromsø Norway; ^13^ Department of Geographical and Historical Studies University of Eastern Finland Kuopio Finland; ^14^ Faculty of Geographical and Geological Sciences Adam Mickiewicz University Poznań Poland; ^15^ Department of Geography Colgate University NY New York New York USA; ^16^ Department of Forest and Conservation Sciences University of British Columbia Vancouver Canada; ^17^ WSL Institute for Snow and Avalanche Research SLF Davos Switzerland; ^18^ Climate Change, Extremes and Natural Hazards in Alpine Regions Research Center CERC Davos Switzerland; ^19^ Institute of Geography and Spatial Organization Polish Academy of Sciences (IGSO PAS) Warsaw Poland; ^20^ Arctic Research Station of the Institute of Plant and Animal Ecology, UB RAS Labytnangi Russia; ^21^ Department of Natural History, NTNU University Museum Norwegian University of Science and Technology Trondheim Norway; ^22^ Institute of Botany and Landscape Ecology University of Greifswald Greifswald Germany

**Keywords:** *Betula glandulosa*, *Betula nana*, demographic history, dwarf birch, phylogeography, Quaternary

## Abstract

The Arctic tundra biome is undergoing rapid shrub expansion (‘shrubification’) in response to anthropogenic climate change. During the previous ~2.6 million years, glacial cycles caused substantial shifts in Arctic vegetation, leading to changes in species' distributions, abundance and connectivity, which have left lasting impacts on the genetic structure of modern populations. Examining how shrubs responded to past climate change through genetic data reveals the demographic dynamics that shaped their current diversity and distribution and sheds light on the resilience of Arctic shrubs. Here we test scenarios of Quaternary demographic history of dwarf birch species ( *Betula nana* L. and *Betula Glandulosa* Michx.) using Single Nucleotide Polymorphism (SNP) markers obtained from RAD sequencing and approximate Bayesian computation. We compare the timings of modelled population events with ice sheet reconstructions and other paleoenvironmental information to untangle the impacts of alternating cold and warm periods on dwarf birch. Our best supported model suggested that the species diverged in the Mid‐Pleistocene Transition as glaciations intensified. We found support for a complex history of inter‐ and intraspecific divergences and gene flow, and secondary contact occurred during both ice sheet expansion and retreat. Our spatiotemporal analysis suggests that the modern genetic structure of dwarf birch results from transitions in climate between glacials and interglacials, with ice sheets acting alternatively as a barrier or an enabler of population mixing. Tundra shrubs may have had more nuanced responses to past climatic changes than phylogeographic analyses have often suggested, with implications for future eco‐evolutionary responses to anthropogenic climate change.

## 1 Introduction

Arctic tundra vegetation covers over 7 million km^2^ of the Earth's surface (Raynolds et al. 2019). Climate warming is contributing to notable changes in the composition of Arctic tundra vegetation, particularly shrub expansion (‘shrubification’) (Myers‐Smith et al. 2011). Shrub expansion has and will have Arctic‐wide impacts on vegetation communities, tundra ecosystems, Arctic surface energy and carbon balance, and potentially global climate feedbacks (Mekonnen et al. 2021). Understanding how Arctic shrubs responded to past climate fluctuations in the Quaternary offers insight into the historical dynamics and resilience of shrub populations and the processes that have resulted in their present distribution and diversity.

The modern Arctic tundra biome formed during the transition from a warmer climate to the onset of Pleistocene glaciations in the Northern Hemisphere ~3–2.6 million years ago (Brochmann and Brysting 2008; Miller et al. 2010). The dynamic glaciation history of the Pleistocene caused large vegetation shifts, as well as speciation and diversification of shrubs (Brochmann and Brysting 2008; Kadereit and Abbott 2021). Arctic tundra shrubs have responded to climate change over the past 3 million years through changes in their distribution and abundance, as evidenced in the palaeoecological record (Birks 2008).

The impacts of Pleistocene glacial cycles on Arctic plants are often generalised as ice sheet expansion causing species' ranges to retract to climatically suitable land areas (refugia), leading to isolation in smaller populations and intraspecific divergence (Bennett and Provan 2008; Hultén 1937). This theory of population contraction during glacials is supported by higher genetic distinctiveness in former refugial regions (Eidesen et al. 2013). Fossil, molecular and phytogeographical evidence shows that the Beringian region (stretching from the Verkhoyansk mountains, east of the river Lena in Eastern Russia to the Mackenzie River in North America) has been an important refugium for shrubs such as dwarf birch species (Bigelow et al. 2003; Brubaker et al. 2005; Eidesen et al. 2015) and Arctic plants in general (Abbott and Brochmann 2003; Hewitt 2004; Hultén 1937). Conversely, ice retreat in interglacials caused population expansion from refugia into newly ice‐free areas as climatic and environmental conditions became suitable, leading to increased connectivity and gene flow among divergent populations (Hewitt 2004; Petit et al. 2003). The proposition that ice sheet retreat enabled recolonisation of plants is supported by a decline in plant species richness and genetic diversity with distance from glacial refugia (reflecting population expansion), except in regions of likely secondary contact between previously‐isolated populations (Pellissier et al. 2016; Stewart et al. 2016). The phylogeographic structure of several shrub species suggests repeated differentiation into intraspecific lineages and intraspecific hybridisation, likely in response to intermittent isolation and secondary contact in the Quaternary (Alsos et al. 2005; Ehrich et al. 2008; Eidesen, Alsos, et al. 2007; Eidesen, Carlsen, et al. 2007; Skrede et al. 2006).

Despite the support for this dichotomous narrative, the phylogeographic history of tundra shrub responses to glacial–interglacial cycles is likely more complex than the interplay between isolation in glacial refugia and enhanced connectivity in interglacials (Bennett and Provan 2008; Stewart and Dalén 2008). The contemporary geographical distribution and associated genetic structure of tundra shrubs have likely been influenced by a complex interplay of fluctuations in climate, ice sheets and associated changes in global sea level and land connectivity (Abbott and Brochmann 2003; Edwards et al. 2018; Elias and Brigham‐Grette 2013), as found in other species (Maresova et al. 2021).

Here we focus on *Betula nana* L. and *Betula glandulosa* Michx. (dwarf birches), two circumpolar and ecologically important shrubs of the Arctic tundra (de Groot et al. 1997). As deciduous shrubs, these species have been prominent in the multidecadal increases in aboveground biomass observed in the Arctic as a response to ongoing climate change (Mekonnen et al. 2021; Myers‐Smith et al. 2011; Sturm et al. 2001). The phylogeographic structure of *Betula nana* has previously been investigated (Eidesen et al. 2015; Palmé et al. 2004), but the response of dwarf birch species to past climate change is only partially understood (Crump et al. 2019, 2021). This is in part because the timescale of glacial cycles is finer than the temporal resolution that can be inferred from short phylogeographic genetic markers (Carstens and Knowles 2007), and thus much of the Quaternary history of dwarf birch species and associated paleoclimatic drivers remains unresolved.

We use a new range‐wide next‐generation sequencing genetic dataset together with coalescent modelling to link modern genetic structure with population demographic history in the Quaternary (Beichman et al. 2018; Kingman 1982; Wakeley and Hey 1997). This method has been used to infer postglacial range shifts and historical population fragmentation of *B. nana* in Britain (Borrell et al. 2018) and the Quaternary histories of organisms including Arctic plants (Ikeda et al. 2017) and tree birches (Chen and Lou 2019; Salojärvi et al. 2017; Tsuda et al. 2015, 2017). Using estimated divergence and secondary contact timings, we assessed the role of Quaternary climate change on the population history of dwarf birch species in the pan‐Arctic by comparing modelled population events to reconstructed ice sheet and sea level changes. We disentangle the effects of ice sheet changes, suitable climatic conditions and geography on the isolation and mixing of dwarf birches during the Quaternary and the subsequent impact on modern genetic structure.

## 2 Materials and Methods

### 2.1 Species

This study examines the genetic diversity of two dwarf birch species: *Betula nana* L. and *Betula glandulosa* Michx. (Figure S1). *Betula nana* (diploid with 2n = 28) is a shrub native to Arctic and boreal regions with a circumpolar distribution. It is monoecious, wind‐pollinated, wind‐dispersed and has a prostrate or upright growth habit up to 1 m in height (de Groot et al. 1997; Jadwiszczak et al. 2012). This species is sometimes divided into two subspecies: *B. nana* subsp. *nana* (Sukaczev) Hult. and *B. nana* subsp. *exilis* (Sukaczev) Hult. (Elven et al. 2011). The Svalbard population belongs to a western Siberian group identified in previous genetic analyses and is morphologically more similar to individuals in northwestern Russia than to Fennoscandia (Alsos et al. 2007; Elven et al. 2011; Eidesen et al. 2015). This group has been described as the subspecies *Betula nana var. tundrarum* Perfil. (Elven et al. 2011). *Betula glandulosa* (diploid with 2n = 28) is closely related to *B. nana* (Wang et al. 2016) and occurs in the Nearctic (de Groot et al. 1997). *B. glandulosa* has similar habitat preferences to *B. nana* but is less cold tolerant (de Groot et al. 1997). The species grows as tall, upright shrubs (up to 3 m in height) in the core of its range, but *B. glandulosa* and *B. nana* both become lower and more prostrate with smaller leaves in harsher environmental conditions and at their northern range limits (de Groot et al. 1997). Extensive historical and contemporary hybridisation and introgression within the *Betula* genus have resulted in high morphological variability within and among species (Palmé et al. 2004; Thomson et al. 2015; Thórsson et al. 2010; Tsuda et al. 2017), making taxonomic and phylogenetic inferences difficult (Järvinen et al. 2004; Li et al. 2005, 2007). There is disagreement on whether *B. nana* subsp. *exilis* and *B. glandulosa* should be considered distinct species, as suggested by genetic evidence (Eidesen et al. 2015; Touchette et al. 2024), or combined as *B. glandulosa* based on potential hybridisation and the morphological continuum observed in their area of range overlap (R. J. Abbott and Brochmann 2003; Ashburner and McAllister 2013; de Groot et al. 1997; Elven et al. 2011; Furlow 1997; Furlow 2004). *Betula glandulosa* hybridises with multiple North American shrub birch species, potentially forming part of a network of distinct species interconnected through hybridisation, termed a syngameon (Buck and Flores‐Rentería 2022; Touchette et al. 2024). *Betula glandulosa* also hybridises with *B. nana* subsp. *nana* in southern Greenland (Furlow 2004).

### 2.2 Sampling and DNA Extraction

Dwarf birch samples ( *B. nana* and *B. glandulosa* ) were collected during the growing season between 2015 and 2021, and were supplemented with samples collected between 2002 and 2004 that were previously sequenced (Eidesen et al. 2015), obtained with permission from the NHMO DNA Bank of the Natural History Museum, University of Oslo (see Appendix S1 for sampling protocol and Table S1 for collection metadata).

We developed a new DNA extraction protocol to overcome the difficulties of high‐quality DNA extraction resulting from the high concentration of polysaccharides and phenolics/polyphenols in dwarf birch leaves (Wang et al. 2013). We extracted total genomic DNA from dried leaves following this protocol (see Appendix S11). Sample quality was assessed using 1% gel electrophoresis, and DNA purity and concentration were assessed using a Qubit with dsDNA BR assay kits Fluorometer (Life Technologies Corporation, Carisbad, CA). Approximately 26% of samples were rejected due to degraded DNA, mostly due to senesced or limited starting material. Samples from six of the seven NHMO populations passed quality control. In all, 250 samples from 49 sites passed quality control. A subset of samples with sufficient DNA was duplicated (between 2 and 3 duplicates per sample) to increase sequencing coverage (Table S2), resulting in 376 DNA extracts in total.

### 2.3 RAD Sequencing and Bioinformatics

DNA extracts were submitted to FLORAGENEX (Beaverton, Oregon) for RAD (Restriction site‐associated) library preparation and sequencing (Baird et al. 2008; Davey and Blaxter 2010). After library quality assurance/quality control (QA/QC), libraries were pooled and fragments were sequenced across four lanes of an Illumina NovaSeq 6000 platform with single‐end 1 × 100 bp chemistry, which can deliver high sequencing depth and increase the confidence of base calls. Raw libraries were checked for errors using fastqc (Andrews 2010). Libraries were demultiplexed, and barcode sequences checked for errors using the process_radtags module of Stacks v2.60 (Catchen et al. 2011, 2013; Rochette et al. 2019). Reads from duplicated samples from the same original individual were combined. The sequencing depth coverage for each individual and population was assessed; six individuals with an unusual number of reads were removed from the analysis (Table S3). Reads were aligned to a reference *B. nana* genome (Wang et al. 2013, ENA accession number GCA_000327005) using Bowtie2 v2.5 with sensitive and end‐to‐end parameters (Langmead and Salzberg 2012). See Appendix S2 for further information on RAD sequencing and data processing.

Reference‐based Single Nucleotide Polymorphism (SNP) calling was performed using the gstacks module of Stacks. Preliminary analyses indicated that four sampling sites were hybrids with unknown birch species (Figure S2): these were excluded from further analyses, and genotype calling and filtering steps were repeated without them (see Appendix S3). The Populations module of Stacks was used to filter loci (see Appendix S4). SNPs were output as (a) files with phased haplotypes with multiple SNPs per locus and (b) as files retaining only the first SNP per locus. Gene diversity was extracted from the former, while rarefied allelic richness (corrected for sample size) was calculated from the latter using the hierfstat package v0.5–7 in R v4.0.3 (R Development Core Team, 2022).

### 2.4 Population Genetic Structure

We examined genetic variation among individuals using the single unlinked SNP dataset and Nonmetric Multidimensional Scaling (NMDS), a rank‐based multivariate ordination technique that addresses some of the limitations of analysing SNP data with Principal Component Analysis (PCA) (Miclaus et al. 2009; Minchin 1987; Novembre and Stephens 2008). NMDS was implemented on a distance matrix of pairwise allelic differences (Hamming Distance (Wang et al. 2015)) using the diss.dist function from the R package poppr v2.9.6 (Kamvar et al. 2024) and the metaMDS function from the vegan package v2.6–6.1 (Oksanen et al. 2022), with 95% confidence ellipses calculated using the stat_ellipse function in ggplot2 v3.5.1 (Wickham et al. 2024) under default options. Unlike the commonly used Euclidean distance (Georges et al. 2023), diss.dist () can handle multi‐allelic loci and missing SNPs, and also captured a wider range of variation in distances in our data. All analyses in R were conducted using R v4.4.1 (R Development Core Team 2024) and RStudio v2024.04.1 (Posit team 2024).

After ordination, we used the software ADMIXTURE v1.3.0 (Alexander et al. 2009; Alexander and Lange 2011) to infer the number of ancestral populations and to identify potential historical admixture events based on the proportion of an individual's alleles that come from K hypothesised ancestral populations (Alexander et al. 2009). We conducted ADMIXTURE analyses on the global dataset and for each regional subset using default parameters. We tested a range of 2–7 possible ancestral populations (K); selecting the K with the lowest cross‐evaluation error (Oreskes 1998). We plotted individual population assignment probabilities using the R package ggplot2 v3.5.1 (Wickham et al. 2024).

To investigate potential gene flow between the ancestral populations identified by ADMIXTURE, D statistics and F4 ratios were calculated using the admixr v0.9.1 (Petr 2020) interface of ADMIXTOOLS v7.0.2 (Patterson et al. 2012) which was installed via Bioconda (accessed 24 June 2025). ADMIXTOOLS has stringent filters to keep only SNPs which are informative for detecting admixture. Therefore, these statistics were calculated using an alternative SNP dataset with relaxed filtering parameters, no LD filtering or MAF threshold filtering and keeping multiple SNPs per locus to retain enough SNPs for D statistic calculation, resulting in 500 SNPs. Quartet topology was (nana, exilis, tundrarum, glandulosa east) with the west European and Greenland Nana samples separated for the F4 ratio test in a sister group to the main Nana group. We used block jackknife resampling and retained 2325 polymorphic SNPs, of which 500 were informative for these tests.

To examine the relationship between population structure (Meirmans 2012) and isolation by distance (IBD, a correlation between genetic and geographic distance), we assessed individual‐based IBD both globally and within the genetic clusters that were defined using ADMIXTURE, using Mantel tests with 999 permutations, implemented in the dartR 2.9.7 package (Gruber et al. 2023). A Haversine great circle distance matrix was created using the geosphere package v1.5–18 (Hijmans et al. 2022). A partial Mantel test using the vegan package v2.6–6.1 evaluated the association between genetic distance and genetic clusters with geographic distances as a covariate, that is, controlling for IBD (Drummond and Hamilton 2007).

We examined global and regional population structure simultaneously using the linkage structure of haplotypes with fineRADstructure v0.3.2 (Malinsky et al. 2018). A coancestry matrix summarising nearest neighbour haplotype relationships was calculated using default parameters. The coancestry matrix was then ordered according to a tree‐like structure of population relationships produced by a Markov Chain Monte Carlo (MCMC) clustering algorithm, which assigns individuals to populations and then builds a tree based on the best state. Ordering the individuals according to the clustering tree helped visualise whether individuals are more closely related to individuals from other populations than to individuals within their own population, indicative of inter‐population gene flow and admixture.

### 2.5 Developing and Testing Demographic Modelling Scenarios

The aim of our demographic modelling was to infer the timing of splits and the timing and degree of gene flow among populations, to understand when the modern genetic structure of dwarf birch species arose. We used approximate Bayesian computation (ABC) to test evolutionary scenarios (Beaumont 2010; Bertorelle et al. 2010; Cornuet et al. 2014), specifically the software DIYABC Random Forest v1.0 in combination with supervised machine learning based on Random Forests (RF) (Collin et al. 2021) for data simulation, model classification (model selection) and parameter estimation.

We used a hierarchical model testing approach where simpler models were tested and improved using a subset of the data before the best scenarios were combined into a final pan‐Arctic model set (Chen and Lou 2019). These were as follows:

1. Global models, exploring the divergences of the three main genetic groups excluding individuals identified as admixed.
2. Regional models for Eurasia and America, including intraspecific divergences and gene flow events.
3. Global models combining scenarios from the regional models, with additional population events between regions.

Populations in DIYABC models were defined based on ancestral populations inferred from ADMIXTURE results (see Appendix S5). Scenarios were developed based on the population genetic structuring analyses and were also informed by glacial and postglacial histories of *B. nana* inferred from a previous phylogeographical analysis (Eidesen et al. 2015). For each scenario, the following parameters were specified: (i) populations, (ii) topology (i.e., the branching structure of the phylogenetic tree), (iii) prior time ranges for events and (iv) prior ranges for effective population sizes (Ne) and bottleneck magnitudes. Secondary contact events between populations were coded as discrete periods of unidirectional gene flow from one population into another established population (introgression) or bidirectional gene flow between populations, resulting in a new, admixed population (admixture). Minor allele frequency (MAF) was set to 0.025. Regional models were combined into global models once the most likely regional topologies had been established. This allowed the Eurasian and North American Exilis populations to be combined in one population, which was supported by their genetic similarity in the NMDS and the intermittent continental connectivity during the Quaternary (Kender et al. 2018). See Appendix S6 for details on model sets and scenarios, including Table S5 for model specification of the final global model used for parameter estimation.

For each set of models, we generated 10,000 simulated genetic datasets, drawing parameter values from the prior distributions. The number of trees for model selection and parameter estimation was fixed at 500 to ensure stable estimation of the global error rate. Otherwise, default software parameters were used. The best‐performing scenarios were selected based on model error rates and on visual assessment of overlap between simulated and observed data when projected on linear discriminant analysis (LDA) axes. LDA also helped to refine prior ranges for new iterations of the scenarios. The best‐performing scenarios were iteratively improved and tested until a final set of models was produced in which the local model error rate was reduced to approximately 20% or less (see Table 1 Collin et al. 2021), complete overlap occurred between simulated and observed data, and posterior probabilities were above 65% (Collin et al. 2021). See Appendix S6 for LDA projections, including Table S6 for model error rates and Table S7 for posterior probabilities of all model sets.

### 2.6 Population History Parameter Estimation

The most plausible evolutionary scenario with the highest posterior probability was selected from the final global model set, and parameters were estimated using independent RF treatments. The simulated training sets included ~25,000 datasets per scenario for parameter estimation (100,000 datasets/four scenarios). Point estimates and 95% credible intervals were calculated along with global and local accuracy metrics. Estimates were converted from number of generations to calendar years, assuming a generation time of between 10 and 14 years. Estimates of generation time in dwarf birch are limited by a lack of evidence on average age at first recruitment, long lifespans, vegetative production and climate‐dependent recruitment (Büntgen et al. 2015; Tsuda et al. 2017). Our generation time was estimated using a Scandinavian‐British *B. nana* divergence from RAD‐seq coalescent modelling (Borrell et al. 2018), calibrated using first occurrences of Betula pollen in Scotland, with two generation times tested to account for uncertainty in the pollen record (see Appendix S7 for details).

SNPs do not require mutation model parameterisation (Collin et al. 2021), but this means that population event timings are not calibrated by mutation parameters. Ratios of time divided by effective population size can therefore be estimated, although point estimates and confidence intervals are more difficult to interpret biologically. Consequently, raw parameters are often used (Bernos et al. 2023; Huanel et al. 2022; Kozhar et al. 2022; McDevitt et al. 2022; Pless et al. 2022; Senczuk et al. 2021; Strumia et al. 2021). We estimated both types of parameters and found local posterior RMSE estimates to be lower in raw parameters. Hence, results from raw parameters are presented.

### 2.7 Comparison With Paleoclimate and Paleoenvironmental Data

Median estimates of the timing of key demographic events from the best performing model were compared to global and regional Pleistocene ice sheet reconstructions to infer spatiotemporal correspondence with potential climatic drivers (Batchelor et al. 2019; Dalton et al. 2020, 2022; Hughes et al. 2016). Best estimates of ice sheet coverage at dated time intervals were derived from empirical (geochronological, stratigraphic, geomorphological) and numerical data (modelling output). The global reconstruction consisted of 17 time slices spanning the pre‐Quaternary, ~3.2 million years ago (mya), to the Last Glacial Maximum (LGM), with ice sheet coverage in the early Quaternary and in regions such as northeast Asia less well constrained. Higher temporal resolution reconstructions were available from 115 to 25 thousand years ago (ka) (5 ka intervals) and 25–6 ka (0.1–0.7 ka intervals) for the Laurentide and Innuitian ice sheets in North America (Dalton et al. 2020, 2022), although reconstructions were more loosely constrained prior to 45 ka (Dalton et al. 2022). Reconstructions of the Eurasian Ice Sheet were available from 25 to 10 ka (1 ka intervals) and 40–25 ka (four intervals) (Hughes et al. 2016). We also obtained estimated dates of Bering Land Bridge emergence during the Quaternary based on geophysical and sediment records and geochemical proxies (Farmer et al. 2023; Jakobsson et al. 2017; Kender et al. 2018). We compared the Svalbard divergence date to dates of first occurrence of *B. nana* in Svalbard inferred from sedimentary DNA (sedaDNA) (Alsos, Sjögren, et al. 2016).

## 3 Results

We used a new RADseq SNP dataset (3.1) in concert with an approximate Bayesian computation coalescent modelling framework to link dwarf birch population genetic structure (3.2) with demographic history (3.3) during the Quaternary (~2.6 million years ago–present). We compared modelled population divergence and secondary contact times to reconstructed ice sheet and sea level changes, and other paleoenvironmental information (3.4), to assess the impacts of past climate changes on the population history of dwarf birch species.

### 3.1 RADseq SNP Discovery

**FIGURE 1:**
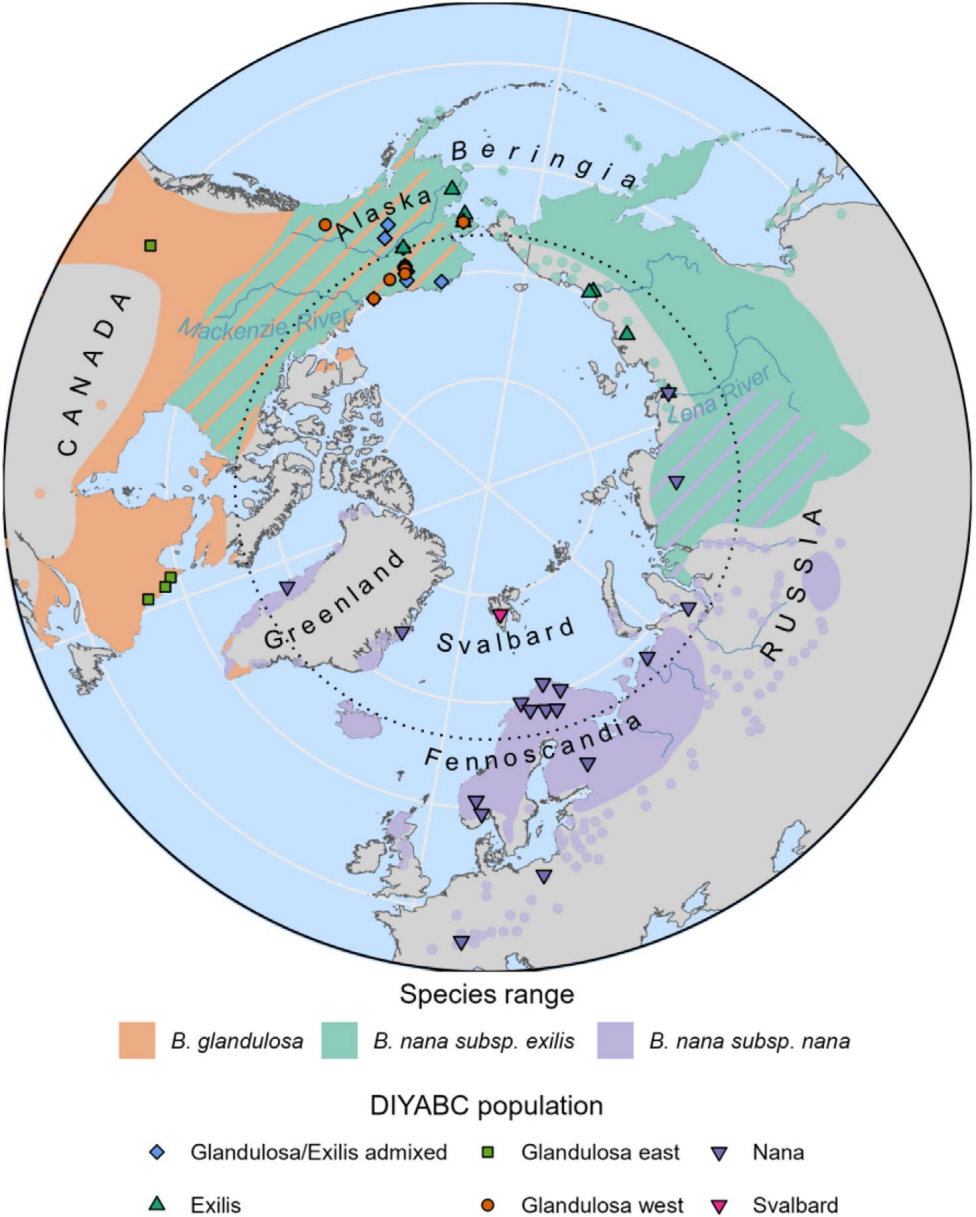
Dwarf birch species distributions and sampling sites. Map of the geographic range of the three study species along with their areas of range overlap (stripes) and sampling sites (*n* = 43) coloured according to genetic populations determined for approximate Bayesian computation (ABC) modelling with DIYABC RF (Collin et al. 2021). Two populations that were geographically close in the River Lena area were assigned to different populations: Exilis and Nana, and some individuals from the same sampling sites in Alaska were assigned to different populations. Populations were assigned using ancestral group coefficients from ADMIXTURE analysis: see Appendix S5 for details. Core species ranges are indicated with coloured shading, while more isolated occurrences are indicated with coloured points. *Betula nana* is found outside its core range in southerly populations in Europe that are glacial relicts (Eidesen et al. 2015; Hultén and Fries 1986; Jadwiszczak et al. 2012). *Betula glandulosa* 's range extends south through the mountain ranges of western North America, with isolated occurrences in mountain ranges of eastern North America (Furlow 2004). Eurasian ranges were adapted from Hultén and Fries (1986) and American ranges from Furlow (1997). See Figure S25 for a detailed map of Alaskan populations.

A total of 419,484,325 reads were generated from four single‐end Illumina Novaseq lanes, of which 91.33% passed quality controls. The number of reads per individual ranged from 351,332 to 3,596,943, with a mean of 1,126,061. Across all individuals, 87.7% of reads successfully mapped to the *B. nana* reference genome, with 71.7% retained with a mapping quality > 10 (gstacks default). After removing individuals with anomalous coverage and the hybrids with unknown species (see Appendix S3), we genotyped 218 individuals from 43 sampling sites (Figure 1), resulting in 2180 loci with varying spatial coverage in the pan‐Arctic dataset. Mean effective per‐sample coverage after gstacks was 346 × (min 99.7, max 1031). Haplotype phasing was successful for 74.5% of loci, resulting in 7528 variant sites, including 3830 unlinked sites. The single SNP dataset contained 1744 variant sites of which 1041 were unlinked.

### 3.2 Global and Regional Population Genetic Structure

**FIGURE 2:**
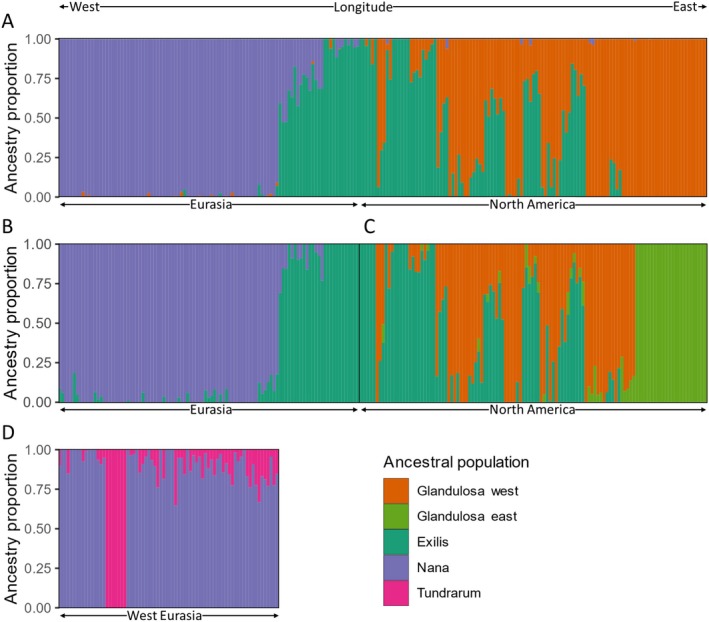
Dwarf birch ancestry proportions inferred from hierarchical ADMIXTURE analyses (Alexander et al. 2009; Alexander and Lange 2011). Ancestry proportions are displayed for each individual and arranged by longitude. Colours represent the optimal number (lowest cross evaluation error) of proposed ancestral populations (*K*). Separate analyses were run for (A) the global dataset with 1041 SNPS (*K* = 3), (B) Eurasia with 1072 SNPS (*K* = 2), (C) America with 864 SNPS (*K* = 3), and (D) western Eurasia with 1040 SNPS (*K* = 2). Individuals are arranged by longitude, from Greenland on the left to eastern Canada on the right.

**FIGURE 3:**
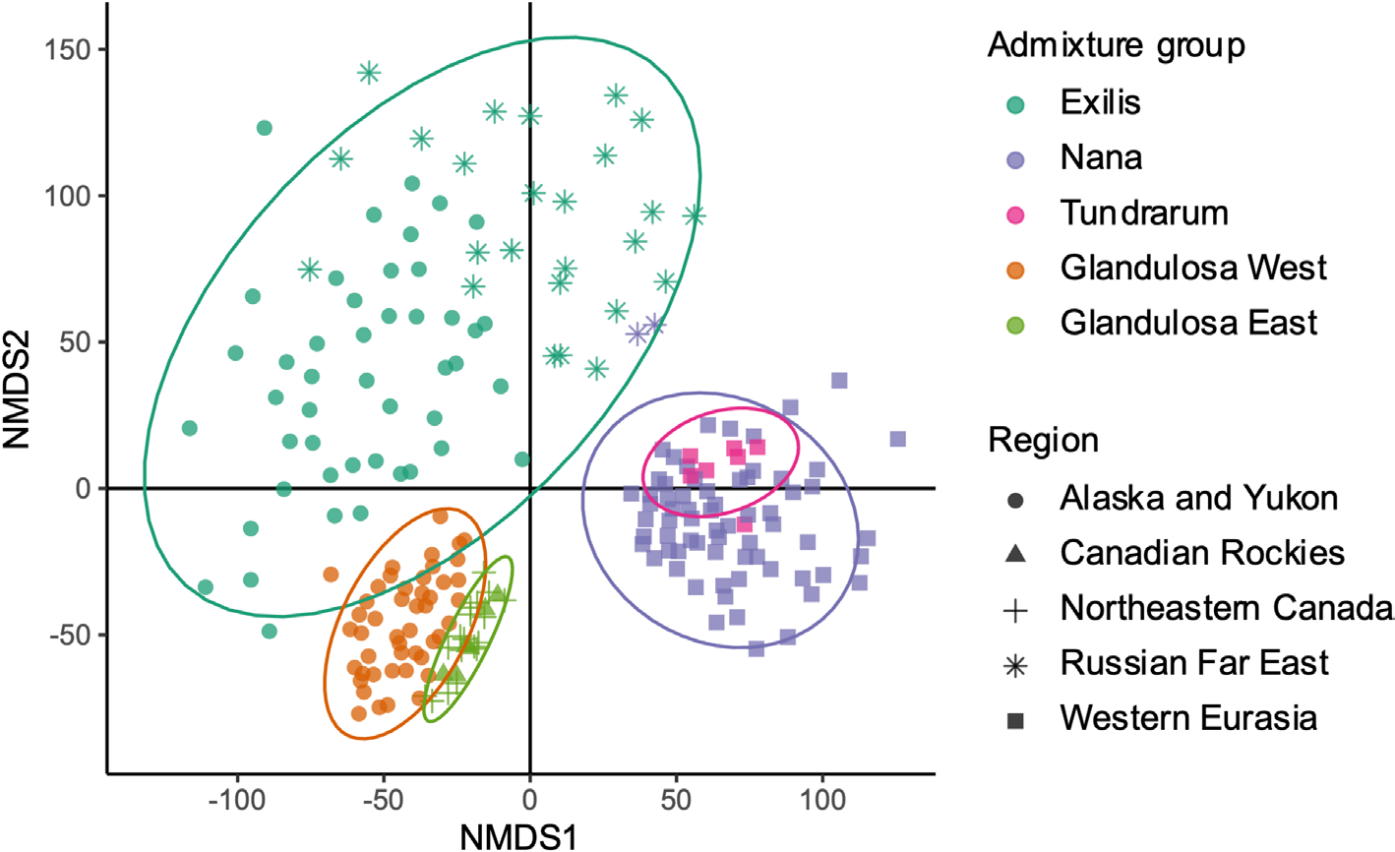
Ordination plot summarising genetic variation in SNPs among dwarf birch individuals (*n* = 218). Nonmetric multidimensional scaling (NMDS) with colours corresponding to ADMIXTURE ancestral groups and shapes corresponding to sampling region. Individuals that cluster more closely together are more similar based on relative allelic differences. Ellipses represent the 0.95 confidence level within which the true group centroids lie, assuming a t distribution. The stress value of the NMDS was < 2 at 0.165, suggesting a good fit: the reduced‐dimensional representation preserves the original distances between samples.

In the following sections, taxonomic (sub‐)species are referred to in italics, while genetic groups obtained from population structure analyses, and DIYABC populations are in regular text and capitalised. Global ADMIXTURE and RADPAINTER analyses identified three major ancestral populations, corresponding to *B. glandulosa* in North America and two *B. nana* populations. One *B. nana* ancestral population occurred in western Eurasia and another in eastern Eurasia and Alaska (Figures 2A, 3, S27). This is congruent with previous phylogeographical and taxonomic work identifying eastern and western *B. nana* groups, corresponding to two often recognised subspecies: *B. nana* ssp. *nana* (Suk.) Hult. and *B. nana ssp. exilis* (Suk.) Hult. (Eidesen et al. 2015; Elven et al. 2011; Hultén and Fries 1986). Hereafter, the three ancestral populations are referred to as ‘Glandulosa’, ‘Nana’ and ‘Exilis’.

The NMDS ordination showed that Nana and Glandulosa were genetically distinct (Figure 3), and that Nana and Exilis were also genetically distinct except for two Nana individuals that clustered with Exilis. However, apparent gene flow between Nana and Exilis was visible in the ADMIXTURE plot as a cline of admixture from the Lena River eastwards to the Kolyma River (Figure 2A). The NMDS also showed that the largest genetic variability was within the Exilis group, and that some Alaskan Exilis individuals were genetically more similar to Alaskan Glandulosa than to Exilis individuals in the Russian Far East (Figure 3). Many individuals in Alaskan populations were admixed between Exilis and Glandulosa (Figure 2A) and the RADPAINTER analysis showed that they shared closer co‐ancestry with individuals from other populations (Figure S28). See Table S8 for population‐level genetic diversity.

Finer‐scale population structure was evident in the regional ADMIXTURE and RADPAINTER results. The regional North American results suggested two ancestral Glandulosa populations: a western Glandulosa group in present‐day Alaska and the Yukon, and an eastern Glandulosa group consisting of two distinct populations in the Canadian Rockies and Labrador (Figure 2C, Figure S29). Populations sampled from Yellowknife, NWT (excluded from further analyses due to potential hybridisation with another *Betula* species, see Appendix S3), also appeared to contain eastern Glandulosa ancestry. A small proportion of ancestry from eastern Glandulosa was also present in eastern Alaskan populations that were predominantly western Glandulosa (Figure 3C).

The Eurasian ADMIXTURE analysis suggested differing patterns for Nana‐Exilis admixture depending on whether Exilis individuals were included or not (Figure 2B and Figure 2D). Admixed populations occurred in the Taimyr Peninsula east of the Lena River, while admixture extended eastwards to the Yana‐Indigirka Lowlands. This transition corresponds to a previously inferred area of range overlap (Hultén and Fries 1986). However, ADMIXTURE analysis of western Eurasian populations suggested that apparent Exilis ancestry in western Eurasia was due to admixture with another ancestral population (Figure 2D). This population was named Tundrarum, as it corresponded geographically with the subspecies *Betula nana var. tundrarum* Perfil. (Elven et al. 2011). The proportion of Tundrarum ancestry increased from northeast Fennoscandia to western Siberia and dominated the Svalbard population (Figure 2D). Central European refugial populations in the Alps and Poland formed distinct clusters (Figure S30), and southern Norway and Greenland clustered together, similar to a distinct Atlantic *B. nana* subgroup identified with AFLP data (Eidesen et al. 2015).

To test our hypothesis of an admixed origin for Tundrarum from Exilis and Nana, we calculated D‐statistics with Glandulosa east as an outgroup. We found a significant excess of derived allele sharing between Tundrarum and Exilis (D ≈0.63, |Z| ≈7), indicating either a sister‐group relationship or post‐divergence gene flow from exilis into tundrarum. The Svalbard population has been subject to bottlenecks in the Holocene that could also lead to an apparent admixture signal if surviving derived alleles were biassed towards Exilis. F4 ratios that estimate the mixing proportions of an admixture event were estimated to distinguish between these scenarios and indicated that ~99% ±16% of the Svalbard Tundrarum genome derives from Exilis (*α* = 0.99, SE = 0.16, *Z* = 6.3). However, non‐metric MDS using genome‐wide distances clustered the Svalbard Tundrarum samples with Nana, which together suggest a hypothesis of admixture between Nana and Exilis forming the Tundrarum population, with most of the material from Exilis.

Mantel tests (Figures S25, S26) identified significant isolation by distance (IBD) across the Arctic (*r* = 0.501, *p* = 0.001) although within lineage effects were small (Exilis *r* = 0.25, *p* = 0.001, Nana *r* = 0.209, *p* = 0.004) genetic group. Geographic distance thus explains a minor fraction of genetic variation within these widespread groups, suggesting that other factors are more influential at that scale. Since *B. nana* expanded across Eurasia over large distances during postglacial dispersal, disruption of the classic IBD pattern due to serial founder events, long distance dispersal or secondary contact with divergent lineages may have occurred. Glandulosa east showed strong IBD (*r* = 0.627, *p* = 0.001), potentially inflated by incomplete geographical range sampling, while Glandulosa west displayed no significant IBD (*r* = 0.064, *p* = 0.279). After controlling for geographic distance, a partial Mantel test indicated significant genetic structure among ADMIXTURE groups (*r* = 0.356, *p* = 0.001), indicating that the observed clusters were not an artefact of IBD (Meirmans 2012).

### 3.3 Demographic History

**FIGURE 4:**
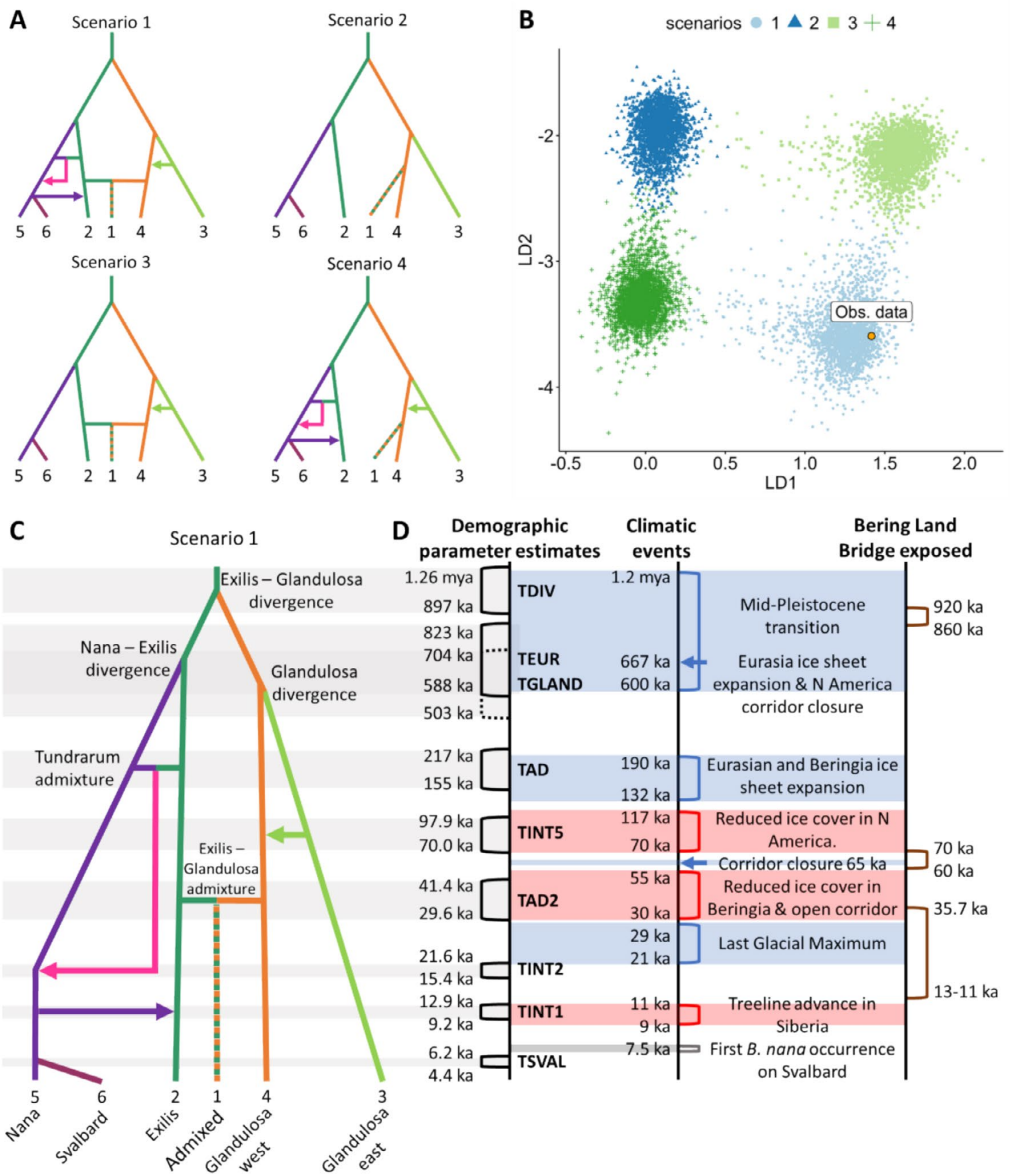
Quaternary demographic models of dwarf birch plotted against climatic and environmental changes. (A) Topologies of the four scenarios in the final global model set. Lineage colours correspond to the DIYABC populations from left to right: Nana, Svalbard, Exilis, Admixed, Glandulosa west and Glandulosa east, as in Figure 2. Scenario one contained interglacial, glacial and postglacial secondary contact and gene flow. Scenario two contained no secondary contact or gene flow. Scenario three contained interglacial gene flow in North America only, and scenario four contained secondary contact and gene flow without Exilis‐Glandulosa admixture. (B) Projection of simulated datasets of the scenarios on the first two axes of a Linear Discriminant Analysis. A random subsample of 10,000 simulated datasets out of the 100,000 used for model choice are plotted. The location of the observed genetic data is indicated by the orange circle. (C) Topology depicting the best supported scenario according to DIYABC RF model choice with a posterior probability of 0.891. Divergence events and bidirectional gene flow (admixture) events are labelled, while the arrows indicate unidirectional gene flow between populations (introgression). (D) The timing of population events from the best supported scenario compared with climatic and environmental changes in the Quaternary inferred from the literature. Population events given as a range between the median estimates calculated using different generation times (10–14 years); see Table S9 for 95% confidence intervals. Time is not to scale.

The final set of global models contained six populations: Exilis, Nana, Svalbard, Glandulosa west, Glandulosa east, an admixed Glandulosa west/Exilis population, and one unsampled Tundrarum population (Figure 4). In the DIYABC RF analysis, scenario 1 had the highest probability (Figure 4) and was characterised by an ancestral Exilis population and admixture and introgression events in Eurasia and North America. Alternative models with Glandulosa as the ancestral population had lower posterior probability (Table S7), and a model with Nana as the ancestral population did not have simulated data in close agreement with the observed data (Figures S20, S21). Estimates of recent population events were more accurately constrained than population events occurring earlier in time (Table S9, Figure S31). Parameter estimates are summarised in Figure 4.

### 3.4 Comparing Demographic History and Quaternary Paleoenvironmental Changes

The main species and intraspecies dwarf birch divergences occurred during the intensification of glacial conditions during the Mid‐Pleistocene Transition (1.2 mya–600 ka), according to our best supported model (Figure 4). The divergence of Nana and Exilis lineages, presumably in Eurasia given their contemporary distributions, encompassed the period ~667 ka (MIS16) when a major expansion of northwest Eurasian ice sheets occurred; ice sheets also expanded in the uplands of west Beringia, in present‐day Russia (Batchelor et al. 2019). The divergence of the two Glandulosa lineages, likely in North America, encompassed a similar time period, when the closure of an ice‐free corridor between the Laurentide (eastern) and Cordilleran (western) ice sheets occurred. This corridor had connected Alaska with the rest of North America, which then remained closed for ~330 ka.

Our model supported that secondary contact and admixture between Exilis and Nana occurred during the maximum Pleistocene extent of ice sheets in northwest Eurasia ~190–132 ka (MIS 6), with extensive glaciation in western Beringian uplands (Batchelor et al. 2019). In contrast, secondary contract and introgression from Glandulosa east to Glandulosa west was estimated to have occurred during a period of much reduced ice cover in North America with potential connectivity along the ice‐free corridor between continental America and Beringia ~117 ka–60 ka (MIS 4) (Batchelor et al. 2019; Dalton et al. 2022).

Admixture between Glandulosa west and Exilis was estimated to have occurred during another reopening of the ice‐free corridor between the Cordilleran and Laurentide ice sheets and ice sheet retreat from the Yukon and Brooks Range ~55–30 ka (Batchelor et al. 2019; Dalton et al. 2022). During this period (MIS 3) conditions in Beringia were similar to the present, with temperatures 1°C–2°C lower and spruce forest present in interior Alaska and the Yukon (Miller et al. 2010).

The initial retreat of the Eurasian Ice Sheet complex after the LGM occurred ~23–21 ka in Eurasia (Hughes et al. 2016), with our model suggesting postglacial secondary contact among the Nana, Tundrarum, and Eurasian Exilis lineages following this. Maps of inferred population divergence and gene flow events with corresponding ice sheet coverage at each time step are presented in Figure S34.

## 4 Discussion

Multiple population divergences, secondary contact and gene flow events in the best‐supported coalescent model coincide temporally with climatic and ice sheet changes (Figure S34). This correspondence suggests that glacial cycles may have influenced dwarf birch population dynamics. However, the response of dwarf birch species to past climate change appears to be more complex than a simple glacial isolation‐interglacial expansion population history, with climate conditions in ice‐free areas potentially influencing distribution and associated population connectivity during both glacial and interglacial intervals.

### 4.1 The Mid‐Pleistocene Origin of the Dwarf Birch Species Complex

The taxonomy and evolutionary relationships of dwarf birch species have long been unclear (Ashburner and McAllister 2013; Elven et al. 2011; Hultén and Fries 1986). Our best demographic model supported a divergence between *B. nana* (specifically, the Exilis genetic group) and *B. glandulosa* (specifically, the Glandulosa west genetic group) in the Mid‐Pleistocene between 1.26 mya (CI 1.13–1.60 mya) and 897 ka (CI 806 ka–1.15 mya). Macrofossil and pollen records with *B. nana* ‐type morphology in North Greenland and northeast Russia from ~3–2.6 mya show a dwarf birch taxon with a widespread Arctic distribution before the onset of Pleistocene glaciations, and a marked increase in pollen abundance with the expansion of the tundra biome at ~2.7 mya (Andreev et al. 2014; Bennike and Böcher 1990). Thus, the onset of glaciations provided new opportunities for dwarf birch expansion, although glacial cycle intensification in the Mid‐Pleistocene Transition (1.2 mya–600 ka) likely isolated dwarf birch in two lineages west and south of North American ice sheets, leading to allopatric speciation between *B. nana* and *B. glandulosa*, respectively.

Regardless, Beringia was the likely source for Pleistocene diversification, as the amphiberingian Exilis was ancestral in the best supported model. A Beringean origin of the current dwarf birches was previously suggested based on a shared ancestral pDNA haplotype between *B. nana*, *B. glandulosa* and *B. pubescens* that is dominant in Beringia (Eidesen et al. 2015). Previous phylogeographic work has also indicated that Beringia was an important source of early Pleistocene dispersals and colonisations for plants expanding into the Arctic (R. J. Abbott et al. 2000; R. J. Abbott and Comes 2004; Alsos et al. 2005; Eidesen 2007; Hultén 1937). The genetic similarities we identified between the Russian Far East and southwest Alaska further support the importance of the Beringian region as a glacial refugium for dwarf birch (Eidesen et al. 2015). Sub‐refugia within Beringia may have enabled Exilis and Glandulosa to remain distinct despite occasional gene exchanges, as suggested for other Arctic plant species where divergent genetic groups co‐occur within Alaska (R. J. Abbott and Comes 2004; Jorgensen et al. 2003).

Intraspecific divergences within *B. nana* and *B. glandulosa* are also estimated to have occurred during the Mid‐Pleistocene. Within *B. nana* , the Nana and Exilis groups diverged from 823 ka (CI 389 ka–1.31 mya) to 588 ka (CI 278–937 ka). They may have diverged due to the westward expansion of Nana into ice‐free Eurasian areas, followed by separation due to expanding ice sheets ~677 ka, with increasingly unsuitable climate conditions in ice‐free areas maintaining separation (Batchelor et al. 2019). Similarly, closure of the corridor between the Cordilleran and Laurentide ice sheets ~677 ka may have isolated *B. glandulosa* in two separate areas until at least 429 ka (Batchelor et al. 2019), leading to the two distinct genetic groups we identified, with an estimated divergence time between 704 ka (CI 221 ka–1.20 mya) and 503 ka (CI 158–857 ka). A recent genetic analysis also identified distinct eastern and western genetic clusters within *B. glandulosa* , suggestive of two glacial lineages with different refugia (Touchette et al. 2024). Continental dwarf birch divergences in the Quaternary therefore appear to be linked to isolation following climate deterioration.

### 4.2 The Complex Role of Ice Sheets in the Quaternary

The latter part of the Quaternary history of dwarf birch species was characterised by secondary contact of divergent lineages, with bidirectional gene flow resulting in new admixed populations, or unidirectional gene flow from one population into another established population (introgression). Unlike population isolation and divergence events, these events appear to coincide with both periods of ice sheet retreat and expansion.

Introgression from Glandulosa east to Glandulosa west—estimated between 97.9 ka (39.5–212 ka) and 70.0 ka (CI 28.2–151.5 ka)—occurred during a period of much reduced ice cover in North America, suggesting a north‐ and northwestwards range shift of the eastern Glandulosa group, resulting in secondary contact between the two Glandulosa groups. Similarly, admixture between Exilis and the western Glandulosa groups—estimated between 41.4 ka (CI 9.06–91.2 ka) and 29.6 ka (CI 6.47–56.2 ka)—coincided with ice sheet retreat within Beringia prior to the LGM (Batchelor et al. 2019). The re‐emergence of the Bering Land Bridge from 35.7 ka onwards (Farmer et al. 2023) very likely increased population connectivity. Since modern Exilis populations in southwest Alaska were genetically closer to individuals in the Russian Far East than to other Exilis populations in Alaska, intermittent connectivity over land or across sea appears to have been sufficient to maintain gene flow across Beringia during the Quaternary.

The Exilis–Nana admixture event between 216 ka (CI 136–323 ka) and 154 ka (CI 97–231 ka) resulted in an unsampled population, Tundrarum, that remained separate during the last glacial period. Isolation of *B. nana* south of the Eurasian ice sheet, and the existence of a distinct, admixed population located in a separate west Siberian refugium, the source of the present‐day west Siberian subspecies (*Betula nana var. tundrarum* Perfil.), was previously hypothesised based on AFLP markers (Alsos et al. 2007; Eidesen et al. 2015) and dwarf birch pollen in Lake Taymyr during the LGM (Andreev et al. 2002). The Exilis–Nana admixture event occurred during the maximum Pleistocene coverage of ice sheets over Siberia and the west Beringian uplands (Batchelor et al. 2019). Ice sheet expansion may have increased the likelihood of secondary contact between geographically separated populations, similar to caribou glacial introgression events during the last glacial cycle in North America (Taylor et al. 2021). Nana and Exilis populations may have come into contact by contracting towards the Lena River and Beringian coastal lowlands, where the modern population genetic structure suggests that postglacial admixture between Nana and Exilis also occurred. Alternatively, the two populations may have both expanded into areas that are currently taiga and so come into contact.

The history of *B. nana* lineages in the postglacial period follows a pattern of range expansion from refugia and secondary contact and gene flow during warmer periods. The isolation by distance (IBD) identified in the Nana and Exilis groups has been previously found in *B. nana* on a regional scale (Wang et al. 2014), and is indicative of postglacial range expansion from refugia (Sharbel et al. 2000; Wahlsteen et al. 2023). Gene flow from Tundrarum to Nana was estimated between 21.6 ka (CI 8.48–32.44 ka) and 15.4 ka (CI 6.06–23.2 ka), coinciding with the retreat of the Eurasian Ice Sheet complex after the LGM (which was ~21–23 ka in Eurasia) (Hughes et al. 2016). The presence of *B. nana* macrofossils in periglacial sites along the edges of the Eurasian ice sheet from 22.2 to 17.2 ka (Binney et al. 2009) suggests that Nana came into contact with Tundrarum early in the postglacial period as it closely tracked the retreating ice sheets. This contrasts with evidence from Baffin Island where dwarf birch shows a lagged migration response to suitable climates in the postglacial period (Crump et al. 2019), which may be due to distant potential source regions caused by remnants of the Laurentide ice sheet to the south of Baffin Island (Dalton et al. 2020), or delayed soil development (Matthews 1992).

Comparison of our modelled population events with ice sheet reconstructions suggests that dwarf birch lineages underwent secondary contact and admixture during periods of climatic transition, rather than during a particular absolute climate state. As dwarf birch has large high dispersal potential over long distances (Alsos et al. 2007; Alsos et al. 2015; de Groot et al. 1997), rapid colonisation of newly suitable areas may have promoted mixing during climate transitions (Meucci et al. 2021).

### 4.3 Dwarf Birch as a Thermophilous Arctic Shrub: Population Connectivity in Ice‐Free Areas

The varying effects of Pleistocene glacial cycles on the population dynamics of dwarf birch species show that the species do not fit the glacial refugial isolation and postglacial expansion model of temperate and boreal tree species, often assumed for a wide range of taxa (Hewitt 2004). They also do not fit the model of a ‘true Arctic’ species, such as *Dryas octopetala* (L.), which expanded its range in glacials and contracted its range in interglacials (Bennett and Provan 2008). Dwarf birch species instead behave as intermediate taxa, thermophilous from an Arctic (tundra) point of view, but cold‐adapted from a boreal/temperate perspective. Gene flow from *B. nana* into the tree species *B. pubescens* at the southern range limit for *B. nana* supports this intermediate status (Eidesen et al. 2015).

For relatively thermophilic Arctic taxa such as dwarf birch species (Alsos et al. 2002; Crump et al. 2019), less suitable abiotic conditions such as the cold and dry characteristic of glacials may have formed a barrier to population connectivity (Soberón and Nakamura 2009). Large parts of the north Eurasian continent were not glaciated during glacial periods (Batchelor et al. 2019) but consisted of a treeless tundra‐steppe ecosystem. Although the prevailing flora of this ecosystem is still debated (Clarke et al. 2020 and refs within), it was likely dominated by forbs and graminoids, since shrubs appear to have been a rare component of this vegetation in the paleorecord (Ager and Phillips 2008; Andreev et al. 2002; Bigelow et al. 2003; Binney et al. 2017, 2009; Müller et al. 2009). These unglaciated areas may have been too cold and dry to sustain large communities of shrubs that would enable connectivity between refugial areas in western Eurasia and Beringia (Anderson and Lozhkin 2015; Kienast et al. 2001, 2005).

Ice‐free areas in the Eurasian and North American high latitudes during warmer interglacial periods may have also been unsuitable for dwarf birch. For example, tree birches and conifers occurred far north of their contemporary limit across northern Eurasia from 15 to 5 ka (Binney et al. 2009); consequently, the reduced area of tundra habitat may have restricted the distribution of dwarf birch and led to population isolation (Stewart et al. 2010). The postglacial northward retreat of dwarf birch left behind small populations at more southerly latitudes (de Groot et al. 1997; Eidesen et al. 2015; Hultén and Fries 1986; Jadwiszczak et al. 2012; Wang et al. 2014). These glacial relict populations (Hampe and Jump 2011) persist in locally suitable conditions—cryptic refugia (Stewart et al. 2010), such as cooler mountainous areas or exposed terrain. Similarly, remnants of more northerly, early Holocene distributions in the form of clonal populations in Svalbard and Baffin Island—areas currently too cold for sexual reproduction and seed set—show that the species can be isolated by unsuitable climatic conditions (Alsos et al. 2002; Engelskjøn et al. 2003; Hermanutz et al. 1989).

Increased dwarf birch isolation in both the coldest and warmest periods during the Pleistocene agrees with increased connectivity during climate transitions and could also explain the maintenance of distinct Exilis and Glandulosa groups in Beringia.

### 4.4 Svalbard Colonisation Likely Obscured by Late Holocene Bottlenecks

The Svalbard population in the best‐supported model derived from the Nana lineage after the Nana‐Tundrarum admixture event, which supports previous genetic data that found Svalbard was predominantly colonised from north‐western Russia (Alsos et al. 2007, 2015). The divergence of the Svalbard population from the mainland population was estimated to occur in the mid‐Holocene between 6.2 ka (CI 1.27–9.86) and 4.4 ka (CI 0.908–7.04 ka). However, the first occurrence of *B. nana* in Svalbard inferred from sedaDNA is at ~7.5 ka (Alsos, Ehrich, et al. 2016), during a period of milder and wetter conditions (van der Bilt et al. 2019; Mangerud and Svendsen 2018; Voldstad et al. 2020), which seem to have been suitable for colonisation, establishment and population growth beyond the restricted distribution in which *B. nana* is found in Svalbard today (Alsos et al. 2002; Alsos, Sjögren, et al. 2016).

A strong population bottleneck and associated loss in genetic variation (Nei et al. 1975) can cause a more recent apparent coalescence of lineages compared with neutral expectations (Gattepaille et al. 2013). Pollen records suggest that *B. nana* underwent range contraction and population bottlenecks during the late Holocene cooling in Svalbard (Alsos et al. 2015; Alsos, Sjögren, et al. 2016; Birkeland et al. 2017), although our models were unable to differentiate between scenarios with and without a late Holocene population bottleneck (model set 8.5: Figure S8, Figure S9, Table S6). Similar to other studies, we found relatively low genetic diversity in Svalbard populations (Alsos et al. 2002, 2007; Eidesen et al. 2015). Our Svalbard divergence date therefore likely reflects the climate‐related bottleneck, rather than initial colonisation of the archipelago.

### 4.5 Model Assumptions and Data Uncertainty

The above model of population changes over the Quaternary must be conditioned on the uncertainty inherent in the climatic data, ice sheet reconstructions (Batchelor et al. 2019; Miller et al. 2010), and the time estimates derived from coalescent modelling. The challenges of estimating the generation time of dwarf birch species (see Appendix S7) led to uncertainty in estimating time in calendar years. Estimates spanning a range of plausible generation times helped to capture some of this uncertainty. The use of known dates of population events inferred from fossil pollen, macrofossil and sedaDNA records to calibrate population events could help address this issue (Borrell et al. 2018; Tsuda et al. 2017). Together, these sources of uncertainty contributed to the wide confidence intervals of demographic events presented in this study. Wider confidence intervals are returned earlier in the Quaternary in our model and were narrower in more recent time periods. This is similar to the greater uncertainty present in ice sheet reconstructions during this time period (Batchelor et al. 2019). Therefore, as with any analysis of demographic history and past climate change, our model of the demographic history of dwarf birch species and association with large‐scale environmental changes should be interpreted with caution, particularly in the early Quaternary. Uncertainty is also added by uneven sampling coverage across the Arctic, meaning that some gene flow routes, hybrid zones and refugia may not be captured with the available data.

Furthermore, although RAD‐seq offers deep sequencing coverage and a breadth of loci across the genome (Harvey et al. 2016), other characteristics of RAD‐seq data and analysis may bias demographic inference. RAD‐seq relies on restriction sites, meaning that loci are lost when a mutation disrupts a cut site. Distantly related or hybrid samples therefore may share far fewer loci than closely related ones (Eaton et al. 2017). In our dataset the putative hybrids—later removed from the analysis—had the highest proportion of missing loci.

Bioinformatic filtering introduces further variability into population genetic inference (Shafer et al. 2017). Stringent depth or missing‐data thresholds can discard loci that carry phylogeographically informative mutations, while lenient filtering can introduce error‐prone sites (Huang and Knowles 2016). We therefore employed intermediate filters to maximise informative variation while minimising noise.

### 4.6 Conclusions: Dwarf Birch Response to Climate Changes in the Arctic

We inferred the Quaternary population history of dwarf birch species, key Arctic tundra shrubs, by testing alternative evolutionary scenarios. The correspondence between the timing of population events and major ice sheet changes suggests a complex response of dwarf birch species to past climate change, with ice sheets acting alternately as a barrier or as an enabler of population mixing. Early Quaternary inter‐ and intraspecific divergences appear to be associated with climatic deterioration and extensive ice sheet coverage, suggesting isolation in separate refugia. Later admixture events between these divergent lineages occur during periods of climatic transition and are independent of absolute climate state, similar to Quaternary admixture events between polar bears ( *Ursus maritimus* L.) and brown bears ( *Ursus arctos* L.) (Wang et al. 2022). Rapid variability between climatic extremes was therefore a key feature that drove the generation of genetic diversity in the Quaternary (Brochmann and Brysting 2008). Dwarf birches do not appear to follow a simple glacial refugial isolation‐postglacial expansion model, which raises further questions about their eco‐evolutionary responses to present and future climate change (Mekonnen et al. 2021). Evidence from the pollen records of the last glacial period suggests that the climate and ecological conditions in ice‐free areas are also likely important mediators of population connectivity. Future research could therefore examine the effect of abiotic conditions such as geological substrate and precipitation, as well as biotic conditions such as competition, fragmentation and herbivory, on population connectivity. Taking the uncertainties inherent in the reconstruction and modelling of past populations and climate into account, the correspondence of multiple population events with multiple climatic events is indicative of an important role of the Pleistocene glacial cycles in influencing the evolution and population dynamics of dwarf birch species.

Dwarf birch is currently expanding in the Arctic as part of ongoing shrubification in response to climate warming (Ropars et al. 2015; Ropars and Boudreau 2012; Tape et al. 2006; Tremblay et al. 2012), and a further increase in Arctic woody shrub cover is predicted (Pearson et al. 2013). Climate‐induced range expansion (Parmesan 2006; Parmesan and Yohe 2003), in addition to altered growing seasons and phenologies (Cleland et al. 2007), can remove reproductive barriers and increase contact between species. In the coming decades, climate change may therefore increase gene flow and admixture between populations of the same species and hybridisation between different species.

The response of dwarf birch to climate change may be complex, since contemporary shrub expansion appears to be currently elevational rather than northward (Myers‐Smith et al. 2011; Myers‐Smith and Hik 2018), although this mismatch is likely due to different timescales involved in short distance elevational vs. longer distance latitudinal expansions. Furthermore, the borealisation of the Arctic may ultimately reduce habitat available for *B. nana* and *B. glandulosa* as other shrub and tree *Betula* species move northward (Reji Chacko et al. 2023; Berner and Goetz 2022). Northward dwarf birch expansion may be limited by lags in soil development, biotic interactions (e.g., competition) or dispersal barriers. In the longer run, the above processes might result in a net range contraction, fragmentation and genetic diversity loss for dwarf birch species (Alsos et al. 2012). These trends may also cause the appearance of isolated, dwarf birch hothouse refugia in the High Arctic and associated allopatric diversification and/or local extinctions. As suggested by our analysis, the response of dwarf birch to anthropogenic climate change will likely involve complex effects on range shifts, population connectivity, gene flow and genetic diversity.

## Author Contributions

M.D. conceptualised the study and designed the research with input from M.M.‐F. and P.B.E. M.M.‐F. designed and coordinated data collection of the wider project. D.A., J.A., B.C.F., M.G., T.T.H., S.R.K., T.K., M.L., M.M.L., I.M.‐S., J.P., C.R., G.S.‐S., M.Sl., S.S., A.S., J.D.M.S. M.Sp. and M.W. contributed to data collection. J.B. advised on DNA extraction, sequencing and demographic modelling approaches. M.D. performed DNA extraction, data processing and all data analyses. M.D. drafted the article with input from M.M.‐F. and E.E.S. All authors commented on the manuscript, contributing to draft revisions.

## Disclosure

Benefit‐sharing statement: A research collaboration was developed with scientists providing genetic samples; all collaborators are included as co‐authors. The results of the research have been shared with the providers and the broader scientific community. Benefits from this research accrue from the sharing of our data and results on public databases, as described above.

## Conflicts of Interest

The authors declare no conflicts of interest.

## Supporting information


**Data S1:** mec70082‐sup‐0001‐Supinfo.pdf.

## Data Availability

The genetic data for this study have been deposited in the European Nucleotide Archive (ENA) at EMBL‐EBI under accession number PRJEB67938. Data derived from the Natural History Museum at the University of Oslo samples are published for non‐commercial use only. Utilisation by third parties for purposes other than non‐commercial scientific research may infringe the conditions under which the genetic resources were originally accessed and should not be undertaken without contacting the corresponding author of the paper and/or seeking permission from the original provider of the genetic material. The ice sheet data that support the findings of this study are available at identifiers 10.17605/OSF.IO/7JEN3, https://doi.org/10.1016/j.quascirev.2020.106223, https://doi.org/10.1016/j.earscirev.2021.103875. Data were obtained from the Neotoma Paleoecology Database (http://www.neotomadb.org) and its constituent database, the European Pollen Database. The work of data contributors, data stewards and the Neotoma community is gratefully acknowledged. Additional data and code are available at DRYAD repository DOI: 10.5061/dryad.3xsj3txtm.
