# Supplementary material for: Molecular Footprints of Quaternary Climate Fluctuations in the Circumpolar Tundra Shrub Dwarf Birch
Source: Mol Ecol. 2025 Sep 2;34(19):e70082. doi: 10.1111/mec.70082 (PMC12456113; doi:10.1111/mec.70082)
Supplement: Supplementary file 1 — Data S1: mec70082‐sup‐0001‐Supinfo.pdf. [file MEC-34-e70082-s001.pdf]

Molecular footprints of Quaternary climate fluctuations in the circumpolar tundra shrub dwarf birch: Supplementary information

Maria Dance[1] (corresponding author); Scott Polar Research institute, University of Cambridge, Cambridge, UK; School of Geography and the Environment, University of Oxford, Oxford, UK; The University Centre in Svalbard, Longyearbyen, Norway; mariaemilyd@gmail.com

Erin E. Saupe; Department of Earth Sciences, University of Oxford, Oxford, UK; eesaupe@gmail.com

James Borrell; Royal Botanic Gardens, Kew, London, UK; J.Borrell@kew.org

Pernille Bronken Eidesen[2]**;** The University Centre in Svalbard, Longyearbyen, Norway; p.b.eidesen@ibv.uio.no

Daniel Ackerman; University of Minnesota, Minneapolis, USA; danackerman5@gmail.com

Jakob Assmann[3]**;** The University of Edinburgh, Edinburgh, UK; jakob.assmann@uzh.ch

Bruce C. Forbes; Arctic Centre, University of Lapland, Rovaniemi, Finland; bruce.forbes@ulapland.fi

Marina Gurskaya; Institute of Plant and Animal Ecology, UB RAS, Yekaterinburg, Russia; marina_gurskaya@mail.ru

Toke T. Høye; Department of Ecoscience, Aarhus University, Aarhus, Denmark; tth@ecos.au.dk

Stein R. Karlsen; NORCE Norwegian Research Centre, Tromsø, Norway; skar@norceresearch.no

Timo Kumpula; Department of Geographical and Historical Studies, University of Eastern Finland, Kuopio, Finland; timo.kumpula@uef.fi

Mariusz Lamentowicz; Faculty of Geographical and Geological Sciences, Adam Mickiewicz University, Poznań, Poland; mariuszl@amu.edu.pl

Michael M Loranty; Department of Geography, Colgate University NY, New York, USA; mloranty@colgate.edu

---

[1] Present address: Scott Polar Research institute, University of Cambridge, Cambridge, UK
[2] Present address: Department of Biosciences, University of Oslo, Oslo, Norway
[3] Present address: Department of Evolutionary Biology and Environmental Studies, University of Zurich, Zurich, Switzerland

Isla Myers-Smith[4]**;** The University of Edinburgh, Edinburgh, UK; isla.myers-smith@ubc.ca

Janet Prevéy; WSL Institute for Snow and Avalanche Research SLF, Davos, Switzerland; janetprevey@gmail.com

Christian Rixen; WSL Institute for Snow and Avalanche Research SLF, Davos, Switzerland; Climate Change, Extremes and Natural Hazards in Alpine Regions Research Center CERC, Davos, Switzerland; rixen@slf.ch

Gabriela Schaepman-Strub; Department of Evolutionary Biology and Environmental Studies, University of Zurich, Switzerland; gabriela.schaepman@ieu.uzh.ch

Michał Słowiński; Institute of Geography and Spatial Organization Polish Academy of Sciences (IGSO PAS), Warsaw, Poland; michal.slowinski@geopan.torun.pl

Sandra Słowińska; Institute of Geography and Spatial Organization Polish Academy of Sciences (IGSO PAS), Warsaw, Poland; s.slowinska@twarda.pan.pl

Aleksandr Sokolov; Arctic Research Station of the Institute of Plant and Animal Ecology, UB RAS, Labytnangi, Russia;

James D. M. Speed; Department of Natural History, NTNU University Museum, Norwegian University of Science and Technology, Trondheim, Norway; james.speed@ntnu.no

Marcus Spiegel; School of Geography and the Environment, University of Oxford, Oxford, UK; marcus.p.spiegel@gmail.com

Martin Wilmking; Institute of Botany and Landscape Ecology, University of Greifswald, Greifswald, Germany; wilmking@uni-greifswald.de

Marc Macias-Fauria; Scott Polar Research Institute, University of Cambridge, Cambridge, UK[5]; School of Geography and the Environment, University of Oxford, Oxford, UK; mm2809@cam.ac.uk

[4] Present address: Department of Forest and Conservation Sciences, University of British Columbia, Vancouver, Canada
[5] Present address: Scott Polar Research institute, University of Cambridge, Cambridge, UK

Contents

Appendix S1: Sampling protocol 3

Appendix S2: RAD Sequencing and Bioinformatics 8

Appendix S3: Hybrid populations 10

Appendix S4: Two-stage SNP filtering 11

Appendix S5: Population assignment for demographic modelling 13

Appendix S6: Model set scenario details and DIYABC RF model choice output 15

Appendix S7: DIYABC RF parameter estimation and generation time 38

Appendix S8: Population genetic structure and diversity 39

Appendix S9: DIYABC RF parameter estimates 47

Appendix S10: Map of dwarf birch Quaternary population history 53

Appendix S11: A non-toxic DNA extraction protocol from dwarf birch herbarium specimens for next-generation sequencing 54

References 59

Appendix S1: Sampling protocol

Identification of individual dwarf birch plants can be difficult, as a genetic individual can extend over many metres due to vegetative reproduction. This is common at northern range limits where climate currently limits sexual reproduction (Alsos et al., 2002; de Groot et al., 1997). To avoid resampling clonal or cryptic individuals, samples were collected over an approximately 200 metre area with a minimum distance of 10 metres between sampled individuals in continuous growth and 5 metres when growth was in distinct patches. Twig and leaf samples were collected from up to 30 individuals. GPS coordinates were obtained for each site. Samples were dried in silica gel, drying ovens, or air-dried, and stored in plastic zip lock bags, or paper bags/envelopes. Samples were shipped and stored in a 4-8°C cold room until processing in 2021. Sixty sites were selected for DNA extraction on the basis of tissue quantity and quality. To fill geographical sampling gaps, silica-dried specimens from seven populations that were also sequenced in Eidesen et al. (2015) were obtained with the permission from the NHMO DNA Bank of the Natural History Museum, University of Oslo. These samples were collected between 2002-2004 with a defined sampling protocol where each sample was collected 25 m apart along a 250 m transect.

Table S1. Dwarf birch sampling site collection metadata. Sites which were used in the Eidesen et al. (2015) study are marked with † and for these populations the 'Site ID' column includes the population name from the original study, and the 'Number of Individuals sequenced' column includes the original accession numbers of the specimens which were analysed in this study.

Site ID	Taxon (field ID)	Country	Region	Locality	Collection year	Collector	Latitude (decimal degrees)	Longitude (decimal degrees)	Number of individuals sequenced	Used in analysis
BN01	*B. nana*	USA	Alaska	Itkillik	2015	DA	68.6422	-149.64	4	Y
BN02	*B. nana*	USA	Alaska	Chandalar	2015	DA	68.0678	-149.599	6	Y
BN03	*B. nana*	USA	Alaska	Dalton Mountain	2015	DA	68.1719	-149.44	6	Y
BN04	*B. nana*	USA	Alaska	Kuparuk River	2015	DA	68.6486	-149.403	7	Y
BN05	*B. nana*	USA	Alaska	Roche Mountonee	2015	DA	68.3747	-149.313	6	Y
BN06	*B. nana*	USA	Alaska	Bunny Haven	2015	DA	68.7657	-148.9	6	Y
BN07	*B. nana*	Canada	Labrador	Komaktorvik River	2015	DM; LH	59.204	-64.0985	6	Y
BN08	*B. nana subsp. exilis*	Russia	Sakha	Kytalyk1	2015	GSS; RS	70.8284	147.4703	6	Y
BN09	*B. nana subsp. exilis*	Russia	Sakha	Kytalyk2	2015	GSS; RS	70.831	147.4746	6	Y
BN10	*B. nana*	Norway	Viken	Hol	2015	JDMS	60.7052	7.9355	6	Y
BN12	*B. nana*	USA	USA	Atqasuk	2015	JP; SW	70.4833	-157.417	4	Y
BN13	*B. nana*	Canada	Labrador	Torngats Basecamp	2015	LH	58.4581	-62.8087	6	Y
BN14	*B. nana*	Canada	Labrador	Nain	2015	LH	56.5439	-61.6993	6	Y

BN15	*B. nana*	Finland	Lapland	Näkkälä	2015	MMF	68.641	24.7725	5	Y
BN16	*B. nana*	Norway	Finnmark	Nordkapp	2015	MMF	71.1087	25.7973	6	Y
BN17	*B. nana*	Finland	Lapland	Saariselkä	2015	MMF	68.4352	27.4351	6	Y
BN18	*B. humilis*	Russia	Urals	Nurgush Mountain	2015	MG; MW	54.8296	59.1588	6	Y
BN21	*B. nana*	USA	Alaska	East Fork Chandalar River	2015	MW	68.71	-144.825	6	Y
BN22	*B. nana*	Poland	Kujawsko-Pomorskie	Linje	2015	MS, ML, SS	53.1876	18.3096	4	N
BN23	*B. nana*	USA	Alaska	Nome	2015	MML	64.508	-165.297	5	Y
BN24	*B. nana*	USA	Alaska	Kuzitin	2015	MML	65.2293	-164.827	6	Y
BN25	*B. nana*	USA	Alaska	Old Man	2015	MML	66.4501	-150.619	5	Y
BN29	*B. nana*	USA	Alaska	SAG	2015	MML	69.4254	-148.697	3	Y
BN30	*B. nana*	USA	Alaska	Fox	2015	MML	64.9506	-147.618	6	Y
BN31	*B. nana subsp. exilis*	Russia	Sakha	Chersky	2015	MML	68.7521	161.4686	6	N
BN32	*B. nana subsp. exilis*	Russia	Sakha	Krutaya Drisva	2015	MML	69.3452	161.5314	6	Y
BN33	*B. nana subsp. exilis*	Russia	Sakha	Ambarchik	2015	MML	69.6215	162.3131	6	Y
BN34	*B. nana*	USA	Alaska	Healy	2015	MML	63.8782	-149.253	6	Y
BN37	*B. nana*	Svalbard	Norway	Endalen & Janssonhaugen	2015	SRK	78.1868	15.7632	7	Y
BN38	*B. nana*	Norway	Troms	Kvaløya	2015	SRK	69.759	18.8524	6	Y
BN39	*B. nana*	Norway	Finnmark	Vadsø	2015	SRK	70.0712	29.8731	5	Y
BN40	*B. nana*	USA	Alaska	St. Mary's	2015	SN	62.0555	-163.259	6	Y
BN42	*B. nana ?*	Finland	Lapland	Kilpisjärvi	2015	TK	68.9441	20.9116	6	Y

BN43	*B. nana*	Finland	Karjala	Joensuu	2015	TK	62.6691	30.017	6	Y
BN44	*B. nana*	Norway	Telemark	Lifjeld	2015	TTH	59.486	8.998	2	Y
BN45	*B. nana*	Switzerland	Neuchâtel	Les Ponts-de-Martel	2015	CR	46.9842	6.7167	6	Y
BN46	*B. glandulosa*	Canada	British Columbia	Og Lake	2015	MMF	50.9516	-115.635	6	Y
BN47	*B. nana*	Russia	Sakha	Rodinka Mountain	2016	TK	68.7337	161.5375	6	N
BN49	*B. glandulosa*	Canada	Yukon Territories	Kluane - Pika Valley	2015	JA	61.22145	-138.286	6	Y
BN51	*B. nana*	Canada	Yukon Territories	QHI - Hawk Valley	2015	JA	69.58539	-138.922	6	Y
BN54	*B. glandulosa*	Canada	Northwest Territories	Yellowknife - Bog	2015	HT	62.45721	-114.533	5	N
BN55	*B. glandulosa*	Canada	Northwest Territories	Yellowknife - Site 2	2015	HT	62.69975	-116.131	3	N
BN61	*B. nana*	Russia	Yamal	Erkuta Tundra Monitoring site	2021	MSp	68.239	69.142	4	Y
BN62† AK-3701	*B. nana*	Russia	Nenetsky AO	Malozemelskaya tundra. Nenetskaya Gryada, Korovinskaya Guba; 5.5-8 km W Cape Seduysky	2004	DE	68.336	53.3	3 (Acc. # O-DP-1725/1-T, O-DP-1727/1-T, O-DP-1728/1-T)	Y

				Nos, c. 0-3 km W to NW the shore						
BN63† AK-205	*B. nana*	Russia	Taimyrsky AO	Ary-Mas nature reserve, (c. 50-60 km NNW Khatanga), right riverside of Novaya, along the river and up to c. 3 km S of river	2004	PS; AT	72.46444	101.8636	1 (Acc # O-DP-2211/1-T)	Y
BN64† AK-3885	*B. nana subsp. exilis*	Russia	Yakutia	Lena R. west bank: Chekurovka village, surroundings of settlement	2004	HS; RE	71.04833	127.5233	3 (Acc # O-DP-25376/1-T, O-DP-25378/1-T, O-DP-25380/1-T)	Y
BN67† AK-1241	*B. nana*	Greenland	Qeqertarsuaq (Disko), Qeqertalik	5-10 km from Arctic Station (coordinates are for station)	2003	BJG; GB	69.25	-53.5667	1 (Acc # O-DP-1560/1-T)	Y
BN68† AK-125	*B. nana*	Greenland	Zackenberg	South of the landingstrip close to "Kjovedammen" and "Teltdammen".	2002	KA	74.46667	-20.55	1	Y

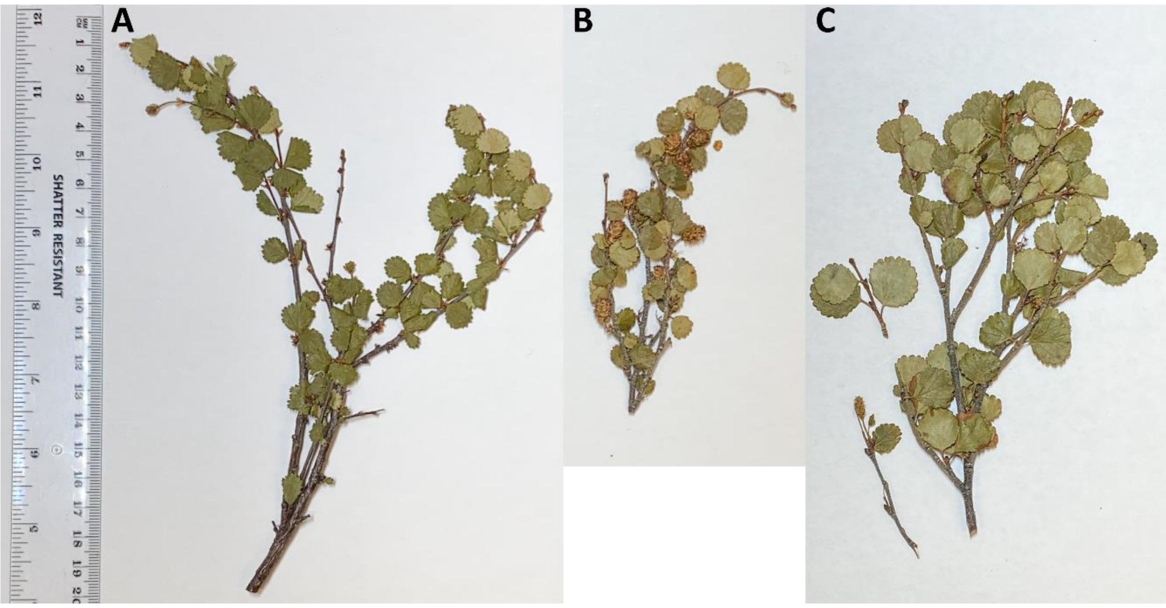

Figure S1. *Betula nana* L. and *Betula glandulosa* Michx. specimens. *Betula nana* (diploid with 2n = 28) is a characteristic shrub of peat bog and tundra environments. It is monoecious, wind-pollinated, wind-dispersed, and has a prostrate or upright growth habit up to 1 metre in height (de Groot et al., 1997; Jadwiszczak et al., 2012). *Betula nana* is sometimes divided into two species: (**A**) *Betula nana* subsp. *nana* (Sukaczev) Hult. and (**B**) *Betula nana* subsp. *Exilis* (Sukaczev) Hult. *Betula glandulosa* (diploid with 2n = 28) (**C**) has similar habitat preferences to *B. nana* but is less cold tolerant (de Groot et al., 1997). The species grows as tall, upright shrubs (up to 3 metres in height) in the core of its range, but *B. glandulosa* and *B. nana* both become lower and more prostrate with smaller leaves in harsher environmental conditions and at their northern range limits (de Groot et al., 1997). Scale in centimetres and inches on the left. Specimens from sampling sites BN38 (**A**), BN32 (**B**), and BN21 (**C**), are shown as their genetic group matched their taxonomic group – see **Table S1** for locality information.

Appendix S2: RAD Sequencing and Bioinformatics

Previous RAD sequencing of *Betula nana* used the cleavage enzyme *PstI,* which has approximately 70,954 cut sites in the draft genome (Borrell et al., 2018; Wang et al., 2013). Here we used the cleavage enzyme *SfbI*, which produces a subset of the same cut sites since it has a 2 bp longer recognition sequence. One library was prepared per sample, with unique 10-base barcodes ligated via adaptors to *SfbI* digested genomic DNA. Each plate contained one blank control in differing locations in case of problems with plate identification, and one

internal control. To ensure consistency in the loci sequenced across samples and to reduce the number of missing genotypes downstream, fragments between 300 and 500 bp were selected and amplified with PCR. After sequencing and demultiplexing libraries, barcode sequences were checked for errors using the process_radtags module of Stacks v 2.60 (Catchen et al., 2011, 2013; Rochette et al., 2019). A quality threshold Phred score of 10 was used in sliding windows of 15% of the length of the read (19.8 bp in a 131 bp alignment) (Rochette et al., 2019). After combining the duplicated samples, the sequencing depth coverage for each individual and population was assessed; four individuals with fewer than 300,000 reads and two individuals with more than 4 million reads were removed from the analysis.

Table S2. List of sample DNA extracts which were duplicated and sequenced. Only samples which were sequenced more than once are listed.

Sampling site	Sample ID	Number of duplicates	Sampling site	Sample ID	Number of duplicates	Sampling site	Sample ID	Number of duplicates
BN01	0101	3	BN10	1001	3	BN37	3702	2
BN02	0206	3	BN10	1002	2	BN37	3703	2
BN03	0304	3	BN10	1003	2	BN39	3902	2
BN03	0301	2	BN10	1004	2	BN42	4205	3
BN03	0302	2	BN10	1005	2	BN43	4307	2
BN03	0303	2	BN10	1006	2	BN43	4308	2
BN03	0305	2	BN12	1204	2	BN43	4309	3
BN03	0306	2	BN13	1301	3	BN43	4310	2
BN04	0401	2	BN13	1304	2	BN43	4311	3
BN04	0402	2	BN13	1305	2	BN43	4312	2
BN04	0403	2	BN14	1402	3	BN44	4401	2
BN04	0405	2	BN14	1405	2	BN44	4402	2
BN04	0404	2	BN14	1409	2	BN45	4509	2
BN04	0407	2	BN14	1410	2	BN46	4606	2
BN05	0501	2	BN17	1702	2	BN46	4607	3
BN05	0502	2	BN17	1706	2	BN47	4707	2
BN05	0503	2	BN24	2403	2	BN49	4902	2
BN05	0504	2	BN24	2404	3	BN49	4903	2
BN06	0601	2	BN24	2406	2	BN49	4906	2
BN06	0602	2	BN24	2408	2	BN49	4907	2
BN06	0604	2	BN25	2501	2	BN51	5101	2
BN06	0605	2	BN25	2507	2	BN51	5102	2
BN06	0606	2	BN30	3002	2	BN51	5103	2
BN07	0701	2	BN30	3003	2	BN51	5104	2
BN07	0702	2	BN30	3005	2	BN51	5105	2
BN07	0704	2	BN30	3006	2	BN51	5106	3
BN07	0706	2	BN31	3108	2	BN54	5402	2

BN08	0801	2	BN32	3208	2	BN54	5403	2
BN08	0802	2	BN33	3301	3	BN54	5404	2
BN08	0803	2	BN33	3302	2	BN54	5409	2
BN08	0804	2	BN33	3303	2	BN55	5506	2
BN08	0805	3	BN33	3305	2	BN55	5507	3
BN09	0901	2	BN33	3306	2	BN61	6112	2
BN09	0904	2	BN34	3402	2	BN62	6204	2
BN09	0906	2	BN34	3408	3	BN68	6805	3
BN09	0907	2	BN37	3701	2			

Table S3. Samples removed from the analysis due to anomalously low or high coverage. Samples with <300,000 reads after alignment to a reference genome were removed ("low coverage") and samples with >1000 x coverage were removed ("high coverage").

Sampling site	Sample ID	Low Coverage	High coverage
BN01	0103	x	
BN22	2201	x	
BN22	2204	x	
BN23	2302	x	
BN49	4907		x
BN61	6112		x

Appendix S3: Hybrid populations

Initial analyses identified four sampling locations as potential hybrid populations with additional, unidentified *Betula* species: two *B. glandulosa* sampling locations near Yellowknife, NWT (BN54, BN55) and two *B. nana* sampling locations near Chersky, Sakha Republic (BN37, BN41). Collection metadata had indicated that these individuals may be hybrids due to their unusually large leaf morphology. Furthermore, populations had a substantial proportion of missing SNP loci (~25% compared with ~5% in other populations). Finally, ADMIXTURE (Alexander et al., 2009; Alexander & Lange, 2011) analyses including individuals from the species *B. humilis* Schrank. indicated four ancestral groups (K=4), with the four sampling sites containing a substantial proportion of ancestry from the same ancestral group as *B. humilis* (**Figure S2**). As these sites were spread across two continents outside the range of *B. humilis*, we hypothesised that different birch species were likely contributing to the hybridisation process in each location, and that the ADMIXTURE results identified these groups as the most divergent from others and so grouped them together. SNP genotyping and filtering steps was repeated without these sampling sites as they would have reduced the number of SNPs genotyped and filtered, and they were excluded from further analyses.

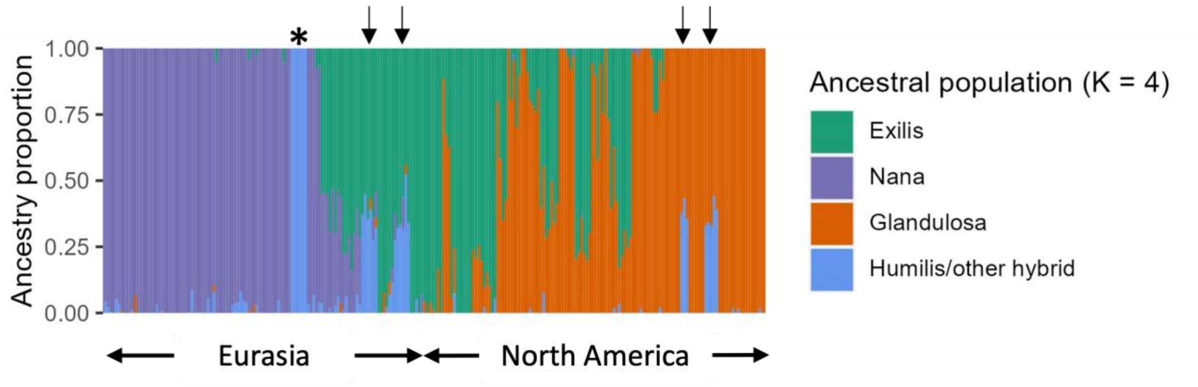

Figure S2. Population genetic structure inferred from ADMIXTURE analysis including potential hybrid populations (Alexander et al., 2009; 2011). Colour corresponds to the optimal number (lowest cross validation error) of proposed ancestral groups (K), with each dwarf birch individual plotted by longitude, represented by bars with total ancestry proportions. *Betula humilis* population (BN18) marked with asterisk (*). Potential hybrid populations marked with arrows, from left to right: BN31, BN47, BN54, BN55.

Appendix S4: Two-stage SNP filtering

Initial filtering suggested that a large number of SNPs were specific to certain sampling locations. To maximise the number of SNPs regional subsets of the data were filtered: a) the global dataset, b) North American sampling sites, c) Eurasian sampling sites, and d) western Eurasian sampling sites. To further keep as many SNPs as possible, a two-stage filtering step was created:

First stage

Loci were filtered with sampling locations treated as populations, except for the two Svalbard populations as they were both within the Adventdalen area (~13 km distant) which were merged due to the low number of samples per population. To minimise missing data, population filters were applied. Loci were retained that were present in 70% of individuals within a population, or present in 70% of individuals across populations. SNPs with a minor allele frequency (MAF) of <0.01 (SNPs where >99% of individuals share the same allele) were excluded to remove uninformative markers. The Populations module of Stacks v2.60 (Catchen et al., 2011, 2013; Rochette et al., 2019) can output phased haplotypes with multiple SNPs per locus, from which population estimates of expected heterozygosity (gene diversity) were extracted. Populations output was also exported taking the first SNP per locus as downstream analyses (ADMIXTURE, PCA) required unlinked alleles, from which rarefied allelic richness was extracted using hierfstat (Goudet et al., 2022) in R v4.0.3 (R Development Core Team, 2020).

To meet assumptions of PCA and ADMIXTURE analyses (Alexander et al., 2009; Alexander & Lange, 2011) the SNPs were filtered further to remove loci that were in Linkage Disequilibrium with nearby loci. Parameters for filtering were determined by calculating the average LD across intervals in the genome in 100 kb bins using PLINK v1.9 (Chang et al., 2015; Purcell & Chang, 2022) and haplotype variant call format files (VCFs). Visual inspection of the results identified a window size of 33 kb where LD decayed to a background rate of 0.1. VCF files were then filtered in windows of 33 kb sliding by 10 kb with a threshold of correlation

coefficient (r^2) > 0.2. SNPs were output as VCFs and converted to plink (bed) files (Chang et al., 2015; Purcell & Chang, 2022). LD-filtered SNPs were converted to DIYABC (Collin et al., 2021; Cornuet et al., 2014) SNP input format using custom R scripts.

Second stage

We used the software ADMIXTURE v 1.3.0 (Alexander et al., 2009; Alexander & Lange, 2011) to redefine input populations for a second run of the Populations module of stacks. We conducted ADMIXTURE analyses on the plink files of the global dataset and for each regional subset of the dataset. ADMIXTURE does not accept non-human chromosome names, so chromosome names were replaced with "0" in the first column of each bed file. ADMIXTURE was run with default parameters. We tested a range of 2-7 possible ancestral populations (K); the K with the lowest cross-validation error was chosen as the optimal value. We equated the number of ancestral populations K with the number of genetically distinct populations. New population maps for the Populations module were created based on the assignment to ancestral populations. Individuals were considered part of a population if they had >50% assignment probability.

The Populations module was repeated, with population filters on the new populations. Loci were retained that were present in 70% of individuals within a population, or present in 70% of individuals across populations. SNPs with a minor allele frequency (MAF) <0.01 were excluded to remove uninformative markers. Genetic summary statistics were calculated by population as before. LD filtering was repeated as before and SNPS output as VCFs and converted to plink (bed) files. The two-stage filtering resulted in a 4-16% increase in SNPs (**Table S4**).

Table S4. Results of two-stage SNP filtering using Stacks v2.60 populations module (Catchen et al., 2011, 2013; Rochette et al., 2019). Loci were subject to population filters, but not every locus had a variant site (SNP), hence the drop in number. Percentage increase refers to the increase in variant sites obtained from filtering stage two relative to filtering stage one. LD refers to Linkage disequilibrium.

**Dataset**	**Output**	**Number of loci**		**Number of variant sites after filtering stages**		**% Increase**	**Number of variant sites in LD**		**% Increase**
		Pre-filters	Post-filters	Filtering stage 1	Filtering stage 2		Filtering stage 1	Filtering stage 2	
America	First SNP per locus	53349	2165	1697	1721	1.41	834	864	3.60
America	Multiple SNPS	53349	2165	6301	6615	4.98	2564	2698	5.23

Eurasia	First SNP per locus	53349	2222	1778	1888	6.19	991	1072	8.17
Eurasia	Multiple SNPS	53349	2222	7232	7945	9.86	3295	3624	9.98
Western Eurasia	First SNP per locus	53349	2245	1747	1917	9.73	946	1040	9.94
Western Eurasia	Multiple SNPS	53349	2245	6185	7164	15.8	2551	2953	15.76
Global	First SNP per locus	53349	2180	1724	1744	1.16	1001	1041	4.00
Global	Multiple SNPS	53349	2180	7528	7858	4.38	3642	3830	5.16

Appendix S5: Population assignment for demographic modelling

Populations were assigned using ancestry coefficients from the regional ADMIXTURE analyses:

Species divergence model: three main populations were identified in the global ADMIXTURE analysis (Exilis, Nana, Glandulosa). Admixture between populations can affect patterns of relatedness between groups and divergence time estimates, so only individuals with >95% ancestry proportion from a single population were retained for these models.

American models: three main populations were identified in the ADMIXTURE analysis (Exilis, Glandulosa west and Glandulosa east). The extensive admixture between Exilis and Glandulosa west in present-day Alaskan populations was interpreted as potentially a longer period of range overlap and gene flow between exilis and glandulosa in Beringia. Our interpretation is supported by a recent genetic analysis of *B. glandulosa* and other North American shrub birch species including *B. nana*, which suggested that these species exchanged genetic material in the past and continue to do so through ongoing gene flow and hybridisation (Touchette et al., 2024). DIYABC can include discrete admixture and introgression events but not continuous gene flow (Collin et al., 2021; Cornuet et al., 2014). Therefore we treated the admixed populations as one effective population, with an admixture date representing the time when the admixture between exilis and glandulosa west began. Individuals with >95% ancestry proportion were assigned to the relevant population. Individuals with >5% and < 95% exilis were assigned to the "admixed population".

Eurasian models: Two populations were identified in the ADMIXTURE analysis (Exilis and Nana), with an additional population identified in the west Eurasian ADMIXTURE analysis that delineated the Svalbard population and was also introgressed into the Nana group, particularly in northeast Scandinavia and northwest Russia. This was suggestive of the "west Siberian group" previously hypothesised to be formed as a result of admixture between Exilis and Nana in west Siberia, and then isolated in a later glacial period to result in a genetically distinctive west Siberian population (Eidesen et al., 2015), sometimes recognised taxonomically as *Betula nana* L. *ssp. tundrarum* (Perfil.) Á.Löve & D.Löve (Elven et al., 2011). As the ancestry proportion of this population never exceeded 50% excepting in Svalbard, the west Siberian population was coded into model scenarios as an unsampled "ghost" population. Individuals with >50% exilis or nana proportion were assigned to exilis or nana, respectively. The Svalbard individuals were assigned to a unique population.

Global models: The global model used the same population groupings as the regional models, meaning that Eurasian individuals that were >50% exilis and American individuals that were >95% exilis were combined in an exilis population. Population genetic structure analyses found Exilis populations in eastern Russia and western Alaska to be very little differentiated, supporting grouping in one population.

Appendix S6: Model set scenario details and DIYABC RF model choice output

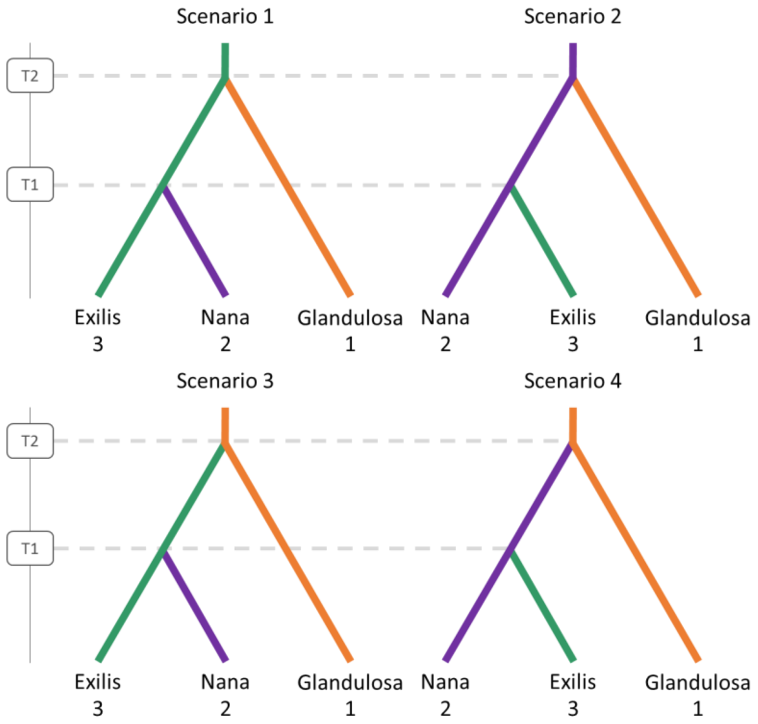

Figure S4. Global species divergence model (model set 4).

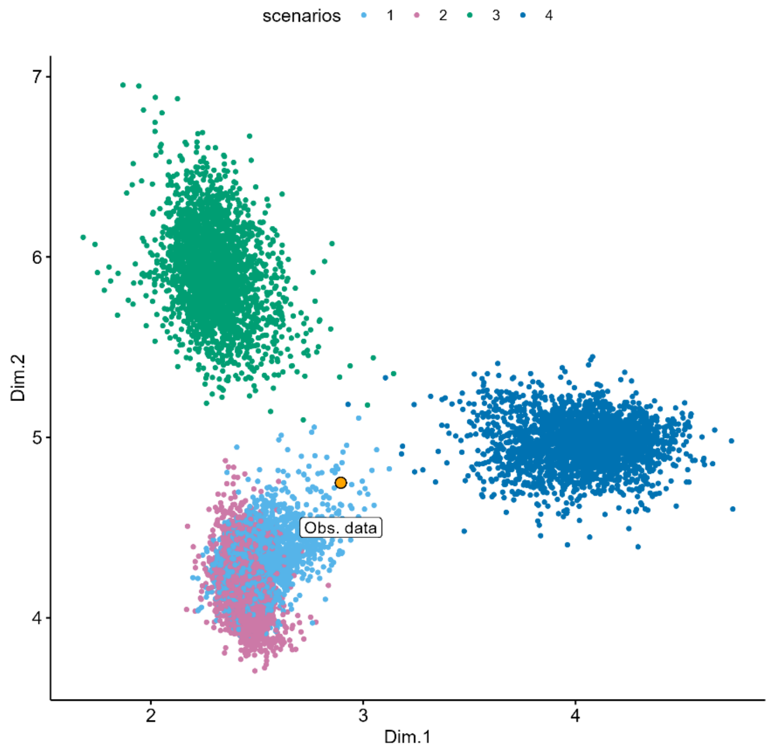

Figure S5. Projection of simulated datasets of global species divergence model (model set 4) on the first two axes of a Linear Discriminant Analysis. The location of the observed genetic data is indicated by the yellow circle. 10,000 datasets were used for model choice.

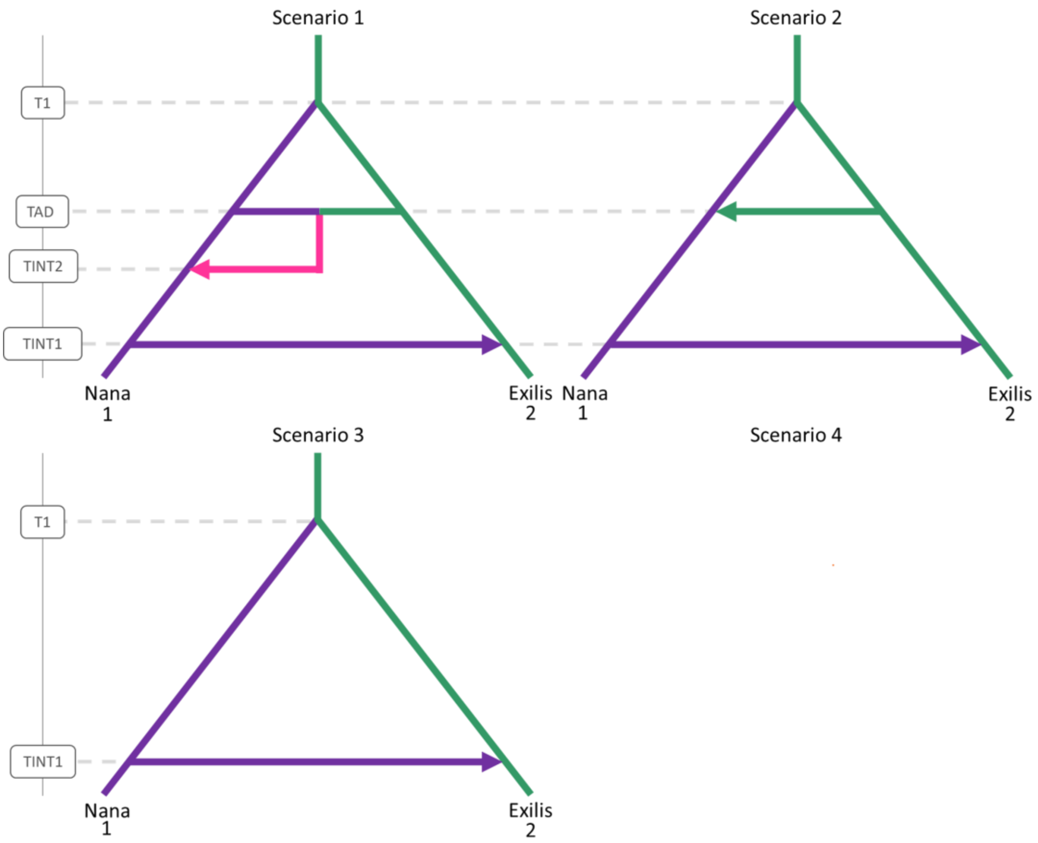

Figure S6. Eurasia model without Svalbard (model set 8.5).

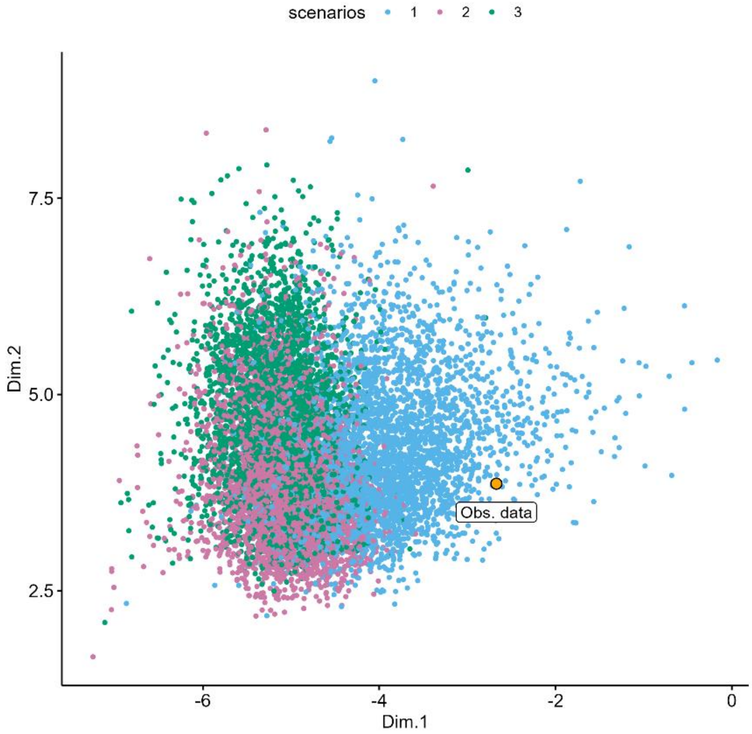

Figure S7. Projection of simulated datasets of Eurasian model without Svalbard (model set 8.5) on the first two axes of a Linear Discriminant Analysis. The location of the observed genetic data is indicated by the yellow circle. 10,000 datasets were used for model choice.

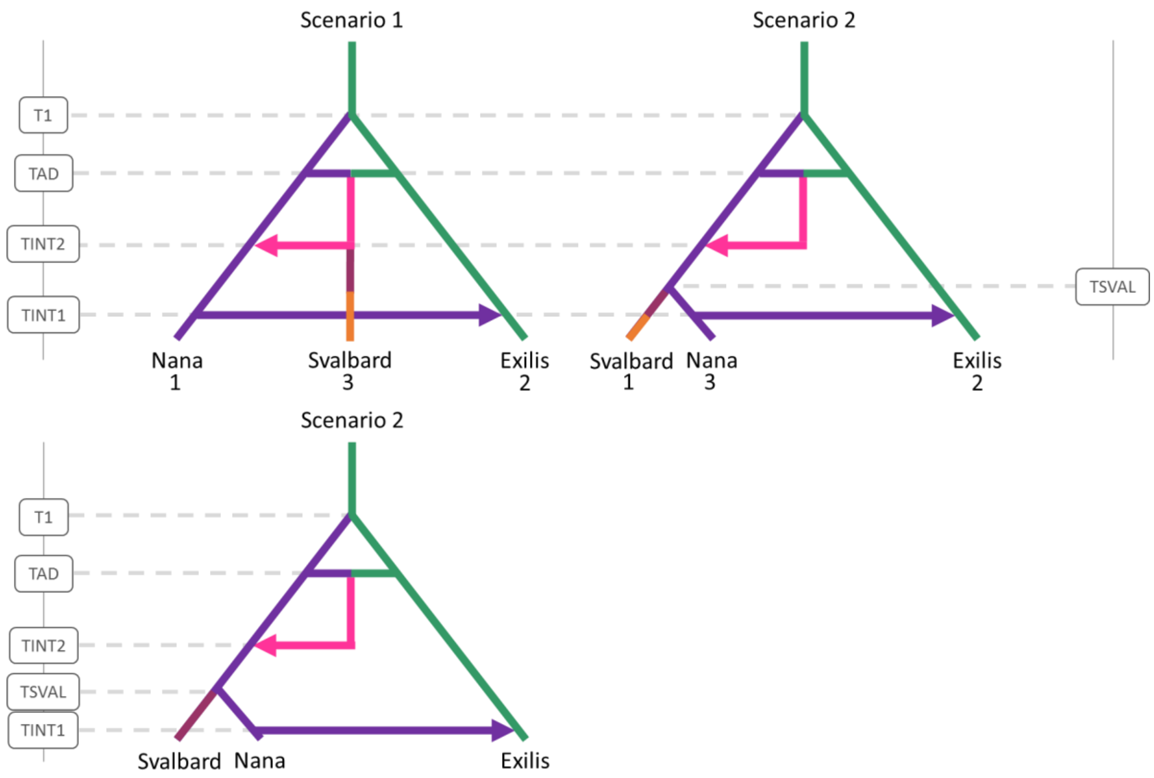

Figure S*8*. Eurasia model with Svalbard: model sets 9 (3 scenarios) and 10 (Scenario 1 and 3 only).

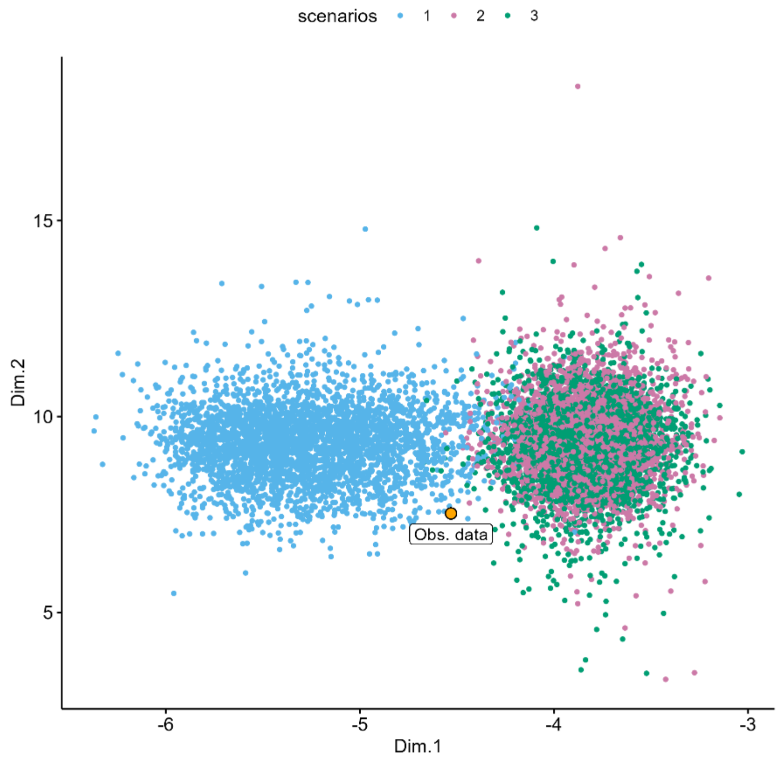

Figure S9. Projection of simulated datasets of Eurasian model with Svalbard (model set 9) on the first two axes of a Linear Discriminant Analysis. The location of the observed genetic data is indicated by the yellow circle. 10,000 datasets were used for model choice.

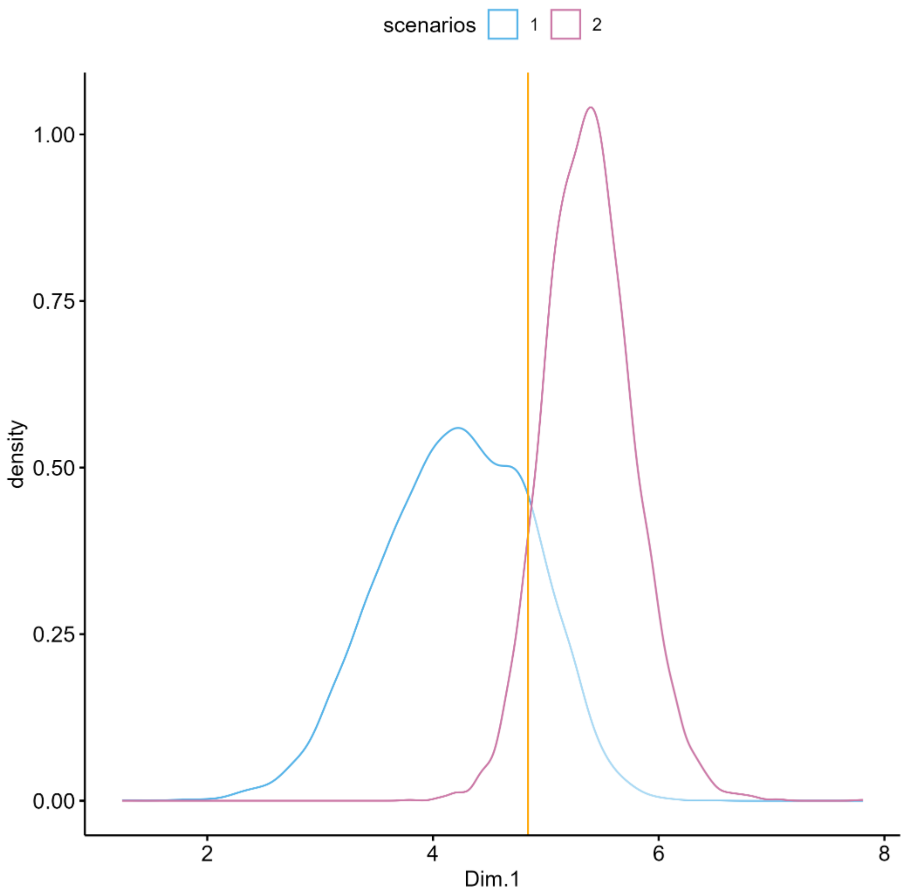

Figure S10. Projection of simulated datasets of Eurasian model without Svalbard (model set 10) on the first axis of a Linear Discriminant Analysis. The location of the observed genetic data is indicated by the solid orange line. 10,000 datasets were used for model choice.

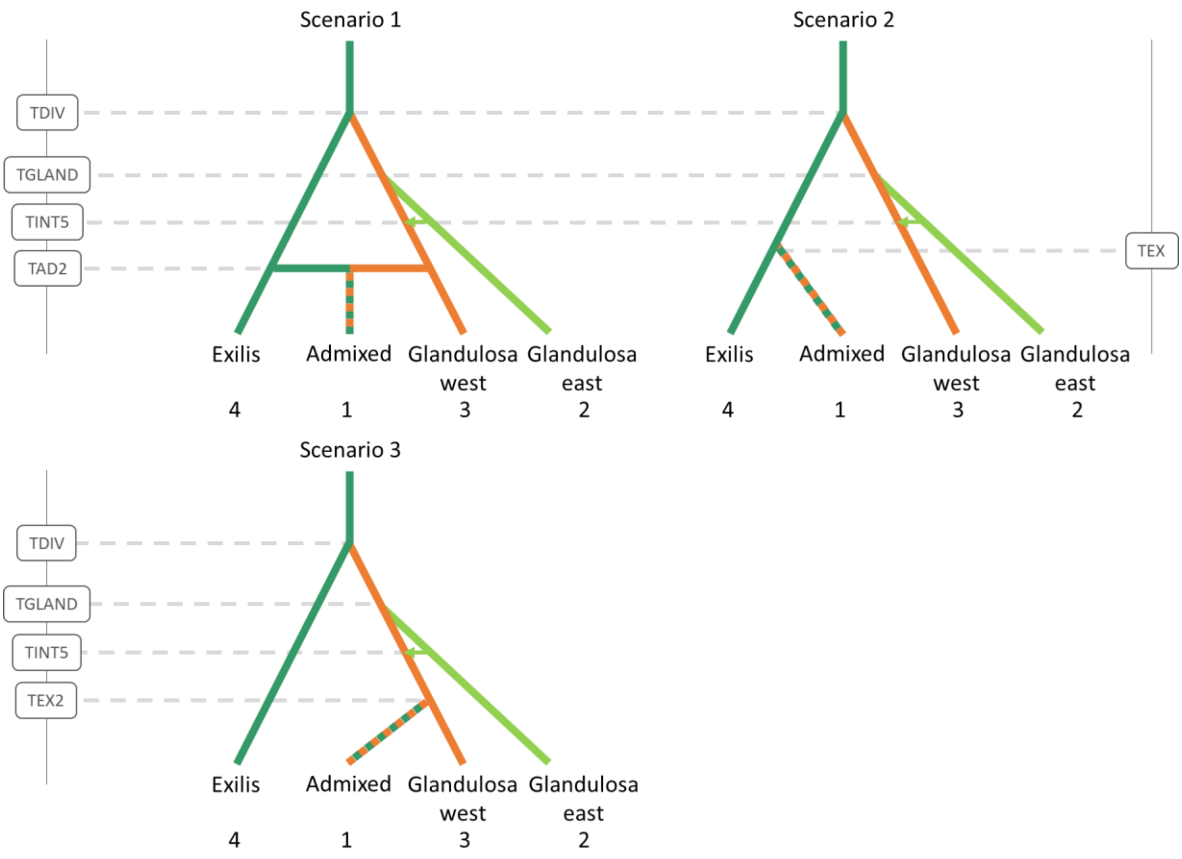

Figure S11. North American model (model set 14.1).

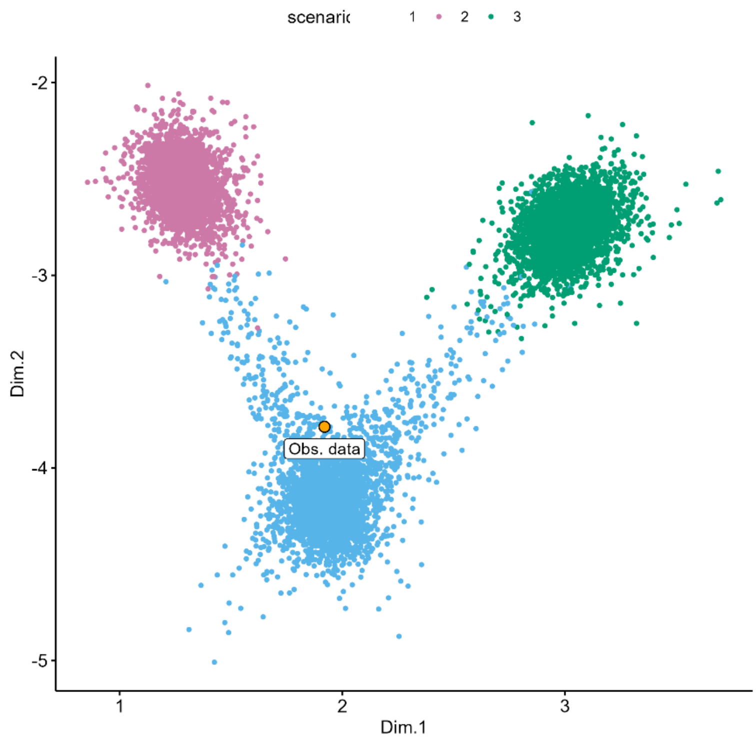

Figure S12. Projection of simulated datasets of North American model (model set 14.1) on the first two axes of a Linear Discriminant Analysis. The location of the observed genetic data is indicated by the yellow circle. 10,000 datasets were used for model choice.

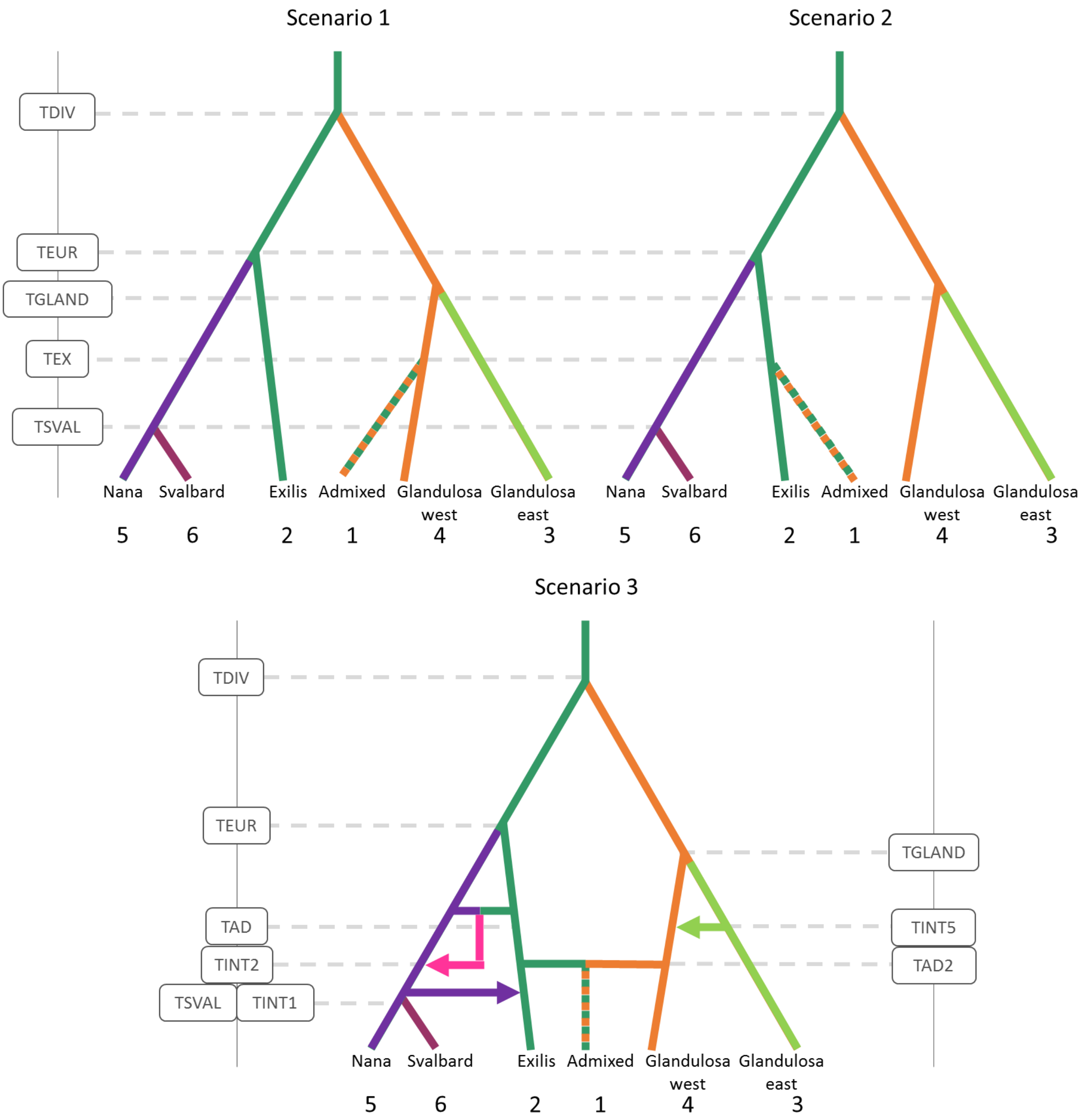

Figure S13. Global model (model set 20).

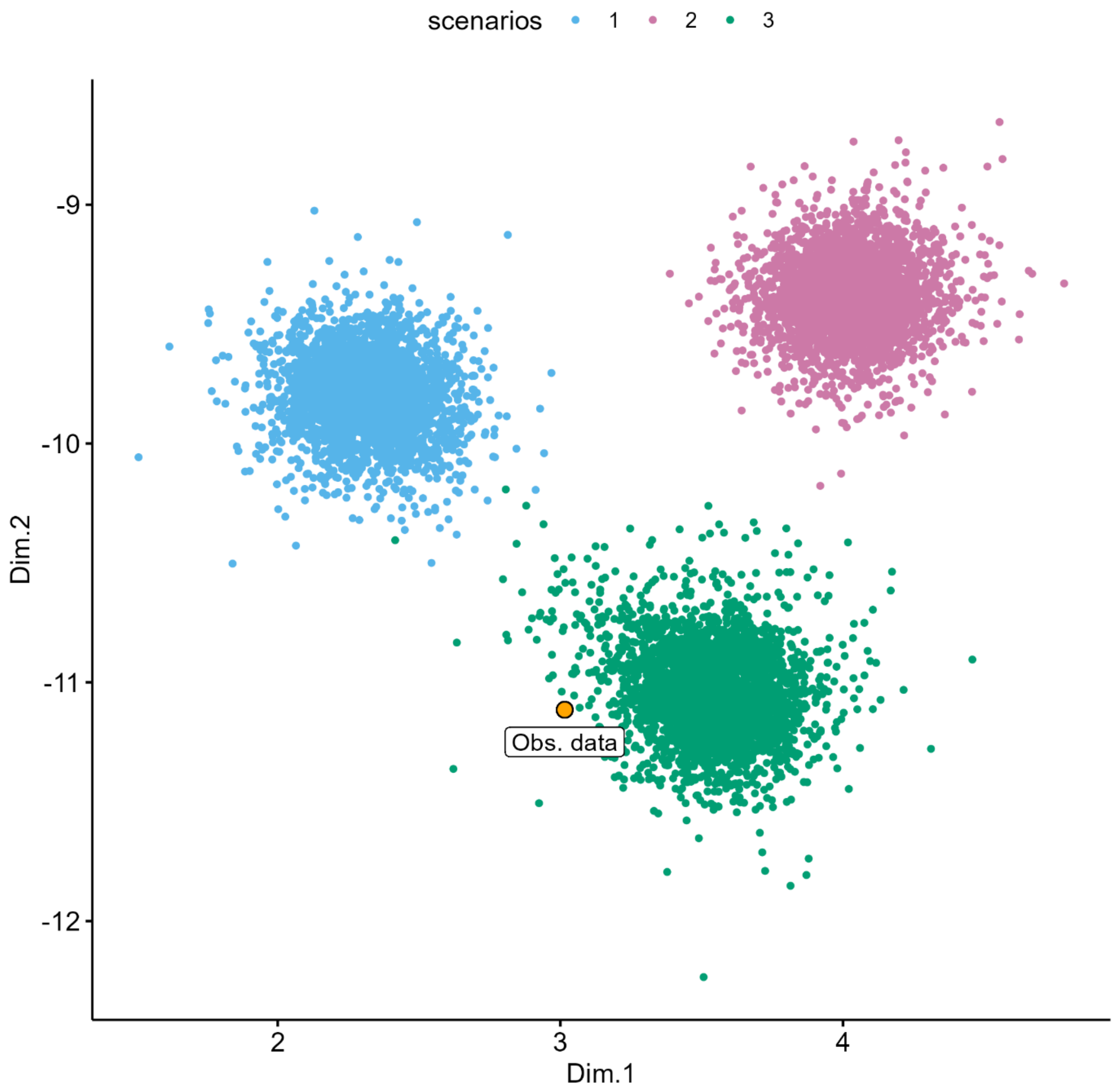

Figure S14. Projection of simulated datasets of global model (model set 20) on the first two axes of a Linear Discriminant Analysis. The location of the observed genetic data is indicated by the yellow circle. 10,000 datasets were used for model choice.

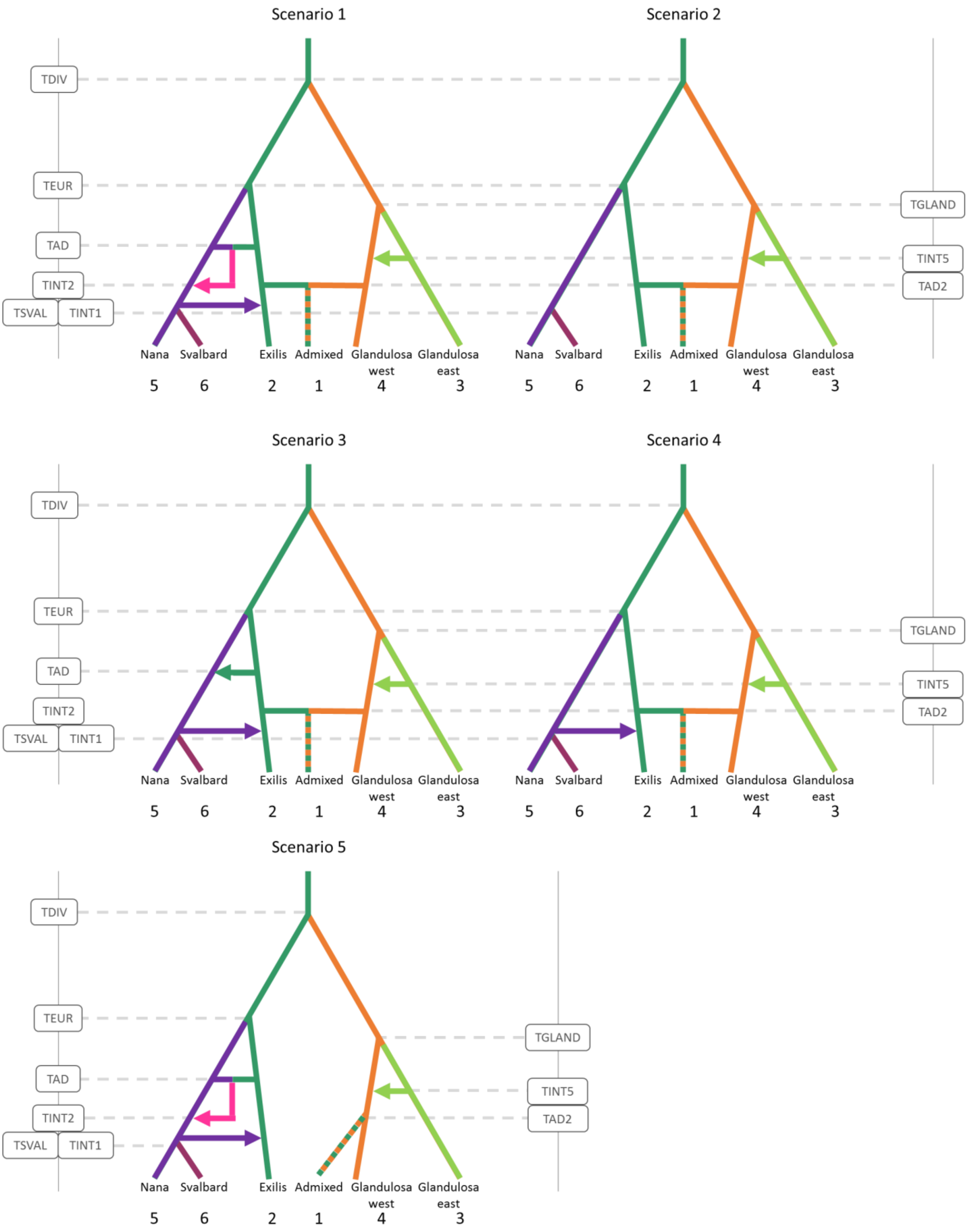

Figure S15. Global model (model set 21).

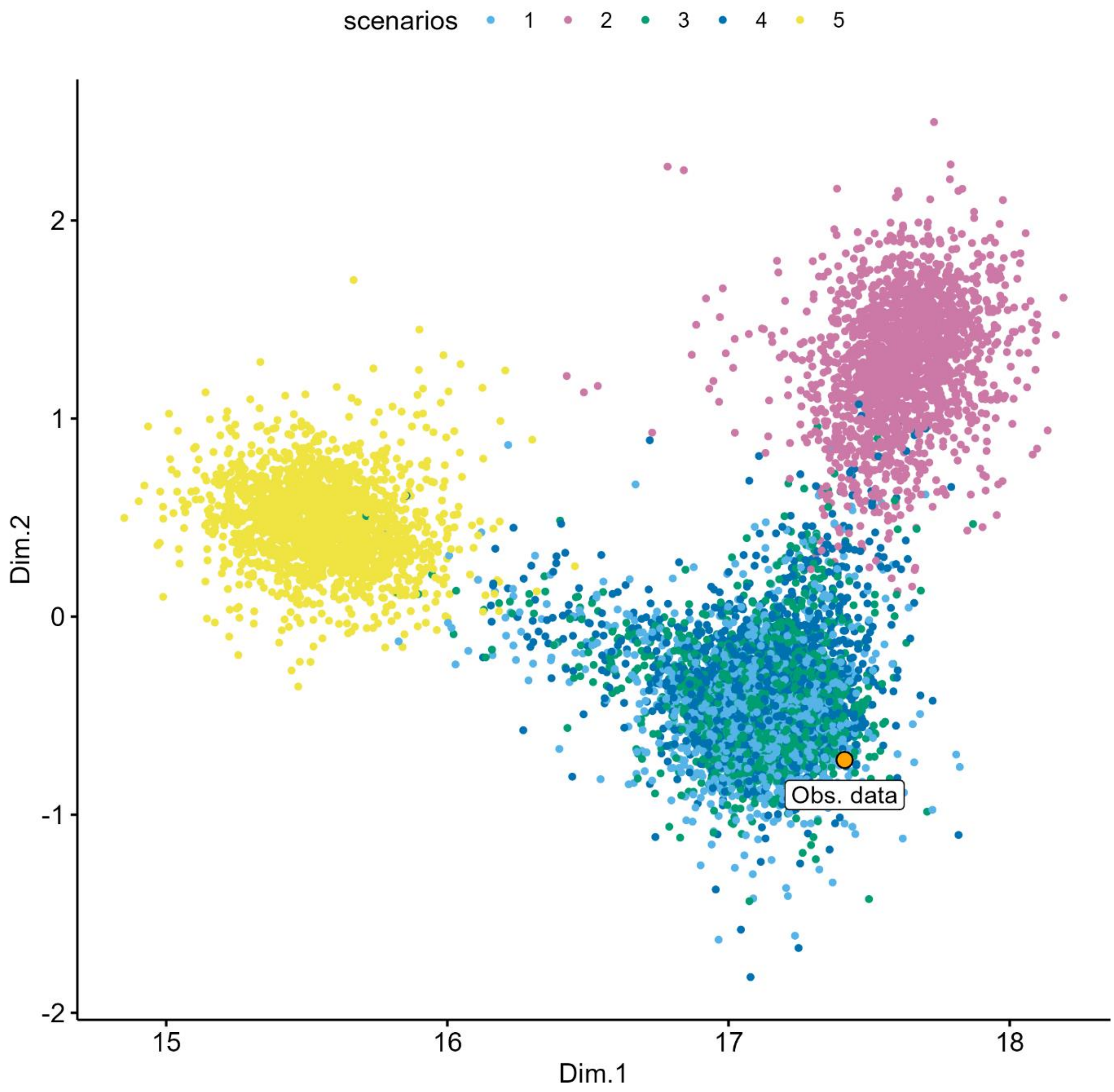

Figure S16. Projection of simulated datasets of global model (model set 21) on the first two axes of a Linear Discriminant Analysis. The location of the observed genetic data is indicated by the yellow circle. 16,500 datasets were used for model choice.

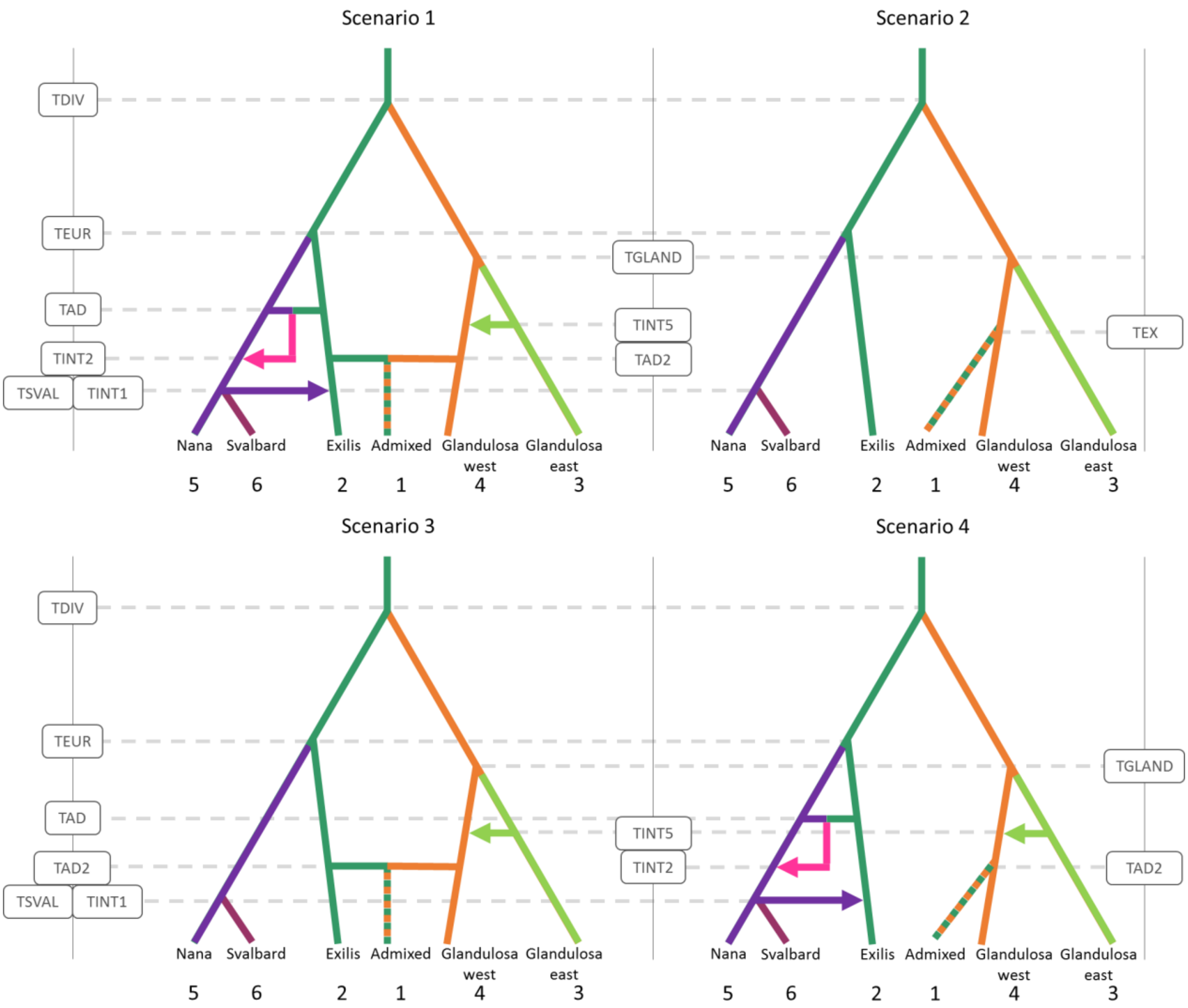

Figure S17. Global model (model set 22) with Exilis as ancestral population.

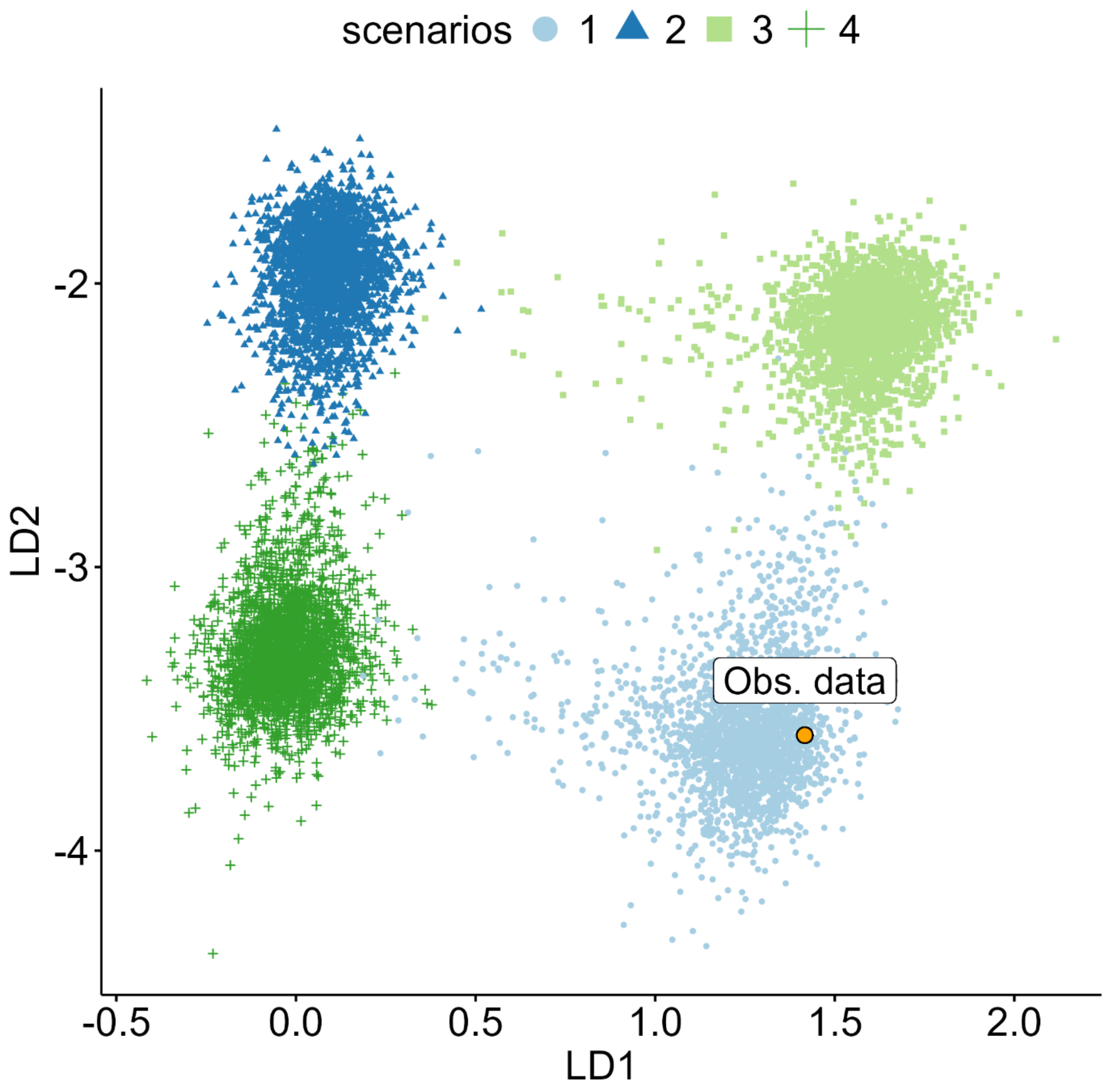

Figure S18. Projection of simulated datasets of global model (model set 22) on the first two axes of a Linear Discriminant Analysis. 100,000 datasets were used for model choice but only a random 10,000 subsample are plotted. The location of the observed genetic data is indicated by the yellow circle.

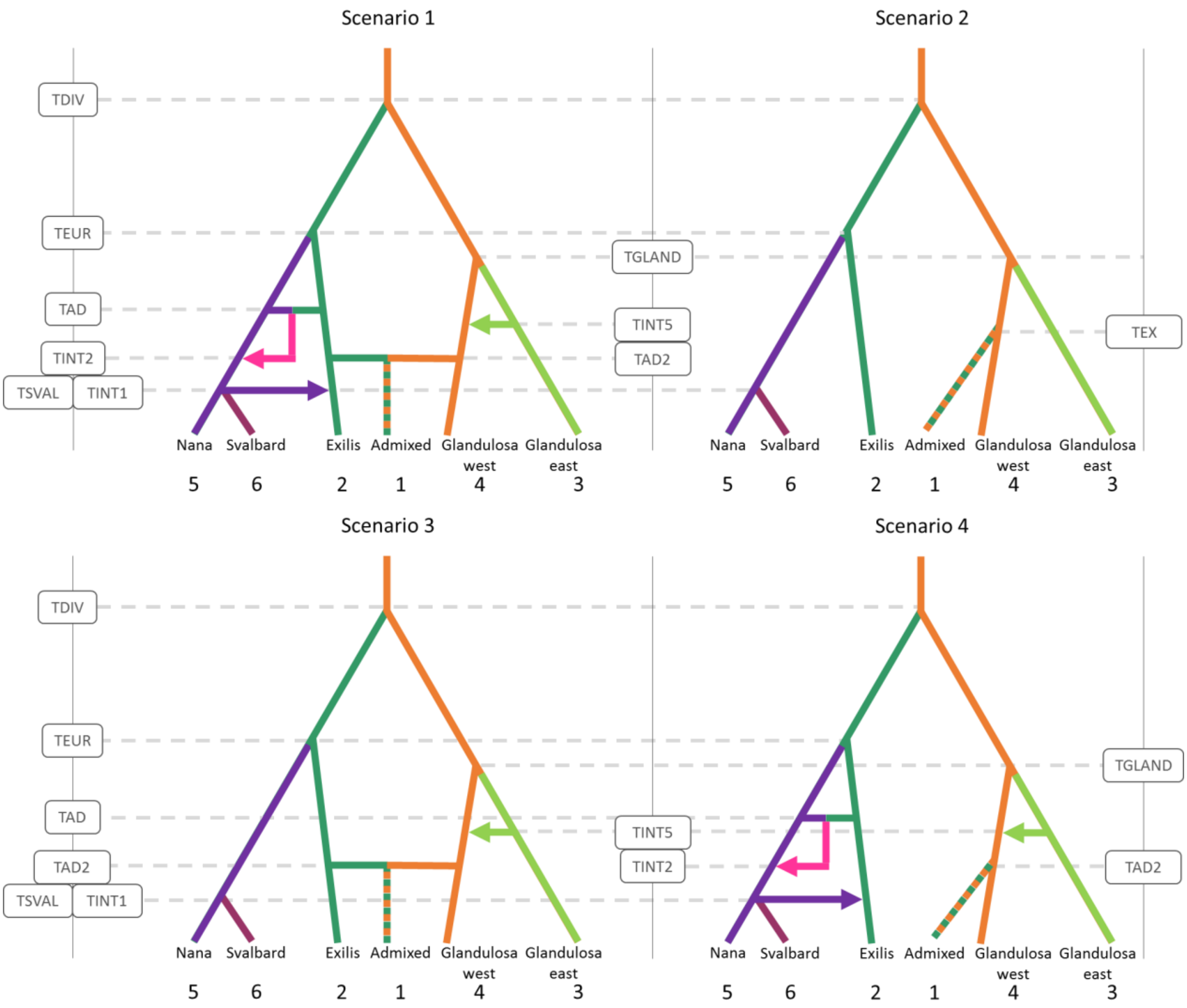

Figure S19. Global model (model set 23) with Glandulosa as ancestral population.

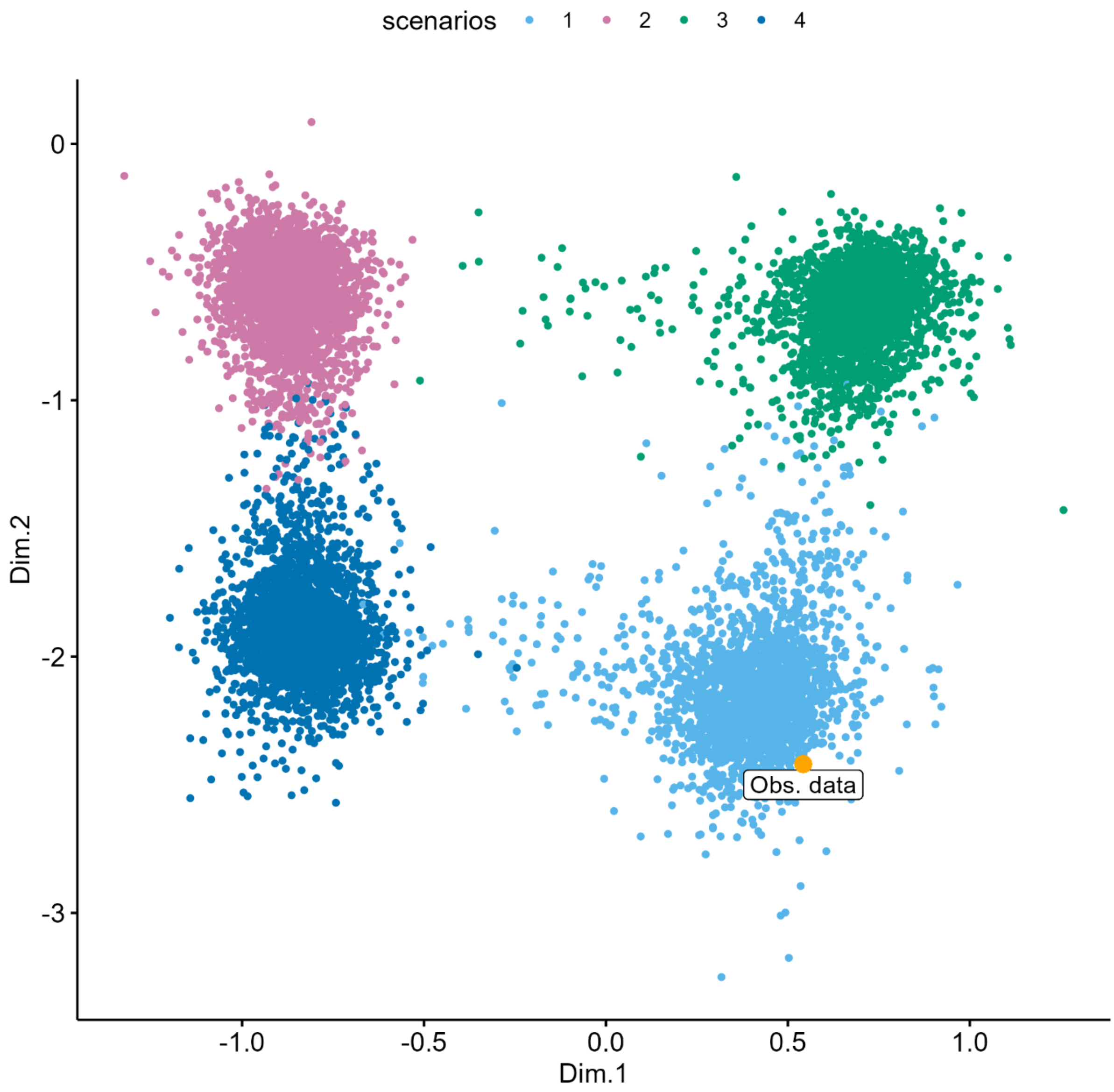

Figure S20. Projection of simulated datasets of global model (model set 23) on the first two axes of a Linear Discriminant Analysis. 100,000 datasets were used for model choice but only a random 10,000 subsample are plotted. The location of the observed genetic data is indicated by the yellow circle.

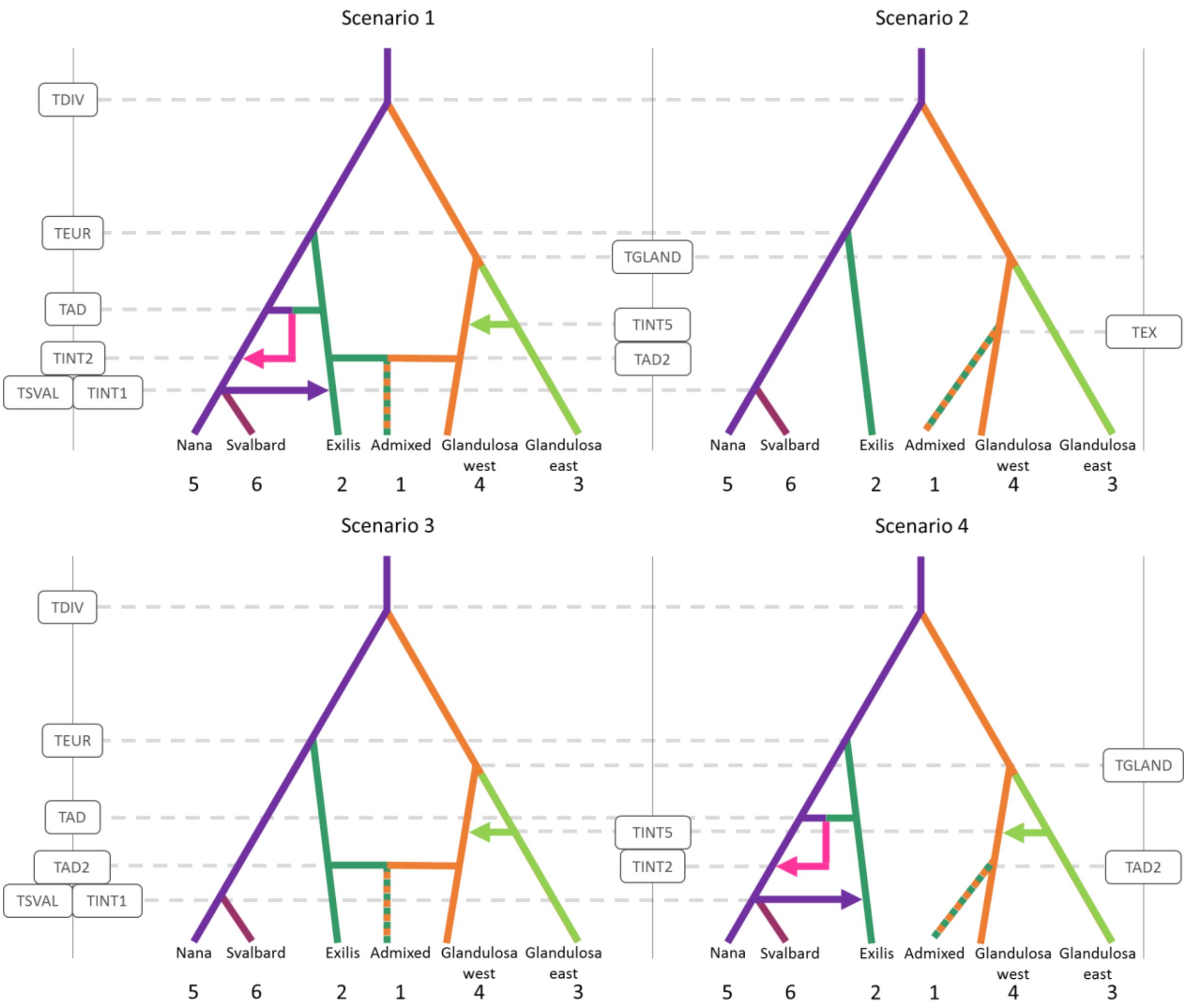

Figure S21. Global model (model set 24) with Nana as ancestral population.

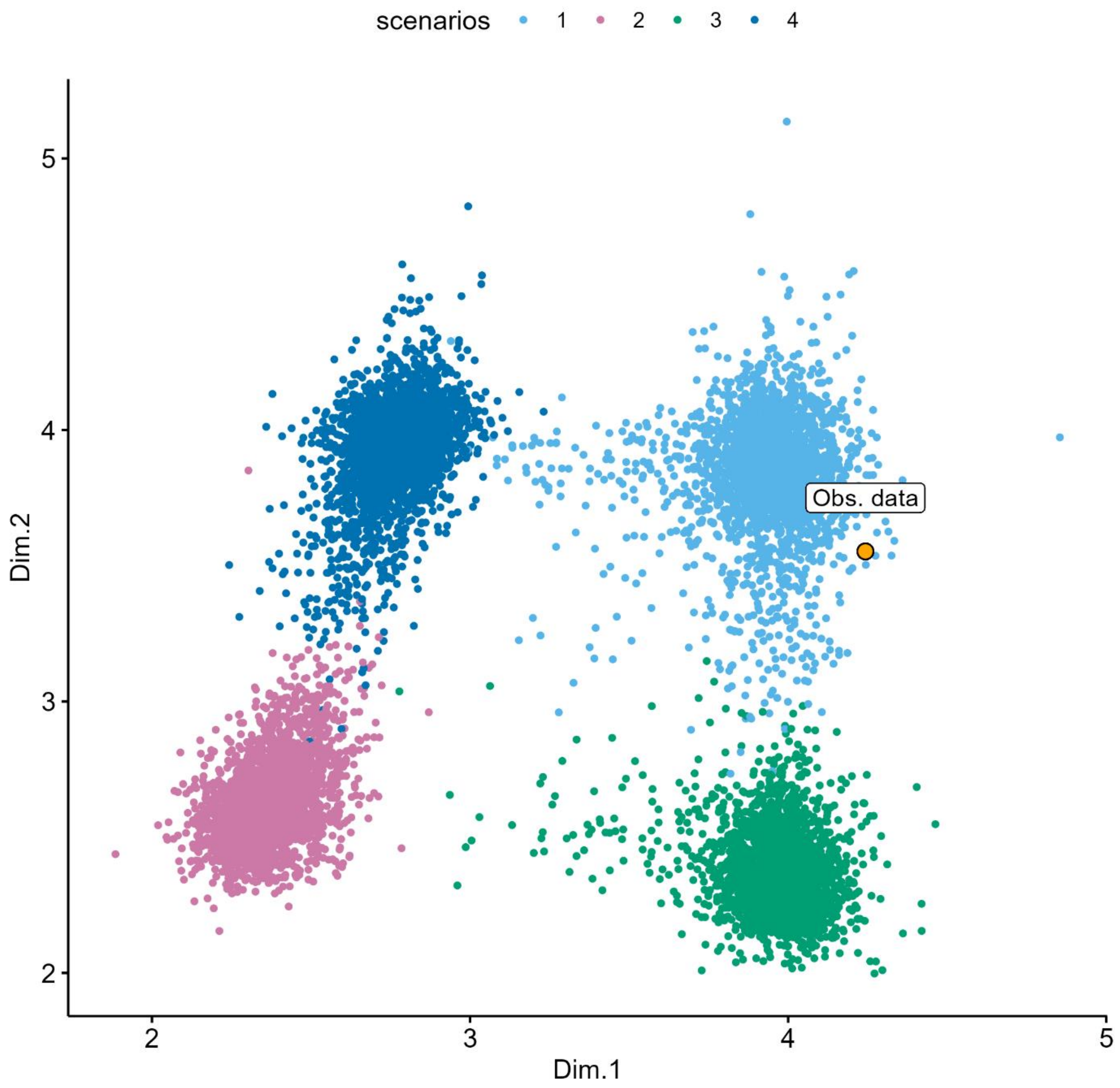

Figure S22. Projection of simulated datasets of global model (model set 24) on the first two axes of a Linear Discriminant Analysis. 100,000 datasets were used for model choice but only a random 10,000 subsample are plotted for clarity. The location of the observed genetic data is indicated by the yellow circle.

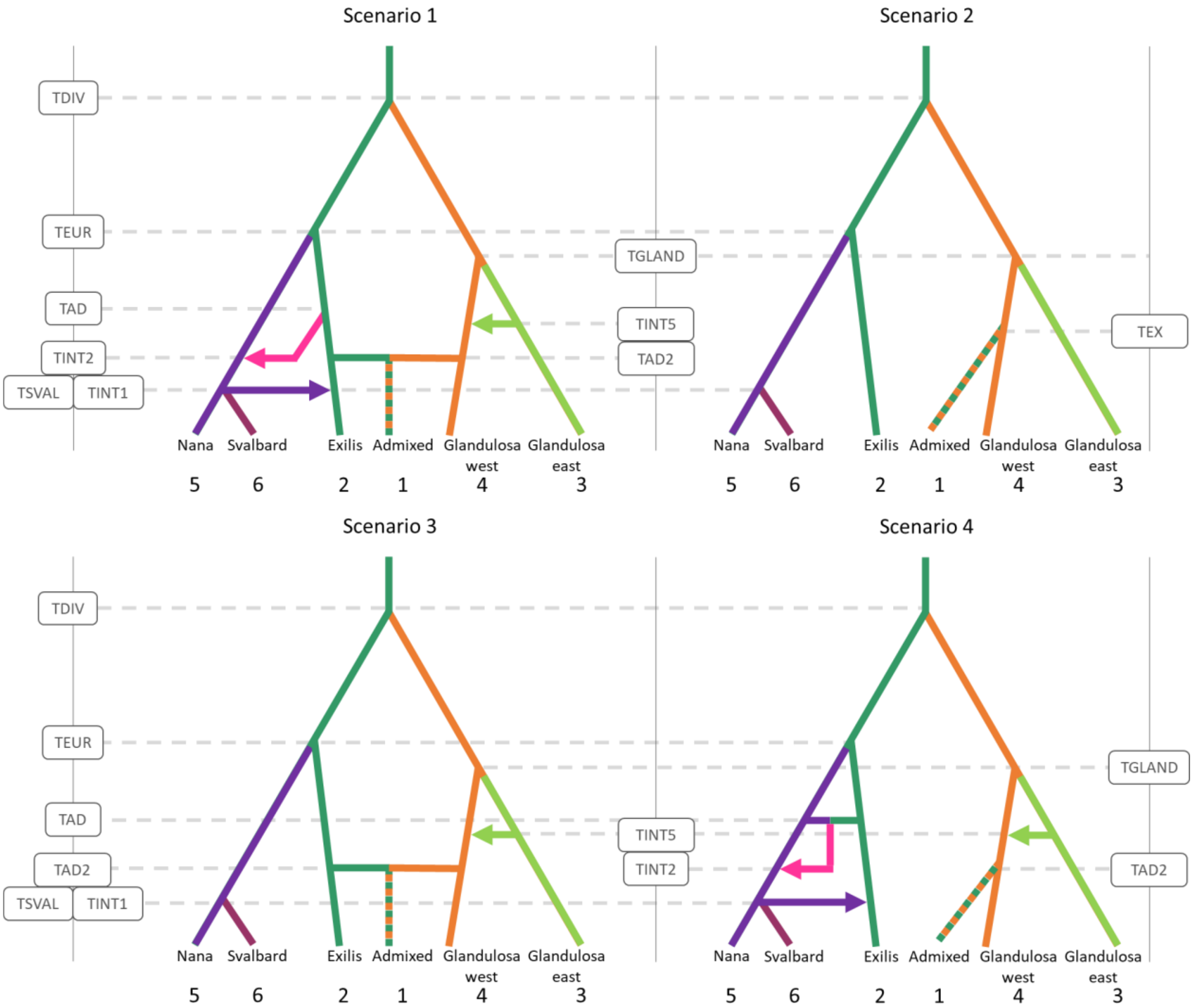

Figure S23. Global model (model set 24) with Exilis as ancestral population and Tundrarum formed from divergence from Exilis in scenario 1.

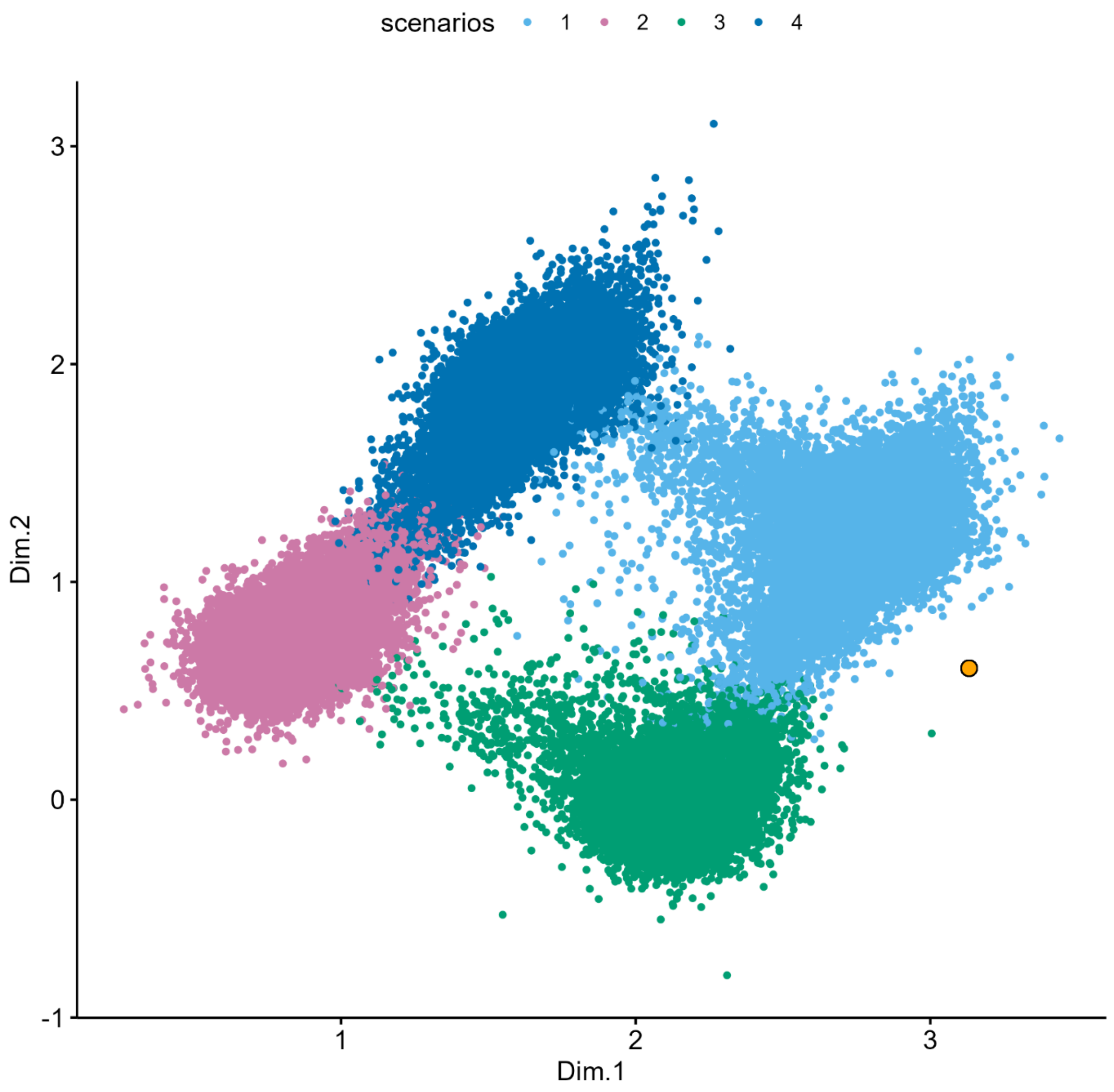

Figure S24. Projection of simulated datasets of global model (model set 25) on the first two axes of a Linear Discriminant Analysis. 100,000 datasets were used for model choice and are plotted. The location of the observed genetic data is indicated by the yellow circle.

Table S5. DIYABC RF (Collin et al., 2021) model specifications for the final global model (model set 22) used for parameter estimates. Model specifications form part of the DIYABC header input file.

Priors	Scenario 1	Scenario 2	Scenario 3	Scenario 4
**Population size**	N1 N UN[10000,150000,0,0]<br>N2 N UN[1000,75000,0,0]<br>N3 N UN[10000,60000,0,0]<br>N4 N UN[10000,150000,0,0]<br>N5 N UN[5000,100000,0,0]<br>N6 N UN[27,750,0,0]<br>N7 N UN[1000,100000,0,0]			
**Conditions**	TINT2>TINT1, TINT2>TSVAL, TAD2>TINT1, TAD>TSVAL, TAD>TINT2, TAD>TAD2, TEUR>TAD, TEUR>TINT1, TEUR>TSVAL, TEUR>TAD2, TINT5>TAD2, TGLAND>TINT5, TGLAND>TEX, TDIV>TEUR, TDIV>TGLAND			
**Locus description**	543 <A> G1 from 1			
**Topology**	scenario 1 [0.25] (21)<br>N1 N2 N3 N4 N5 N6 N2 N5 N7 N7 N4 N3 N5<br>0 sample 1<br>0 sample 2<br>0 sample 3<br>0 sample 4<br>0 sample 5<br>0 sample 6<br>TSVAL merge 5 6<br>TINT1 split 2 13 7 r1<br>TINT1 merge 5 13<br>TINT2 split 5 8 9 r2<br>TINT2 merge 10 9	scenario 2 [0.25] (11)<br>N1 N2 N3 N4 N5 N6<br>0 sample 1<br>0 sample 2<br>0 sample 3<br>0 sample 4<br>0 sample 5<br>0 sample 6<br>TSVAL merge 5 6<br>TEX merge 4 1<br>TEUR merge 2 5<br>TGLAND merge 4 3<br>TDIV merge 2 4	scenario 3 [0.25] (14)<br>N1 N2 N3 N4 N5 N6 N4 N3<br>0 sample 1<br>0 sample 2<br>0 sample 3<br>0 sample 4<br>0 sample 5<br>0 sample 6<br>TSVAL merge 5 6<br>TAD2 split 1 2 4 r3<br>TEUR merge 2 5<br>TINT5 split 4 8 7 r5<br>TINT5 merge 3 7<br>TGLAND merge 8 3	scenario 4 [0.25] (20)<br>N1 N2 N3 N4 N5 N6 N2 N5 N7 N7 N4 N3 N5<br>0 sample 1<br>0 sample 2<br>0 sample 3<br>0 sample 4<br>0 sample 5<br>0 sample 6<br>TSVAL merge 5 6<br>TINT1 split 2 13 7 r1<br>TINT1 merge 5 13<br>TAD2 merge 4 1<br>TINT2 split 5 8 9 r2

	TAD2 split 1 7 4 r3<br>TAD split 10 8 7 r4<br>TEUR merge 7 8<br>TINT5 split 4 11 12 r5<br>TINT5 merge 3 12<br>TGLAND merge 11 3<br>TDIV merge 7 11		TDIV merge 2 8	TINT2 merge 10 9<br>TAD split 10 8 7 r4<br>TEUR merge 7 8<br>TINT5 split 4 11 12 r5<br>TINT5 merge 3 12<br>TGLAND merge 11 3<br>TDIV merge 7 11
**Admixture priors**	r1 A UN[0.01,0.99,0,0]<br>r2 A UN[0.01,0.99,0,0]<br>r3 A UN[0.01,0.99,0,0]<br>r4 A UN[0.01,0.99,0,0]<br>r5 A UN[0.01,0.99,0,0]			
**Event parameter priors**	TSVAL T UN[10,2000,0,0]<br>TINT1 T UN[10,2400,0,0]<br>TINT2 T UN[10,2400,0,0]<br>TAD2 T UN[10,9000,0,0]<br>TAD T UN[9000,24000,0,0]<br>TEUR T UN[25000,100000,0,0]<br>TINT5 T UN[100,20000,0,0]<br>TGLAND T UN[10000,90000,0,0]<br>TDIV T UN[80000,200000,0,0]<br>TEX T UN[10,10000,0,0]			

Table S6. Model error rates for DIYABC RF (Collin et al., 2021) model sets.

Model set	Prior error rate			Model 1	Model 2	Model 3	Model 4		Class error
Species divergence: 4.3	0.0371	predicted	model 1	2249	112	0	0		0.0474
		predicted	model 2	258	2414	0	0		0.0966
		predicted	model 3	0	0	2496	1		0.0004
		predicted	model 4	0	0	0	2470		0
				model 1	model 2	model 3			class error
Eurasia without Svalbard: 8.5	Prior error rate	predicted	model 1	2875	226	151			0.116
	0.2838	predicted	model 2	274	2129	938			0.363
		predicted	model 3	267	982	2158			0.367
Eurasia with Svalbard: 9				model 1	model 2	model 3			class error
	Prior error rate	predicted	model 1	3327	9	12			0.006272
	0.3299	predicted	model 2	20	1640	1586			0.495
		predicted	model 3	8	1664	1734			0.491
Eurasia with Svalbard 10.1	Prior error rate			model 1	model 2				class error
	0.0982	predicted	model 1	4277	297				0.0649
		predicted	model 2	685	4741				0.126
North America: 14.1	Prior error rate			model 1	model 2	model 3			class error
	0.003	predicted	model 1	3315	1	1			0.0006
		predicted	model 2	12	3343	0			0.00358
		predicted	model 3	16	0	3312			0.00481

Global model: 20	Prior error rate			model 1	model 2	model 3			class error
	0.0001	predicted	model 1	3244	0	1			0.00031
		predicted	model 2	0	3356	0			0
		predicted	model 3	0	0	3399			0
Global model: 21	Prior error rate			model 1	model 2	model 3	model 4	model 5	class error
	0.193333	predicted	model 1	1886	5	810	176	0	0.345
		predicted	model 2	23	3243	29	67	1	0.0357
		predicted	model 3	948	9	2109	313	0	0.376
		predicted	model 4	373	0	344	2753	0	0.207
		predicted	model 5	38	5	25	24	3319	0.027
Global model: 22	Prior error rate			model 1	model 2	model 3	model 4		class error
	0.00406	predicted	model 1	24698	0	39	1		0.00162
		predicted	model 2	0	25011	24	78		0.00406
		predicted	model 3	115	0	25042	0		0.00457
		predicted	model 4	104	45	0	24843		0.00596
Global model: 23	Prior error rate			model 1	model 2	model 3	model 4		class error
	0.00432	predicted	model 1	24743	0	27	5		0.00162
		predicted	model 2	0	24921	22	99		0.00406
		predicted	model 3	107	0	24987	0		0.00457
		predicted	model 4	107	64	1	24917		0.00596
Global model: 24	Prior error rate			model 1	model 2	model 3	model 4		class error
	0.0039	predicted	model 1	24617	0	27	4		0.00126
		predicted	model 2	0	24919	37	77		0.00455
		predicted	model 3	98	1	24845	0		0.00397

		predicted	model 4	92	51	3	25229		0.00575
Global model: 25	Prior error rate	predicted	model 1	model 1	model 2	model 3	model 4		Class error
	0.00436	predicted	model 2	24565	1	40	0		0.00167
		predicted	model 3	1	24798	39	79		0.00478
		predicted	model 4	74	3	25280	0		0.00304
				133	56	10	24921		0.008

Table S7. Posterior probabilities of DIYABC RF (Collin et al., 2021) model sets. Model choice conducted with 10,000-100,000 datasets depending on the model, 500 trees, minimum node size of 1, n summary statistics, n axes of summary statistics LDA linear combination (1 - number of scenarios), and 5 noise variables.

Model set	Votes model 1	Votes model 2	Votes model 3	Votes model 4	Votes model 5	Selected model	Posterior probability
Species divergence: 4.3	186	180	110	24	NA	1	0.77
Eurasia without Svalbard: 8.5	480	15	5	NA	NA	1	0.994
Eurasia with Svalbard: 9	162	114	224	NA	NA	3	0.541
Eurasia with Svalbard: 10.1	245	255	NA	NA	NA	2	0.772
North America: 14.1	476	8	16	NA	NA	1	0.98
Global model: 20	9	24	467	NA	NA	3	1
Global model: 21	262	11	169	58	0	1	0.55
Global model: 22	439	0	53	8	NA	1	0.891
Global model: 23	449	0	48	3	NA	1	0.869
Global model: 24	457	0	41	2	NA	1	0.896
Global model: 25	906[1]	0	91	3	NA	1	0.925

[1] 1000 trees were used in this Random Forest Classification.

Appendix S7: DIYABC RF parameter estimation and generation time

Inferring generation time is challenging in woody plant species such as dwarf birch species due to their longevity, vegetative reproduction, generational overlap, and variable age of maturity depending on environmental conditions (Tsuda et al., 2017). An average generation time was approximated using the colonisation date of first occurrence of *Betula* pollen in Scotland inferred from the pollen record from the Neotoma database (Bartley, 2019; Bartley & Morgan, 1990; Fyfe et al., 2009; Giesecke et al., 2014; Williams et al., 2018), which was divided by the number of generations estimated for a Scandinavian-British population divergence in a previous study of *B. nana* demographic history using RADseq data (Borrell et al., 2018).

Calculating generation time using pollen records could be affected by lower-resolution time scales and uncertainties in colonisation timing due to long-distance dispersal of pollen (Parducci et al., 2015). However, there are also uncertainties in estimating generation time directly from observations because of vegetative growth and reduced sexual reproduction in unfavourable environments (de Groot, 1997), leading to difficulties in estimating age of first seed reproduction, i.e. generation time. For example, based on ring-width analysis, one study in northeast Siberia found that dwarf birch ramets (clones/individuals belonging to the same genet, or genetic parent individual) were typically established 20 - 50 years ago (Li et al., 2015), while another study found an estimated average/maximum plant age of 60/160 years in East Greenland (Büntgen et al., 2015). Genet turnover is likely also slower than mean ramet turnover, leading to a potentially much longer generation time.

Appendix S8: Population genetic structure and diversity

Table S8. Genetic diversity of dwarf birch sampling sites. Gene diversity (Expected heterozygosity) was calculated using 7232 variant sites whereas rarefied allelic richness which was calculated using 1724 variant sites. Mean global gene diversity was 0.091 (SD 0.0196) and mean rarefied allelic richness was and 1.10 (SD 0.0177).

Sampling site ID	Locality	Number of individuals	Gene diversity (He)	Rarefied Allelic Richness (Ar)
BN01	Itkillik	3.89	0.116	1.133
BN02	Chandalar	5.89	0.095	1.1
BN03	Dalton Mountain	5.82	0.104	1.112
BN04	Kuparuk River	6.74	0.122	1.134
BN05	Roche Mountonee	5.9	0.087	1.089
BN06	Bunny Haven	5.9	0.101	1.108
BN07	Komaktorvik River	5.88	0.081	1.08
BN08	Kytalyk1	5.85	0.103	1.107
BN09	Kytalyk2	5.82	0.105	1.107
BN10	Hol	5.88	0.091	1.102
BN12	Atqasuk	3.83	0.107	1.128
BN13	Torngats Basecamp	5.89	0.075	1.076
BN14	Nain	5.85	0.079	1.079
BN15	Näkkälä	4.89	0.097	1.106
BN16	Nordkapp	5.83	0.097	1.105
BN17	Saariselkä	5.85	0.097	1.102
BN21	East Fork Chandalar River	5.9	0.083	1.088
BN22	Linje	3.94	0.078	1.084
BN23	Nome	4.8	0.098	1.107
BN24	Kuzitin	5.82	0.126	1.139
BN25	Old Man	4.88	0.103	1.116
BN29	SAG	3	0.103	1.126
BN30	Fox	5.84	0.119	1.13
BN32	Krutaya Drisva	5.87	0.104	1.109
BN33	Ambarchik	5.88	0.097	1.104
BN34	Healy	5.78	0.119	1.134
BN37	Endalen & Janssonhaugen	6.81	0.079	1.082
BN38	Kvaløya	5.84	0.096	1.102
BN39	Vadsø	4.84	0.092	1.099
BN40	St. Mary's	5.84	0.089	1.1
BN42	Kilpisjärvi	5.9	0.096	1.102
BN43	Joensuu	5.91	0.085	1.091
BN44	Lifjeld	2	0.067	1.084
BN45	Les Ponts-de-Martel	5.91	0.09	1.101
BN46	Og Lake	5.93	0.064	1.068
BN49	Kluane - Pika Valley	4.9	0.08	1.084
BN51	QHI - Hawk Valley	5.9	0.091	1.099

BN61	Erkuta Tundra Monitoring site	3	0.092	1.111
BN62	Malozemelskaya tundra. Nenetskaya Gryada, Korovinskaya Guba; 5.5-8 km W Cape Seduysky Nos, c. 0-3 km W to NW the shore	3	0.084	1.098
BN63	Ary-Mas nature reserve, (c. 50-60 km NNW Khatanga), right riverside of Novaya, along the river and up to c. 3 km S of river	1	0.05	1.104
BN64	Lena R. west bank: Chekurovka village, surroundings of settlement	3	0.082	1.092
BN67	5-10 km from Arctic Station (coordinates are for station)	1	0.039	1.078
BN68	South of the landingstrip close to "Kjovedammen" and "Teltdammen".	1	0.034	1.074

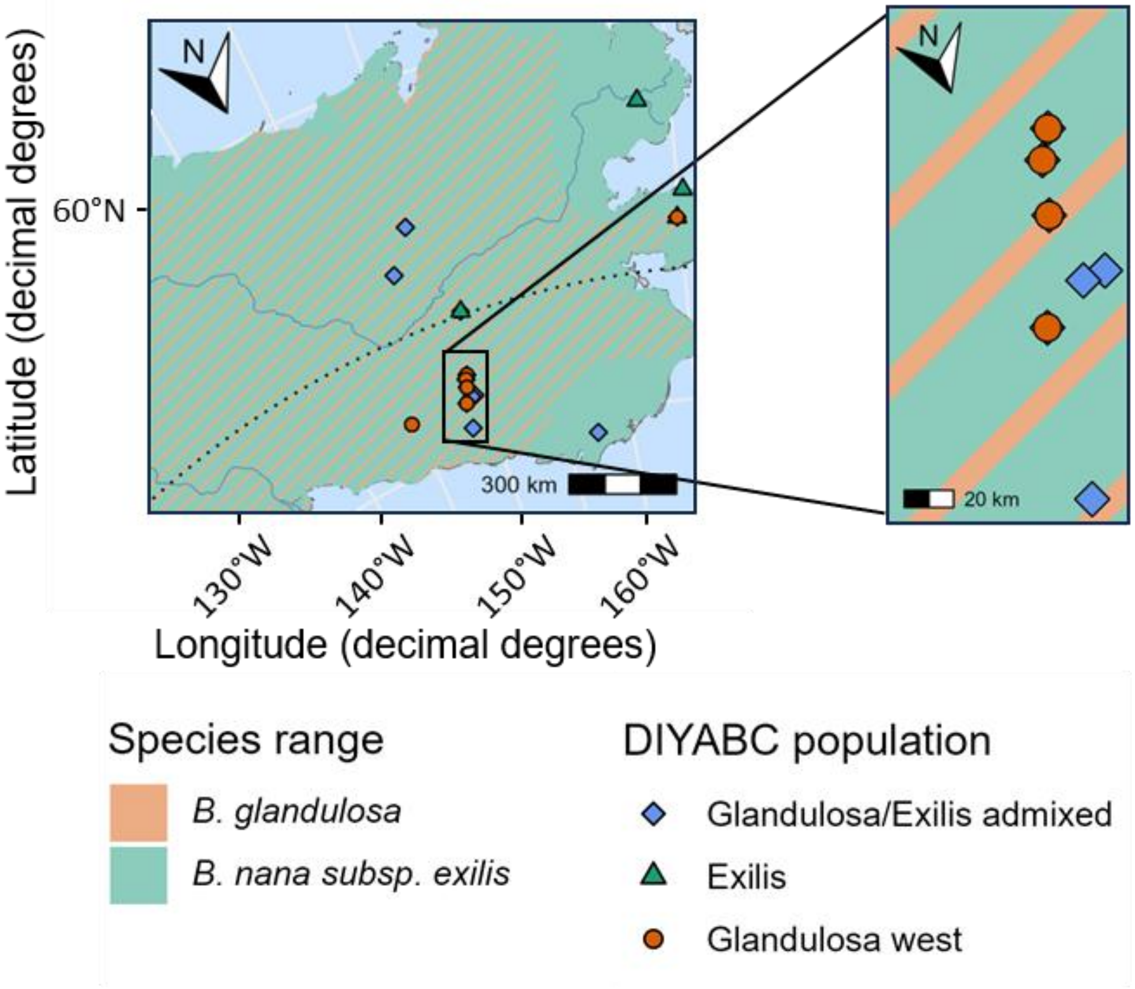

Figure S25. Alaskan dwarf birch sampling sites. Sites coloured according to populations used in Approximate Bayesian computation (ABC) modelling with DIYABC RF (Collin et al., 2021). Populations were assigned using ancestral group coefficients from ADMIXTURE analysis.

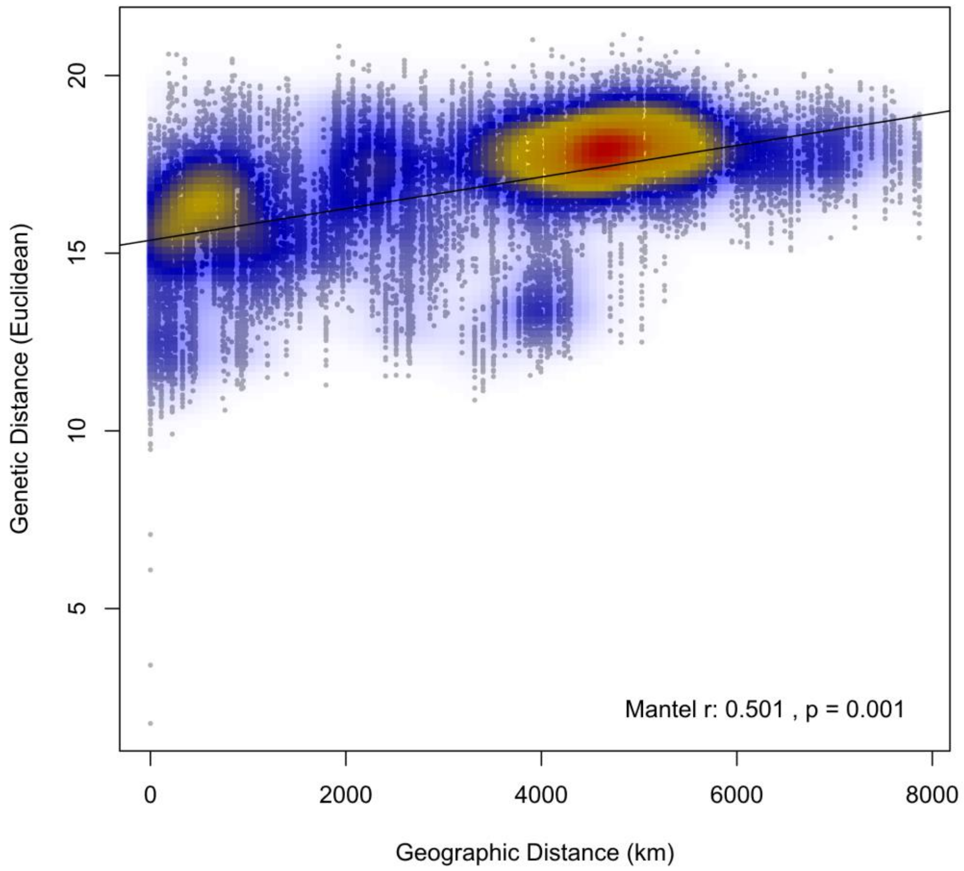

Figure S26. The panarctic relationship between geographic and genetic distances in dwarf birch individuals. Isolation by distance is indicated by the significant Mantel statistic (bottom right). A wide, variable band of points along the regression line (R^2=0.251, p=0.001) suggests variability in the relationship between genetic and geographic distances, potentially due to migration or admixture. A kernel density estimate (KDE) heatmap is plotted to illustrate the density and distribution of pairwise genetic and geographic comparisons.

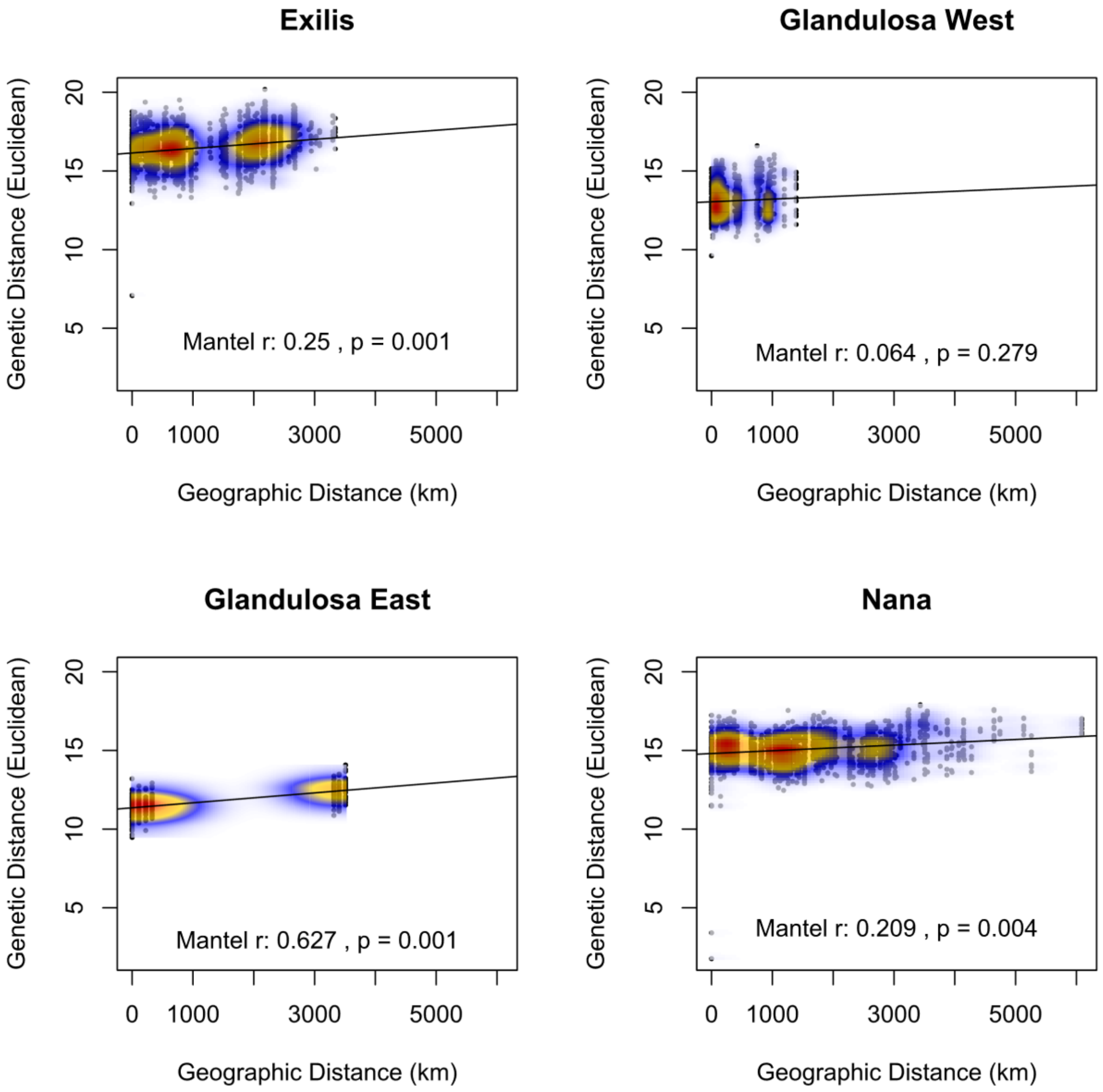

Figure S27. The relationship between geographic and genetic distances in dwarf birch individuals by genetic group. Isolation by distance is indicated by the significant Mantel statistic (bottom right). A kernel density estimate (KDE) heatmap is plotted to illustrate the density and distribution of pairwise genetic and geographic comparisons. The Tundraraum group was not tested due to a small number of data points.

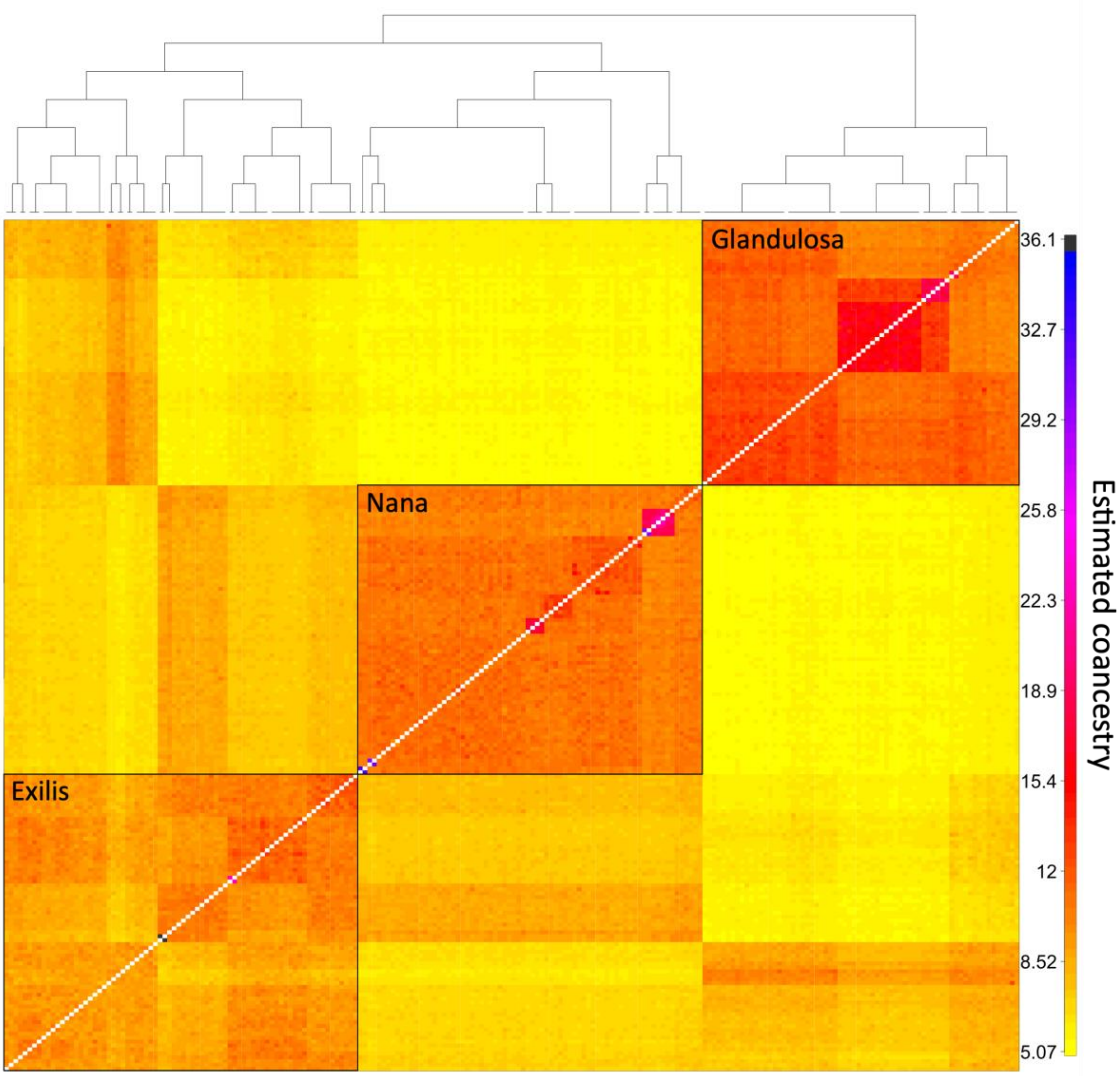

Figure S28. Global dwarf birch population genetic structure inferred from RADPainter. Coancestry matrix summarising nearest neighbour haplotype relationships between 218 individuals inferred from linkage structure derived from 3,830 SNPS. Degree of coancestry is indicated by the colour scale. The matrix is clustered according to fineRADstructure population relationships identified using a Markov Chain Monte Carlo (MCMC) clustering algorithm (dendrogram). Discrete blocks of high coancestry represent a population where individuals are genetically indistinguishable from each other and different from other populations, while interpopulation coancestry indicates interpopulation gene flow and admixture (Malinsky et al., 2018). Regional RADPainter analyses were also conducted (**Figures S3, S4**). Proposed genetic groups are marked with Exilis, Nana, and Glandulosa.

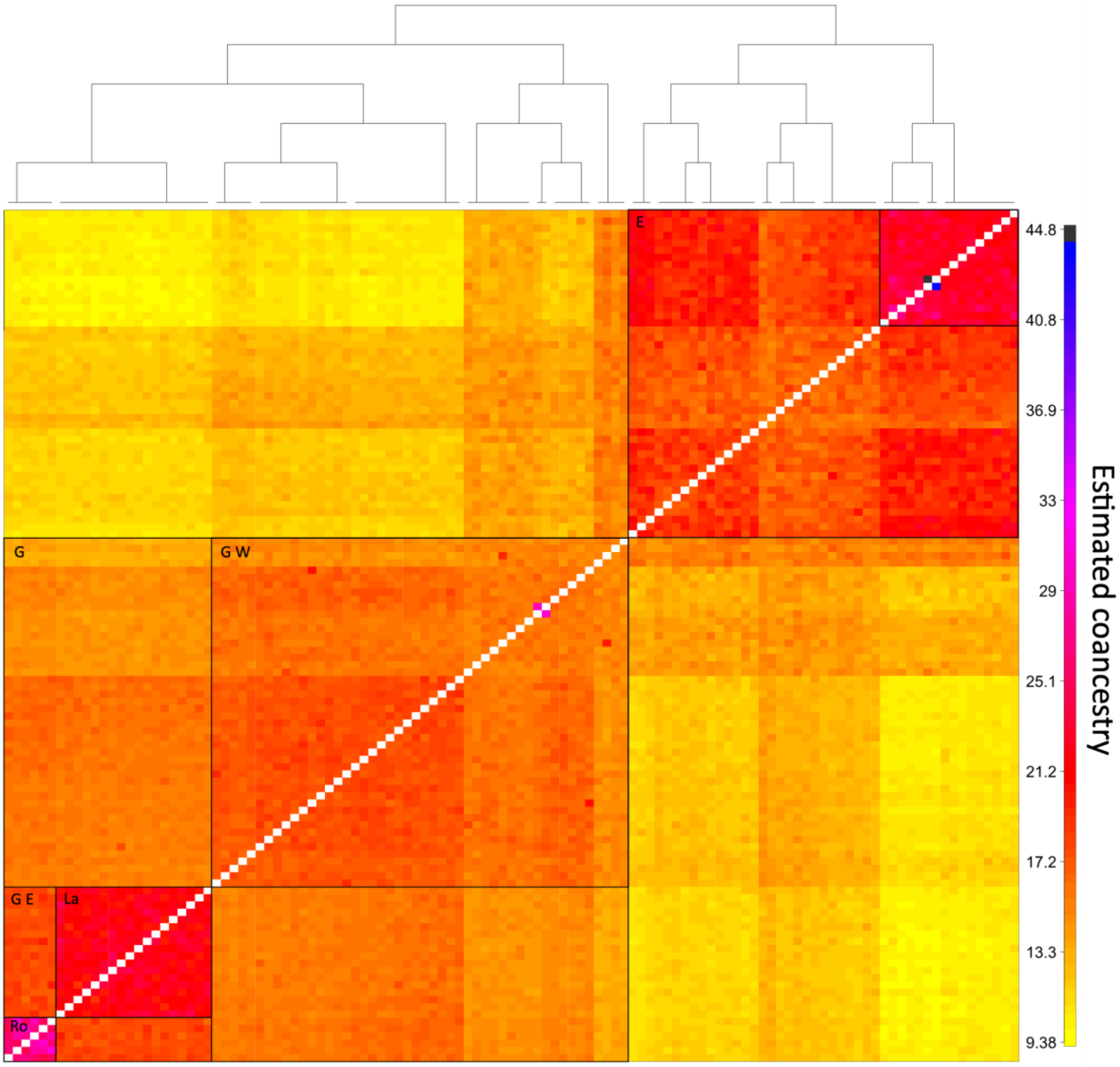

Figure S29. North American dwarf birch population genetic structure inferred from RADPainter. Coancestry matrix summarising nearest neighbour haplotype relationships between 117 individuals inferred from linkage structure derived from 2698 SNPS. Degree of coancestry is indicated by the colour scale. The matrix is clustered according to fineRADstructure population relationships using a Markov chain Monte Carlo (MCMC) clustering algorithm (dendrogram). The presence of discrete blocks of higher coancestry represents a genetic population where individuals are genetically indistinguishable from each other and different from other populations, while higher coancestry between different populations indicates gene flow and admixture between populations (Malinsky et al., 2018). Letters represent possible populations: E = Exilis, G = Glandulosa, GW = Glandulosa west, GE = Glandulosa east, La= Labrador, Ro = Rocky mountains.

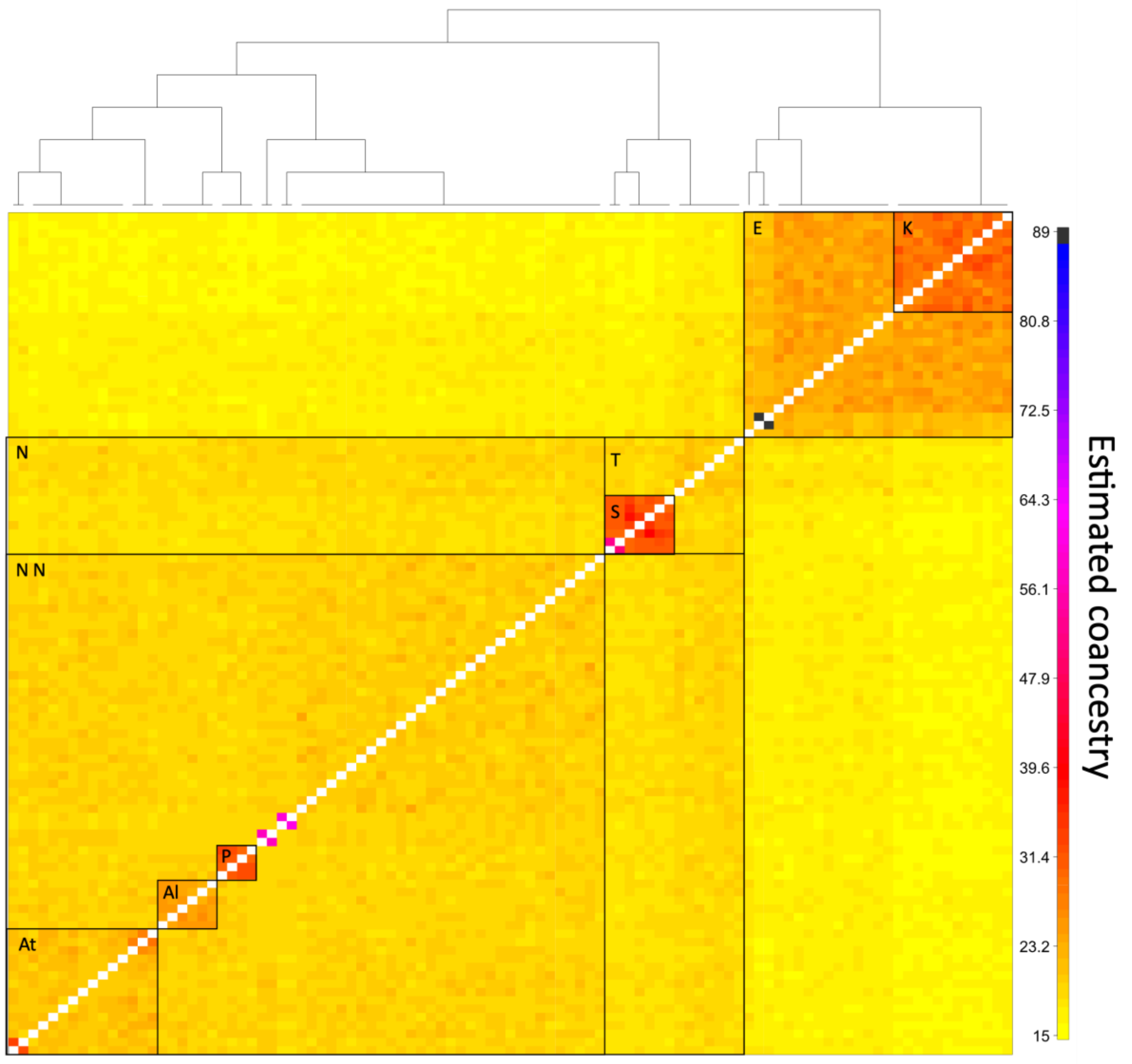

Figure S30. **Eurasian dwarf birch population genetic structure inferred from RADPainter.** Coancestry matrix summarising nearest neighbour haplotype relationships between 101 individuals inferred from linkage structure derived from 3624 SNPS. Degree of coancestry is indicated by the colour scale. The matrix is clustered according to fineRADstructure population relationships using a Markov chain Monte Carlo (MCMC) clustering algorithm (dendrogram). The presence of discrete blocks of higher coancestry represents a genetic population where individuals are genetically indistinguishable from each other and different from other populations, while higher coancestry between different populations indicates gene flow and admixture between populations (Malinsky et al., 2018). Letters represent possible populations: N = Nana, E = Exilis, NN = Nana subsp. nana, K = Kolyma River, T = Tundrarum, S = Svalbard, At = Atlantic, Al = Alps, P = Poland.

Appendix S9: DIYABC RF parameter estimates

Table S9. Time parameter estimates for the best supported scenario in global model set 22. Estimates were based on 24,917 simulated datasets from scenario 1, with minimum node size of 5, 572 summary statistics, n axes of summary statistics in PLS linear combination, 5 noise variables, and with 1000 out-of-band samples used as a test. Estimates are given in generations (gens) and years with (a) 10-year generation time and (b) 14-year generation time. 95% confidence intervals and local posterior root-mean-square error (RMSE) have the same units as the variable, and local posterior Normalised RMSE (RMSE divided by the median parameter estimate) is unitless.

Event	Event type	Description	Potential Region	Median gens	Years (10)	95% CI		Years (14)	95% CI		RMSE	NRMSE
TSVAL	divergence	Svalbard and Nana	Barents Sea Islands	444	4,440	908	7,040	6,216	1,272	9,856	192.756	0.434
TINT1	introgression	Nana to Exilis	Siberia / Lena River	923	9,230	1,430	19,960	12,922	2,002	27,944	481.909	0.522
TINT2	introgression	Tundrarum to Nana	Northwest Siberia	1,544	15,440	6,060	23,170	21,616	8,484	32,438	493.066	0.319
TAD2	admixture	Exilis and Glandulosa west	Beringia	2,959	29,590	6,474	65,150	41,426	9,064	91,210	1227.015	0.415
TINT5	introgression	Glandulosa east to Glandulosa west	North America	6,996	69,960	28,215	151,450	97,944	39,501	212,030	2464.674	0.352

TAD	admixture	Nana and Exilis	Siberia / Lena River	15,486	154,860	97,133	231160	216,804	135,987	323,624	4540.784	0.293
TGLAND	divergence	Glandulosa east and Glandulosa west	North America	50,292	502,921	157,700	856,896	704,089	220,780	1,199,654	22295.23	0.443
TEUR	divergence	Nana and Exilis	Eurasia	58,837	588,367	278,050	937,323	823,714	389,270	1,312,252	20703.55	0.352
TDIV	divergence	Glandulosa and Exilis	Beringia / North America	89,750	897,497	806,395	1,145,920	1,256,496	1,128,953	1,604,288	10689.46	0.119

Table S10. Population size parameter estimates for the best supported scenario in global model set 22. Effective haploid population size (Ne) is 1/2 of effective population size as *Betula nana* and *Betula glandulosa* are diploid. 95% confidence intervals and local posterior root-mean-square error (RMSE) have the same units as the variable, and local posterior Normalised RMSE (RMSE divided by the median parameter estimate) is unitless.

Parameter	Population	Median (haploid)	95% CI (haploid)		Median (diploid)	95% CI (diploid)		RMSE	NRMSE
N1	Admixed	99461	30759	147742	198922	61518	295484	35543.650	0.357
N2	Exilis	59243	31197	73615	118486	62395	147229	9446.427	0.160
N3	Glandulosa east	47584	26518	59346	95167	53037	118691	6147.911	0.129
N4	Glandulosa west	104968	54350	145876	209936	108700	291752	25627.353	0.244
N5	Nana	71105	33705	97611	142210	67409	195223	17051.889	0.240
N6	Svalbard	503	93	726	1006	186	1452	235.398	0.470

N7	Tundrarum	65395	21738	97806	130789	43476	195612	24533.653	0.375

Table S11. Ratio of time/population size parameter estimates for the best supported scenario in global model set 22. Estimates were based on 24,917 simulated datasets from scenario 1, with minimum node size of 5, 572 summary statistics, n axes of summary statistics in PLS linear combination, 5 noise variables, and with 1000 out-of-band samples used as a test. 95% confidence intervals and local posterior root-mean-square error (RMSE) have the same units as the variable, and local posterior Normalised RMSE (RMSE divided by the median parameter estimate) is unitless.

Event	Event type	Description	Median estimate	95% CI		RMSE	NRMSE
TSVAL/N6	divergence	Svalbard and Nana	0.786	0.605	1.03	0.120	0.15248
TINT1/N2	introgression	Nana to Exilis	0.0195	0.00221	0.0688	0.00836	0.42983
TINT2/N5	introgression	Tundrarum to Nana	0.0349	0.0126	0.215	0.0780	2.23640
TAD2/N1	admixture	Exilis and Glandulosa west	0.0268	0.00721	0.0731	0.0136	0.50861
TINT5/N4	introgression	Glandulosa east to Glandulosa west	0.0888	0.0304	0.251	0.0470	0.52887
TAD/N7	admixture	Nana and Exilis	0.355	0.136	6.09	1.145	3.22868
TGLAND/N3	divergence	Glandulosa east and Glandulosa west	0.972	0.283	2.23	0.558	0.57346
TEUR/N5	divergence	Nana and Exilis	1.153	0.441	5.086	1.457	1.26338

TDIV/N4	divergence	Glandulosa and Exilis	0.867	0.594	1.604	0.113	0.13039

Table S12. Admixture rate parameter estimates for the best supported scenario in global model set 22. 95% confidence intervals and local posterior root-mean-square error (RMSE) have the same units as the variable, and local posterior Normalised RMSE (RMSE divided by the median parameter estimate) is unitless.

Parameter	Description	Median	95% CI		RMSE	NRMSE
r1	Proportion of Exilis genes from Nana	0.159	0.0414	0.386	0.178	1.12
r2	Proportion of Nana genes from Tundrarum	0.584	0.0772	0.922	0.297	0.509
r3	Proportion of Glandulosa genes from Exilis	0.512	0.363	0.640	0.0225	0.0439
r4	Proportion of Tundrarum genes from Nana	0.463	0.0397	0.843	0.297	0.641
1-r5	Proportion of Glandulosa west genes from Glandulosa west (as opposed to Glandulosa east)	0.663	0.0958	0.960	0.0347	0.0523

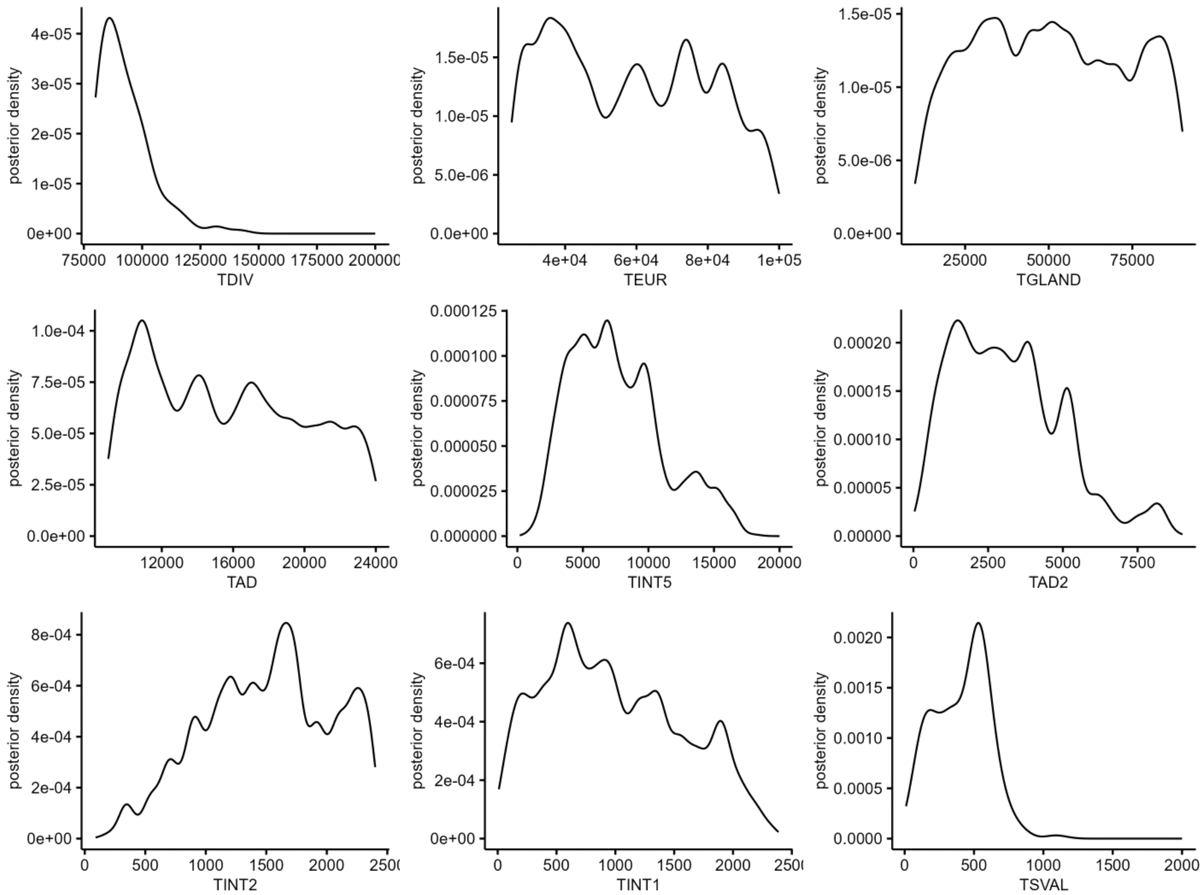

Figure S31. Posterior probability distributions for time parameters from the best supported scenario in global model set 22. Time given in number of generations.

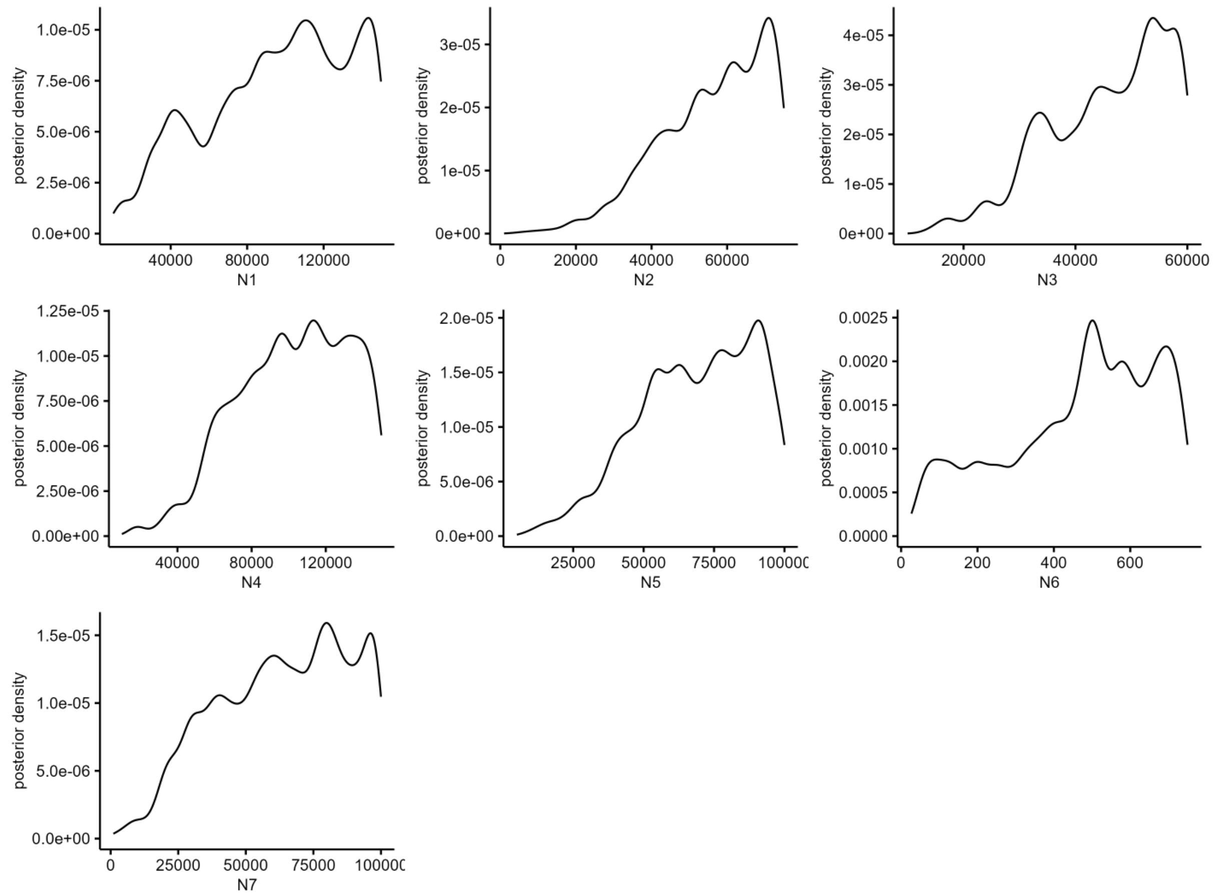

Figure S32. Posterior probability distributions for population size parameters from the best supported scenario in global model set 22. The parameters are effective haploid population size (Ne): 1/2 of effective population size as *Betula nana* and *Betula glandulosa* are diploid.

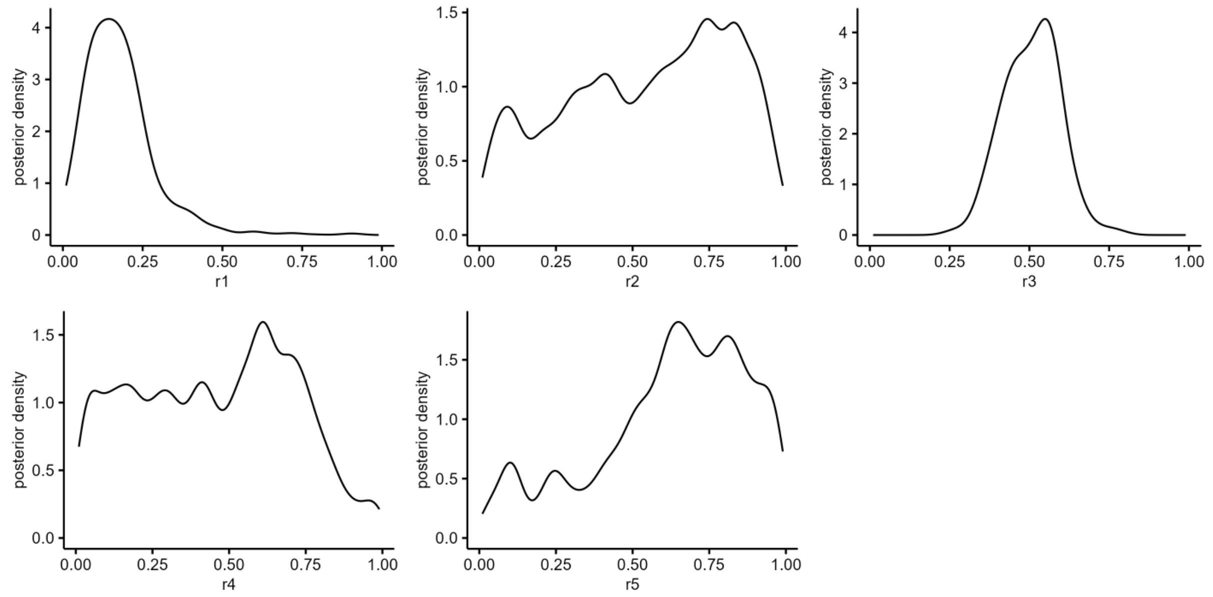

Figure S33. Posterior probability distributions for admixture rate parameters from the best supported scenario in global model set 22.

Appendix S10: Map of dwarf birch Quaternary population history

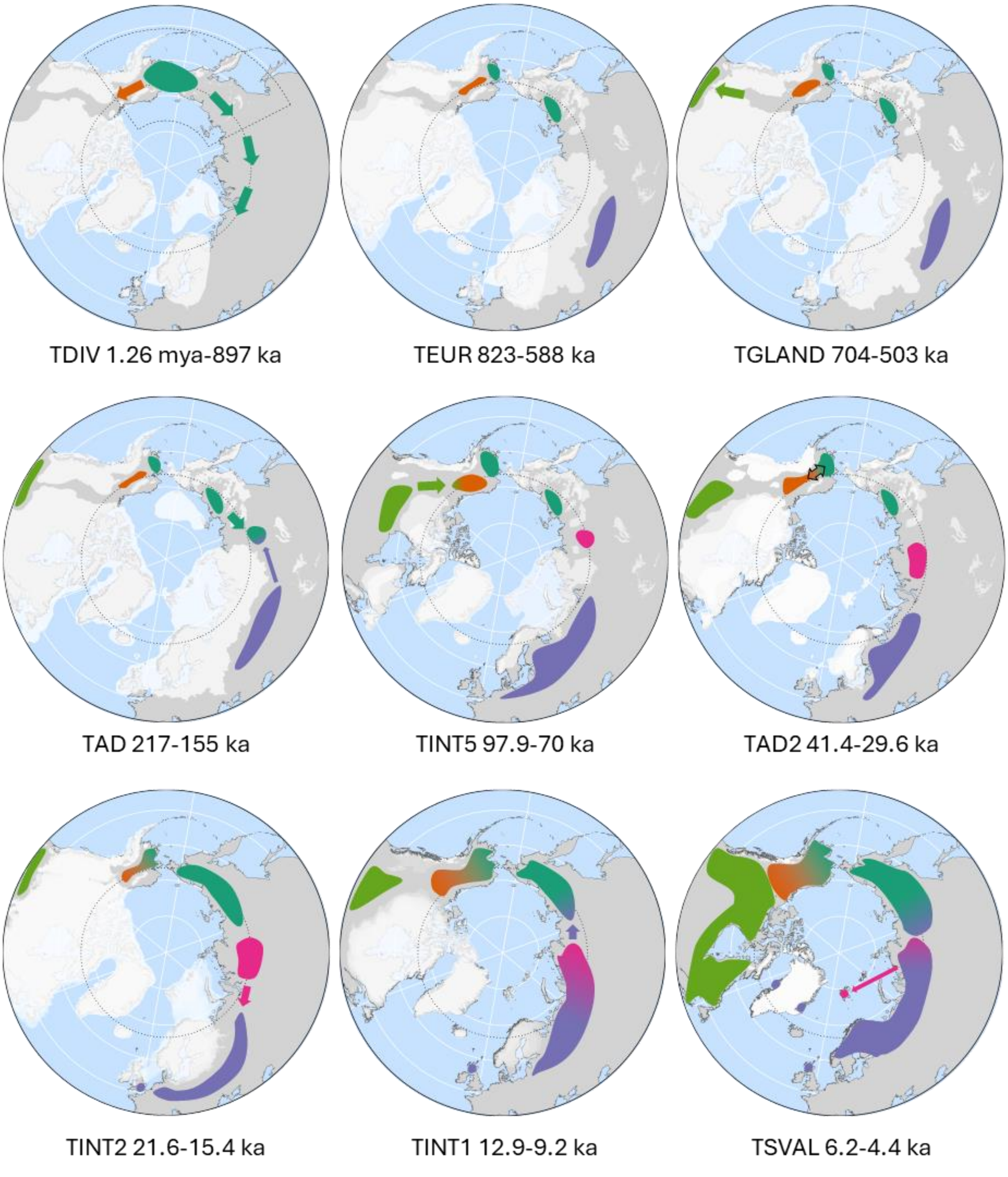

Figure S34. Maps of inferred locations of key demographic events of dwarf birch in the Quaternary. Hypothesised locations of key demographic events from the best supported coalescent modelling scenario. Unidirectional arrows indicate population migration and divergence, unidirectional arrows with colour gradients in the receiving population represent introgression, bidirectional arrows indicate admixture with gradients showing the resulting genetic change in the focal population. Polygon colours correspond to populations used in coalescent modelling: Exilis (**dark green**), Glandulosa west (**orange**), Glandulosa East (**light green**), Nana (**purple**) and Tundrarum and latterly Svalbard (**pink**). Distributions are

hypothesised and approximate. Ice sheets at each time step are shown, with multiple ice sheets overlaying each other with some transparency, since multiple changes often occurred during each time step. Only the modern configuration of land and sea level is shown due to data availability, although much of Beringia was exposed at various points in the Quaternary; population distributions in Alaska and Eastern Siberia are likely under-estimated.

Appendix S11: A non-toxic DNA extraction protocol from dwarf birch herbarium specimens for next-generation sequencing

CTAB and SDS protocol, modified from Barbier et al. (2019), with additions from Wang et. al. (2013) and Qiagen DNeasy Plant Mini Kit.

Summary: We created an efficient and cheap protocol that can produce large quantities of high-quality genomic DNA from dried dwarf birch samples, and so is suitable for samples collected in remote field campaigns. The DNA produced is of sufficient quality and quantity for next generation sequencing such as restriction site-associated sequencing (RADSeq). The protocol also avoids use of toxic and carcinogenic chemicals (Phenol-chloroform and 2-mercaptoethanol) commonly used in extraction protocols.

Duration: The full protocol takes approximately 9 hours for 24 samples (plus additional optional overnight incubation), with 2 consecutive hours unsupervised time. Although approximately double the time required for a commercial kit extraction, the protocol is relatively fast for a CTAB-based method, where one extraction can otherwise take 2-4 days. The protocol could in theory be used for high-throughput processing of 96 well cluster tube arrays if appropriate centrifuge and rotor are available, but it has not been tested. Care would be needed to ensure DNA pellets are not accidentally discarded.

Reagents

- 1 M Tris hydrochloride (Tris-HCl) buffer, pH 8.0 (1 M Tris-HCl pH3.0-5.0, adjust to pH 8.0 using 10N NaOH, fill to final volume with deionised H_2O)
- 0.5 M Ethylenediaminetetraacetic acid (EDTA) buffer, pH 8.0 (0.5 M EDTA, adjust to pH 8 using 10N NaOH, fill to final volume with deionised H_20)
- Sorbitol buffer (Inglis et al. 2018) (0.35 M sorbitol, 100 mM of 1M TRIS-HCl buffer pH8, 5 mM EDTA buffer, Polyvinylpyrrolidone (PVP40) 1% w/v (weight by volume, i.e. the mass per 100 ml solution), fill to final volume with deionised H_2O)
- Cetrimonium bromide (CTAB) buffer (Barbier et al. 2019) (CTAB 2% w/v, 1.4 M NaCl, 0.5 M EDTA buffer pH 8, 1 M TRIS-HCl buffer pH 8, PVP40 2% w/v, fill to final volume with deionised H_2O)
- TE buffer (10 mM of 1 M TRIS-HCl buffer pH 8.0, 1 mM of 0.5 M EDTA buffer
- NaCl solution (5 M NaCl, fill to final volume with deionised H_2O)
- Isopropanol (100%)
- Ethanol (70%, fill to final volume with deionised H_2O)
- AW1, AW2, and AE buffers from DNeasy Plant Mini Kit (catalogue no. 69104; QIAGEN, Hilden, Germany). See protocol for generic alternatives.

- Proteinase K (any brand)
- RNase A (any brand)
- SDS (sodium dodecyl sulfate, 10% w/v)

Equipment and consumables

- 2ml tubes for freezing and grinding stages (Safe-Lock Tubes 2.0mL, catalogue no. 0030120230; Eppendorf, Hamburg, Germany). Alternatives include cryotubes to withstand low temperatures.
- 3mm Tungsten beads
- Liquid nitrogen
    - N.B Use of liquid N is optional if plants are dried. We retained the liquid N step to minimise further DNA degradation as it was suspected that samples could have been subject to degradation resulting from collection or storage conditions. A less costly alternative to liquid N would be to carefully freeze and store tissue at -80°C, when possible, then disrupt with a) a bead mill, or b) grind with a prechilled mortar and pestle (Sahu et al., 2012). As sample heating occurs during disruption with a bead mill, refreezing the samples for 30 minutes between cycles may reduce DNA degradation.
- Bead mill (TissueLyser II and adaptor set 2 x 24; catalogue no.s 85300, 69982; QIAGEN, Hilden, Germany)
- Vortex mixer
- Heating block (water bath can also be used)
- Microcentrifuge (5425 R, catalogue no.5406000569; Eppendorf, Hamburg, Germany)
- White spin columns from DNeasy Plant Mini Kit (catalogue no. 69104; QIAGEN, Hilden, Germany). Less costly alternatives include EconoSpin All-In-One Silica Membrane Mini Spin Column (catalogue no. 1920-050/250; Epoch Life Sciences, Missouri City, Texas, USA), as used by Mavrodiev et al. (2021).
- Open bore pipettes for later stages in the extraction protocol, to prevent damage and shearing of DNA during pipetting.

Equipment used for DNA quantification and quality assessment:

- Spectrophotometer (Nanodrop One, catalogue no. ND-ONE-W; Thermo Scientific, Waltham, Massachusetts, United States)
- Fluorometer (Qubit 4 Fluorometer, dsDNA Broad Range Assay kit, and assay tubes; Catalogue no.s Q33226, Q32850, and Q32856; Invitrogen, Waltham, Massachusetts, USA)
- Gel electrophoresis apparatus with 1% TAE agarose gel, ethidium bromide, and UV light box
- DNA ladders: FastRuler High Range DNA Ladder (catalogue no. SM1123; Thermo Scientific Waltham, Massachusetts, United States), GeneRuler 1 kb DNA Ladder (catalogue no. SM0311; Thermo Scientific Waltham, Massachusetts, United States)

Protocol

Part I: Tissue disruption and sorbitol wash

1. Place 20 mg of dried leaf tissue in a 2ml tube or cryotube with a 3mm tungsten bead. 100-150 mg may be required for fresh tissue. Flash-freeze with liquid nitrogen for 2 minutes and transfer to -80°C freezer for storage.
2. Use TissueLyser adaptors that have been stored at -80°C. Disrupt tissues in Tissuelyser at 28 hertz for 1 minute. Rotate alignment of plates 180° and disrupt for a further 1 minute.
    a. N.B keeping the sample and equipment as cold as possible reduces nuclease activity and chance of DNA degradation. Browning of tissue during extraction indicates DNA degradation (Sahu et al., 2012)
    b. N.B if tubes crack during disruption, use stronger cryotubes or lower frequency (e.g. 23 hertz). We found that if tubes were not stored at -80°C overnight, but used immediately after liquid nitrogen step, 2ml SafeLock Eppendorf tubes cracked.
3. To remove polysaccharides, add 1 ml Sorbitol buffer. Briefly vortex samples and inspect to confirm suspension of powdered material.
    a. N.B Ensure all tissue powder is in contact with liquid using pipette tips.
    b. N.B Store samples on ice until CTAB and proteinase K are added.
4. Centrifuge tubes at 5,000 x g (relative centrifugal force) for 5 minutes at room temperature (RT).
5. Carefully uptake supernatant with pipette
    c. N.B repeat sorbitol addition and centrifuge steps if the supernatant from the first wash is viscous, turbid, or dark in colour (Inglis et al., 2018).
6. Turn on heat block to 65°C.

Part II: Cell lysis and phase separation

7. Add 800 µL CTAB buffer and 20µL proteinase K and mix by inversion.
    a. N.B minimise mechanical damage of DNA by inverting rather than vortexing the DNA from this stage.
8. Incubate at 65°C for 1 hr. Mix by inversion every 15 mins.
    a. N.B An incubation temperature of 65°C irreversibly denatures nucleases.
    b. N.B the beads in the tubes help with the macerating process, ensuring the plant material is in contact with the buffer.
9. Add 50 µL of SDS 10% and mix by inversion.
    a. N.B SDS precipitates with CTAB-polysaccharide complexes, making the sample cloudy.
10. Centrifuge at 20,000 x g for 15 minutes at RT. Turn on heating block to 37°C and cool plates in running water if necessary.
    a. N.B Centrifugation and pelleting of un-lysed leaf material and tissue debris reduces the continued leeching of leaf phenolics into solution.
11. Remove carefully from the centrifuge, uptake, and transfer 600 µL of the liquid phase into a new 2 ml tube.
    a. CTAB/SDS complexes will form a semi-solid film at the surface, so push the pipette tip past this to uptake the liquid below (but also avoid uptake of the cell debris pellet at the bottom of the tube).
12. Add 4µL RNase A and incubate at 37°C for 30 minutes.
13. Centrifuge at 20,000 x g for 15 minutes at RT.

14. Uptake and transfer 600 µL of supernatant from each sample into new 2ml tube. Avoid uptake of any remaining pelleted material at the bottom.

Part III: Nucleic acid precipitation and elution

15. Add 420 µL (0.7 x volume) of pre-cooled (-20°C) isopropanol and 300µL (half volume) pre-cooled 5M NaCl solution and mix by inversion.
    a. N.B NaCl promotes precipitation by neutralising the charge on the nuclei acid backbone, while polysaccharides stay in solution.
    b. N.B lower temperatures promotes better pellet formation of nucleic acids.
16. Leave tubes at -20°C for 1 hour.
    a. N.B the 1 hour incubation time should not be exceeded as NaCl will eventually precipitate, preventing the DNA pellet from forming during centrifugation (Healey et al., 2014).
17. Centrifuge at 20,000 x g for 1 hour at 4°C.
18. Remove isopropanol carefully with pipette, avoiding the pellet which will be glassy and difficult to see at this point.
19. Add 700 µL pre-cooled (-20°C) 70% ethanol and mix by inversion.
    a. N.B Ethanol removes excess salt from previous step, while polysaccharides, phenols, and proteins remain soluble.
20. Centrifuge at 20,000 x g for 15 mins at 4°C.
21. Remove ethanol carefully with pipette, centrifuge briefly (1 minute), and remove remaining ethanol.
    a. N.B. Additional ethanol wash, centrifuge, and removal step with 100% ethanol can be added here if not using spin columns. Ensure that all the ethanol is removed before proceeding to the next step, e.g. by air-drying the samples for some minutes.
22. Add 200 µL pre-warmed TE buffer. Gently pipette the solution up and down to resuspend the pellet but avoid uptake of pellet.
23. Incubate samples at 65°C for 20 minutes.
    a. N.B. This speeds up the resuspension and redissolving of the pellet. Leaving samples overnight at 4°C or RT will ensure the pellet has completely redissolved before further stages.

Part IV: Further DNA separation and purification using spin columns.

If purity ratios of the DNA extracts determined using a spectrophotometer are unacceptable (A260/280 <1.8, A260/230 <2), an additional stage using silica membrane spin columns to purify the DNA and remove polysaccharide contamination can be carried out. An indication whether this is necessary is the colour of the DNA pellet after step (17). If the DNA pellet is not pure white but discoloured yellow or green then the spin column step is necessary. Polysaccharide contamination in the extracted DNA solution after step (18) will be indicated by a viscous, glue-like solution (Porebski et al., 1997; Souza et al., 2012) that is difficult to pipette. Note that spin columns may reduce the yield of DNA (Healey et al 2014). The spin columns may be sufficient so that alcohol precipitation is not necessary, as in Mavrodiev et al (2021), but this was not tested.

24. Add 250µl (1.5 x volume) of buffer AW1 (or an alternative high-salt TNE buffer; TRIS-HCL, NaCl, and EDTA). Mix by pipetting.

a. N.B The buffer promotes DNA binding to the spin column by maintaining pH at an optimal level.
b. N.B. Using wide bore tips may reduce DNA shearing.

25. Pipette all liquid into spin columns placed in collection tubes.
26. Centrifuge at 6000 x g for 1 minute at RT. Discard flow through and collection tube, keeping spin column.
27. Place spin column in new collection tube. Add 500 µL AW2 (or similar ethanol-containing wash solution).
28. Centrifuge at 6000 x g for 1 minute at RT. Discard flow through.
    a. N.B DNA remains bound to the membrane while contaminants are washed away.
29. Add another 500µL AW2 and centrifuge at 20,000 x g for 2 minutes. Discard flowthrough.
30. Remove spin column carefully, ensuring flowthrough does not touch spin column. Transfer to new 2ml tube.
31. Add 50µL AE[1] (or TE buffer; 10 mM Tris-HCl pH 9 and 0.5 mM EDTA pH 9). Incubate for 5 minutes at RT.
32. Centrifuge at 6,000 x g for 1 minute at RT.
33. Repeat steps (27) and (28) and then remove spin column and store DNA samples at 4°C, or at -20°C for longer storage periods.

Notes on reagents and extraction conditions

- CTAB is a cationic detergent that captures lipids of cell membranes, frees contents of inner cell, and promotes separation of proteins and polysaccharides from nucleic acids. At high ionic strength (i.e. when the buffer contains higher concentrations of CTAB and NaCl), CTAB bind to polysaccharides and forms complexes which are removed. CTAB also denatures proteins and enzymes including deoxyribonuclease DNase which degrades DNA (Heikrujam et al., 2020).
- NaCl in the CTAB buffer prevents formation of CTAB-nucleic acid complexes. Note that lower salt concentrations (<0.5 M) DNA is insoluble, while at higher salt concentrations polysaccharides are insoluble (Sahu et al., 2012).
- EDTA chelates divalent cations which are used as cofactors for nucleases, thus reducing the activity of nucleases which could degrade the DNA (Heikrujam et al., 2020).
- Using a TRIS-HCl buffer at pH 8.0 helps maintains extraction pH at a level which promotes DNA stability and reduces DNA degradation (Heikrujam et al., 2020).
- PVP 40 prevent polyphenols present in plant tissue from oxidising and binding to DNA (indicated by a browning of the extracts), reducing the likelihood of polyphenols co-precipitating with DNA and making downstream processing difficult and reducing DNA stability (Heikrujam et al., 2020; Sahu et al., 2012).
- SDS is a detergent which helps digest the cell membrane and the release of more nucleic acids, while separating proteins from nucleic acids (Barbier et al., 2019).

References

Alexander, D. H., & Lange, K. (2011). Enhancements to the ADMIXTURE algorithm for individual ancestry estimation. *BMC Bioinformatics*, *12*(1), 246. https://doi.org/10.1186/1471-2105-12-246

Alexander, D. H., Novembre, J., & Lange, K. (2009). Fast model-based estimation of ancestry in unrelated individuals. *Genome Research*, *19*(9), 1655–1664. https://doi.org/10.1101/gr.094052.109

Alsos, I. G., Engelskjøn, T., & Brochmann, C. (2002). Conservation genetics and population history of Betula nana, Vaccinium uliginosum, and Campanula rotundifolia in the Arctic archipelago of Svalbard. *Arctic, Antarctic, and Alpine Research*, *34*(4), 408–418. https://doi.org/10.1080/15230430.2002.12003511

Barbier, F. F., Chabikwa, T. G., Ahsan, M. U., Cook, S. E., Powell, R., Tanurdzic, M., & Beveridge, C. A. (2019). A phenol/chloroform-free method to extract nucleic acids from recalcitrant, woody tropical species for gene expression and sequencing. *Plant Methods*, *15*(1), 62. https://doi.org/10.1186/s13007-019-0447-3

Bartley, D. D. (2019). *King's Pool pollen dataset* (1.0, p. 24 KB) [JSON]. Neotoma Paleoecological Database. https://doi.org/10.21233/SHHZ-0F61

Bartley, D. D., & Morgan, A. V. (1990). The palynological record of the King's Pool, Stafford, England. *New Phytologist*, *116*(1), 177–194. https://doi.org/10.1111/j.1469-8137.1990.tb00522.x

Borrell, J. S., Wang, N., Nichols, R. A., & Buggs, R. J. A. (2018). Genetic diversity maintained among fragmented populations of a tree undergoing range contraction. *Heredity*, *121*(4), 304. https://doi.org/10.1038/s41437-018-0132-8

Catchen, J., Amores, A., Hohenlohe, P., Cresko, W., & Postlethwait, J. H. (2011). Stacks: Building and Genotyping Loci De Novo From Short-Read Sequences. *G3 Genes|Genomes|Genetics*, *1*(3), 171–182. https://doi.org/10.1534/g3.111.000240

Catchen, J., Hohenlohe, P. A., Bassham, S., Amores, A., & Cresko, W. A. (2013). Stacks: An analysis tool set for population genomics. *Molecular Ecology*, *22*(11), 3124–3140. https://doi.org/10.1111/mec.12354

Chang, C. C., Chow, C. C., Tellier, L. C., Vattikuti, S., Purcell, S. M., & Lee, J. J. (2015). Second-generation PLINK: Rising to the challenge of larger and richer datasets. *GigaScience*, *4*(1), s13742-015-0047–0048. https://doi.org/10.1186/s13742-015-0047-8

Collin, F.-D., Durif, G., Raynal, L., Lombaert, E., Gautier, M., Vitalis, R., Marin, J.-M., & Estoup, A. (2021). Extending approximate Bayesian computation with supervised machine learning to infer demographic history from genetic polymorphisms using DIYABC Random Forest. *Molecular Ecology Resources*, *21*(8), 2598–2613. https://doi.org/10.1111/1755-0998.13413

Cornuet, J.-M., Pudlo, P., Veyssier, J., Dehne-Garcia, A., Gautier, M., Leblois, R., Marin, J.-M., & Estoup, A. (2014). DIYABC v2.0: A software to make approximate Bayesian computation inferences about population history using single nucleotide polymorphism, DNA sequence and microsatellite data. *Bioinformatics*, *30*(8), 1187–1189. https://doi.org/10.1093/bioinformatics/btt763

de Groot, W. J., Thomas, P. A., & Wein, R. W. (1997). Betula Nana L. and Betula Glandulosa Michx. *Journal of Ecology*, *85*(2), 241–264. https://doi.org/10.2307/2960655

Eidesen, P. B., Alsos, I. G., & Brochmann, C. (2015). Comparative analyses of plastid and AFLP data suggest different colonization history and asymmetric hybridization between Betula pubescens and B. nana. *Molecular Ecology*, *24*(15), 3993–4009. https://doi.org/10.1111/mec.13289

Elven, R., Murray, D., Razzhivin, V., & Yurtsev, B. (2011). *Annotated Checklist of the Panarctic Flora (PAF) Vascular plants version 1.0*. Checklist of the Panarctic Flora (PAF). http://panarcticflora.org/

Fyfe, R. M., de Beaulieu, J.-L., Binney, H., Bradshaw, R. H. W., Brewer, S., Le Flao, A., Finsinger, W., Gaillard, M.-J., Giesecke, T., Gil-Romera, G., Grimm, E. C., Huntley, B., Kunes, P., Kühl, N., Leydet, M., Lotter, A. F., Tarasov, P. E., & Tonkov, S. (2009). The European Pollen Database: Past efforts and current activities. *Vegetation History and Archaeobotany*, *18*(5), 417–424. https://doi.org/10.1007/s00334-009-0215-9

Giesecke, T., Davis, B., Brewer, S., Finsinger, W., Wolters, S., Blaauw, M., De Beaulieu, J.-L., Binney, H., Fyfe, R. M., Gaillard, M.-J., Gil-Romera, G., Van Der Knaap, W. O., Kuneš, P., Kühl, N., Van Leeuwen, J. F. N., Leydet, M., Lotter, A. F., Ortu, E., Semmler, M., & Bradshaw, R. H. W. (2014). Towards mapping the late Quaternary vegetation change of Europe. *Vegetation History and Archaeobotany*, *23*(1), 75–86. https://doi.org/10.1007/s00334-012-0390-y

Goudet, J., Jombart, T., Kamvar, Z. N., Archer, E., & Hardy, O. (2022). *hierfstat: Estimation and Tests of Hierarchical F-Statistics* (0.5-11) [Computer software]. https://CRAN.R-project.org/package=hierfstat

Healey, A., Furtado, A., Cooper, T., & Henry, R. J. (2014). Protocol: A simple method for extracting next-generation sequencing quality genomic DNA from recalcitrant plant species. *Plant Methods*, *10*(1), 21. https://doi.org/10.1186/1746-4811-10-21

Heikrujam, J., Kishor, R., & Mazumder, P. B. (2020). The Chemistry Behind Plant DNA Isolation Protocols. In *Biochemical Analysis Tools—Methods for Bio-Molecules Studies*. IntechOpen. https://doi.org/10.5772/intechopen.92206

Inglis, P. W., Pappas, M. de C. R., Resende, L. V., & Grattapaglia, D. (2018). Fast and inexpensive protocols for consistent extraction of high quality DNA and RNA from challenging plant and fungal samples for high-throughput SNP genotyping and sequencing applications. *PLOS ONE*, *13*(10), e0206085. https://doi.org/10.1371/journal.pone.0206085

Malinsky, M., Trucchi, E., Lawson, D. J., & Falush, D. (2018). RADpainter and fineRADstructure: Population inference from RADseq data. *Molecular Biology and Evolution*, *35*(5), 1284–1290. https://doi.org/10.1093/molbev/msy023

Porebski, S., Bailey, L. G., & Baum, B. R. (1997). Modification of a CTAB DNA extraction protocol for plants containing high polysaccharide and polyphenol components. *Plant Molecular Biology Reporter*, *15*(1), 8–15. https://doi.org/10.1007/BF02772108

Purcell, S., & Chang, C. (2022). *PLINK* (1.9) [Computer software]. www.cog-genomics.org/plink/1.9/

R Development Core Team. (2020). *R: A language and environment for statistical computing* (4.0.3) [Computer software]. R Foundation for Statistical Computing. www.R-project.org

Rochette, N. C., Rivera-Colón, A. G., & Catchen, J. M. (2019). Stacks 2: Analytical methods for paired-end sequencing improve RADseq-based population genomics. *Molecular Ecology*, *28*(21), 4737–4754. https://doi.org/10.1111/mec.15253

Sahu, S. K., Thangaraj, M., & Kathiresan, K. (2012). DNA Extraction Protocol for Plants with High Levels of Secondary Metabolites and Polysaccharides without Using Liquid

Nitrogen and Phenol. *ISRN Molecular Biology*, *2012*, 205049. https://doi.org/10.5402/2012/205049

Souza, H. A. V., Muller, L. A. C., Brandão, R. L., & Lovato, M. B. (2012). Isolation of high quality and polysaccharide-free DNA from leaves of Dimorphandra mollis (Leguminosae), a tree from the Brazilian Cerrado. *Genetics and Molecular Research*, *11*(1), 756–764. https://doi.org/10.4238/2012.March.22.6

Touchette, L., Godbout, J., Lamothe, M., Porth, I., & Isabel, N. (2024). A cryptic syngameon within Betula shrubs revealed: Implications for conservation in changing subarctic environments. *Evolutionary Applications*, *17*(4), e13689. https://doi.org/10.1111/eva.13689

Wang, N., Thomson, M., Bodles, W. J. A., Crawford, R. M. M., Hunt, H. V., Featherstone, A. W., Pellicer, J., & Buggs, R. J. A. (2013). Genome sequence of dwarf birch (Betula nana) and cross-species RAD markers. *Molecular Ecology*, *22*(11), 3098–3111. https://doi.org/10.1111/mec.12131

Williams, J. W., Grimm, E. C., Blois, J. L., Charles, D. F., Davis, E. B., Goring, S. J., Graham, R. W., Smith, A. J., Anderson, M., Arroyo-Cabrales, J., Ashworth, A. C., Betancourt, J. L., Bills, B. W., Booth, R. K., Buckland, P. I., Curry, B. B., Giesecke, T., Jackson, S. T., Latorre, C., … Takahara, H. (2018). The Neotoma Paleoecology Database, a multiproxy, international, community-curated data resource. *Quaternary Research*, *89*(1), 156–177. https://doi.org/10.1017/qua.2017.105
